# Targeting oxidized phospholipids by AAV-based gene therapy in mice with established hepatic steatosis prevents progression to fibrosis

**DOI:** 10.1126/sciadv.abn0050

**Published:** 2022-07-15

**Authors:** Clint M. Upchurch, Scott Yeudall, Caitlin M. Pavelec, Dennis Merk, Jan Greulich, Mohan Manjegowda, Shyam S. Raghavan, Irina M. Bochkis, Michael M. Scott, Edward Perez-Reyes, Norbert Leitinger

**Affiliations:** ^1^ Department of Pharmacology, University of Virginia School of Medicine, Charlottesville, VA 22904, USA.; ^2^ Robert M. Berne Cardiovascular Research Center, University of Virginia School of Medicine, Charlottesville, VA 22904, USA.; ^3^ Environmentally-Induced Cardiovascular Degeneration, Clinical Chemistry and Laboratory Diagnostics, Medical Faculty, University Hospital and Heinrich-Heine University Düsseldorf, 40225 Düsseldorf, Germany.; ^4^ IUF-Leibniz Research Institute for Environmental Medicine, 40225 Düsseldorf, Germany.; ^5^ Department of Pathology, University of Virginia, Charlottesville, VA 22904, USA.

## Abstract

Oxidized phosphatidylcholines (OxPCs) are implicated in chronic tissue damage. Hyperlipidemic LDL-R-–deficient mice transgenic for an OxPC-recognizing IgM fragment (scFv-E06) are protected against nonalcoholic fatty liver disease (NAFLD). To examine the effect of OxPC elimination at different stages of NAFLD progression, we used cre-dependent, adeno-associated virus serotype 8–mediated expression of the single-chain variable fragment of E06 (AAV8-scFv-E06) in hepatocytes of albumin-cre mice. AAV8-induced expression of scFv-E06 at the start of FPC diet protected mice from developing hepatic steatosis. Independently, expression of scFv-E06 in mice with established steatosis prevented the progression to hepatic fibrosis. Mass spectrometry–based oxophospho-lipidomics identified individual OxPC species that were reduced by scFv-E06 expression. In vitro, identified OxPC species dysregulated mitochondrial metabolism and gene expression in hepatocytes and hepatic stellate cells. We demonstrate that individual OxPC species independently affect disease initiation and progression from hepatic steatosis to steatohepatitis, and that AAV-mediated expression of scFv-E06 is an effective therapeutic intervention.

## Teaser

AAV8-induced scFv-E06 expression protects mice from developing hepatic steatosis and, independently, progression to fibrosis.

## INTRODUCTION

Nonalcoholic fatty liver disease (NAFLD) is a multistage disease that affects approximately 30% of the global population (*1*, *2*). Hepatic steatosis is the hallmark of NAFLD, which, in a subset of patients, will progress into nonalcoholic steatohepatitis (NASH). Steatosis can arise as a result of caloric overload, which dysregulates hepatocyte bioenergetics and metabolism (*3*) and increases reactive oxygen species (*4*) and hepatic triglyceride accumulation (*5*, *6*), resulting in organ damage indicated by elevated plasma alanine aminotransferase (ALT) and aspartate aminotransferase (AST) (*4*, *7*–*11*). The culmination of multiple hepatic insults leads to the development of NASH, which is characterized by inflammation (*12*) and activation of hepatic stellate cells, ultimately leading to irreversible hepatic fibrosis (*12*–*17*). In the absence of U.S. Food and Drug Administration–approved pharmacological interventions and suitable biomarkers for NASH, there is an urgent need for new therapeutic and diagnostic tools (*18*).

Excess radical oxygen species generation in steatotic livers leads to the formation of lipid oxidation products, including oxidized phosphatidylcholines (OxPCs) (*19*–*22*). Free radical–driven and enzymatically driven oxidation of polyunsaturated fatty acids contained in phospholipids forms chemically unique classes of oxidized species whose location and chemical functionalization dictate the regulation of specific cellular responses, including endothelial barrier integrity (*23*–*27*); immune cell migration (*28*), activation (*19*, *20*), and metabolism (*29*); bone homeostasis (*30*); and regulated cell death (*31*, *32*). Consequently, OxPCs are thought to play a central role in acute pathologies such as sepsis (*21*) and lung injury (*23*, *24*, *33*), as well as in chronic diseases including those of the metabolic syndrome (*34*, *35*). Moreover, plasma levels of OxPCs, as measured by reactivity with E06, a natural immunoglobulin M (IgM) that binds oxidized phosphorylcholine (*36*), predict severity of human carotid and femoral atherosclerosis (*37*).

Recent work from the Witztum laboratory has demonstrated that constitutive transgenic expression of a single-chain variable fragment of E06 (scFv-E06) protects hypercholesterolemic *Ldlr^−/−^* mice from diet-induced hepatic steatosis and subsequent NASH (*35*, *37*, *38*). While these studies demonstrated that targeting OxPCs in general is sufficient to improve clinical outcomes in a mouse model of chronic disease, the identity of individual OxPC species that are eliminated by scFv-E06 in vivo remains unknown. Furthermore, it is unknown whether OxPC sequestration by scFv-E06 is sufficient to independently halt the progression to NASH and the transition to hepatic fibrosis, and it is necessary to identify the cellular targets and the pathological mechanisms by which OxPCs drive hepatic steatosis and fibrosis.

Here, we show that adeno-associated virus serotype 8–mediated hepatic expression of scFv-E06 (AAV8-E06) eliminates defined plasma OxPC species derived from oxidation of 1-palmitoyl-2-arachidonyl-*sn*-glycero-3-phosphocholine (PAPC) and 1-palmitoyl-2-linoleoyl-*sn*-glycero-3-phosphocholine (PLPC), which protects mice from diet-induced hepatic steatosis. Identified OxPC species regulate hepatocyte gene expression and shift cellular metabolism toward a bioenergetically impaired state, which results in reduced oxygen consumption and increased lipid droplet accumulation. Moreover, intervention with AAV8-scFv-E06 in mice with established hepatic steatosis prevents the progression to NASH and hepatic fibrosis. OxPC species that were reduced during the progression phase regulate hepatic stellate cell bioenergetics and gene expression. Together, we identify specific pathology-driving OxPC species in plasma that may be used as noninvasive biomarkers to diagnose distinct stages of NAFLD, and we demonstrate efficacy of AAV8-mediated gene transfer of scFv-E06 as an intervention-based therapeutic measure that attenuates the initiation of hepatic steatosis and the progression to fibrotic steatohepatitis in mice.

## RESULTS

### AAV8-mediated gene transfer for cre recombinase–dependent expression of scFv-E06 in mice

**Fig. 1.**
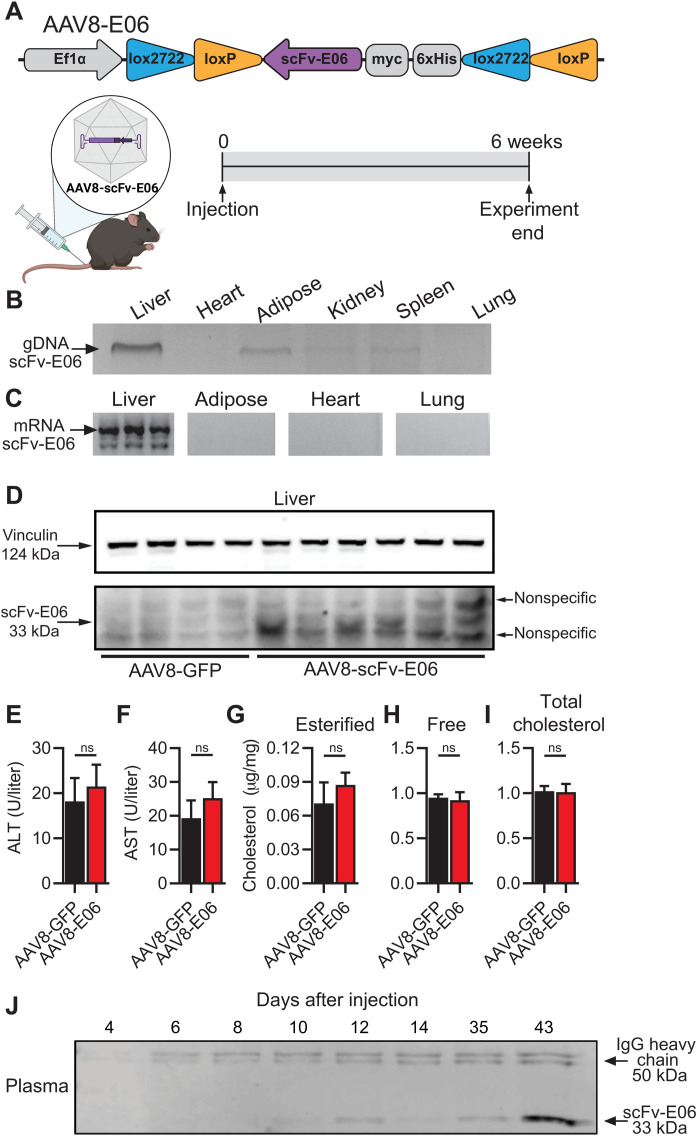
Virus-mediated gene transfer for cre-dependent expression of scFv-E06 in a murine model. (**A**) Schematic of experimental design. Mice were injected via tail vein with AAV8-E06 or AAV-GFP and fed chow diet for 6 weeks (created with BioRender.com). Viral transduction of AAV8-scFv-E06 was assessed by (**B**) polymerase chain reaction (PCR) of genomic DNA and (**C**) reverse transcription PCR of mRNA. scFv-E06 expression in the (**D**) liver was confirmed via Western blotting 6 weeks after injection. Vinculin was used as a loading control. There were no significant changes in (**E**) ALT, (**F**) AST, and (**G**) esterified, (**H**) free, and (**I**) total liver cholesterol between scFv-E06– and GFP-expressing mice (AAV8-GFP, *n* = 5; AAV8-E06, *n* = 6). (**J**) scFv-E06 was detectable in plasma 12 days after injection and remained elevated after 6 weeks. Nonspecific IgG heavy chain staining was used as a loading control. ns, not significant.

Previous reports demonstrated that constitutive transgenic expression of scFv-E06 via the *Apoe* promoter protected *Ldlr^−/−^* mice fed a high-cholesterol diet from hepatic steatosis and ensuing steatohepatitis (*35*, *38*). To investigate the effect of scFv-E06 at different stages of disease progression and to establish a therapeutic approach using virus-mediated gene transfer of scFv-E06, we developed an AAV8 construct containing a myc- and 6xHis-tagged scFv-E06 flanked by double-inverse orientation flox sites (AAV8-scFv-E06) for cre-dependent expression (Fig. 1A). Speer6-ps1^Tg(Alb-cre)21Mgn^/J (Alb-cre) mice, which express cre recombinase specifically in hepatocytes, were injected via tail vein with AAV8-scFv-E06 or a control AAV8 expressing green fluorescent protein (AAV8-GFP). Viral transduction resulted in incorporation of the scFv-E06 vector predominantly in the liver and, to some extent, in adipose tissue, kidney, and spleen, while it was not detected in the heart or lungs (Fig. 1B). Messenger RNA and protein expression of scFv-E06 was restricted to the liver, demonstrating that expression was dependent on cre recombinase (Fig. 1, C and D). scFv-E06 protein was secreted into the plasma with detectable levels as early as 10 days after AAV administration (Fig. 1J).

AAV8-mediated hepatic expression of scFv-E06 had no observable effect on total body weight, and liver, gonadal adipose tissue, heart, kidney, or spleen mass (fig. S1). There were also no differences in levels of ALT (Fig. 1E) and AST (Fig. 1F) in plasma, or esterified (Fig. 1G), free (Fig. 1H), and total cholesterol (Fig. 1I) in livers of mice given AAV8-scFv-E06 compared to AAV-GFP controls. Together, these data demonstrate that AAV8-mediated hepatic expression of scFv-E06 in mice leads to accumulation of scFv-E06 protein in the liver and plasma without inducing overt physiological changes.

Using a high fructose, palmitate, and cholesterol (FPC) diet supplemented with 4.2% sugar water (55/45 glucose/fructose), we establish a progressive model NAFLD defined by distinct stages of hepatic steatosis and subsequent inflammation and fibrosis (*39*). These stages were evident in histopathological assessment of livers from mice fed FPC diet compared to chow-fed controls (fig. S1, G to J). This allowed us to assess the impact of scFv-E06 expression at different stages of NAFLD progression.

### AAV8-mediated expression of scFv-E06 protects mice from diet-induced hepatic steatosis and reduces oxidized phospholipids in plasma

**Fig. 2.**
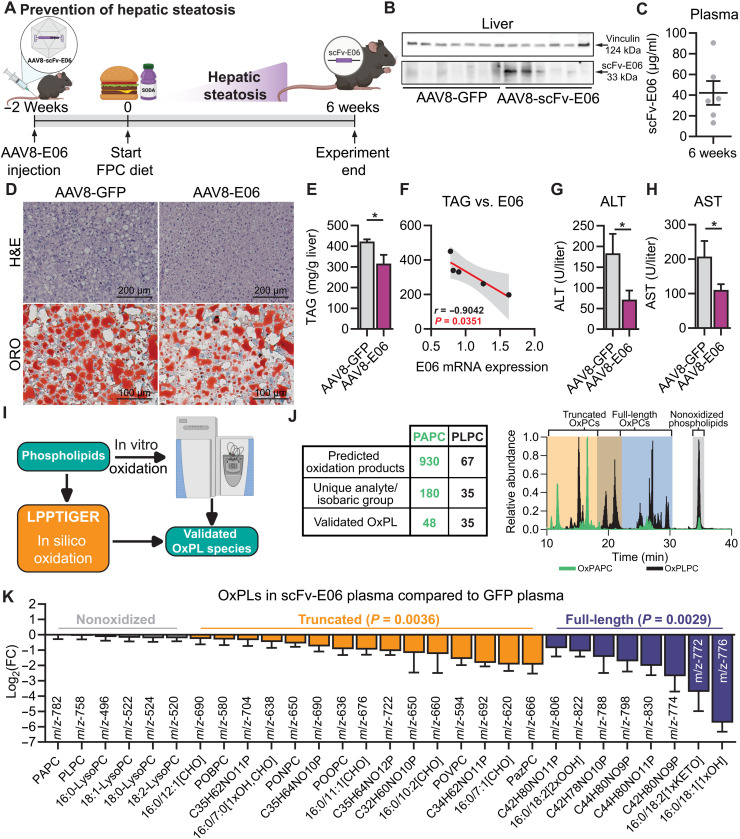
Virus-mediated hepatic expression of scFv-E06 prevented development of diet-induced hepatic steatosis. (**A**) Schematic of experimental design. Mice were injected via tail vein with AAV8-E06 or AAV8-GFP 2 weeks before the start of 6-week FPC diet challenge (created with BioRender.com). (**B**) scFv-E06 protein expression in mouse liver 8 weeks after injection. (**C**) scFv-E06 titer concentrations were estimated in plasma by competitive sandwich enzyme-linked immunosorbent assay (ELISA). (**D**) Hematoxylin and eosin (H&E) and Oil red O (ORO) staining revealed decreased hepatic lipid burden in mice expressing scFv-E06 and was confirmed by (**E**) quantification of hepatic triglycerides (GFP, *n* = 6; scFv-E06, *n* = 5). (**F**) Hepatic triglyceride concentrations negatively correlated with hepatic mRNA expression of scFv-E06 (scFv-E06, *n* = 5). scFv-E06 expression protected mice from diet-induced liver toxicity resulting in significantly lower (**G**) ALT and (**H**) AST (GFP, *n* = 6; scFv-E06, *n* = 6). (**I**) Schema demonstrating strategy for mass spectrometry method development and validation. (**J**) Forty-eight analytes/isobaric groups from PAPC and 35 analytes/isobaric groups from PLPC were validated. (**K**) Both truncated and full-length OxPC species were significantly reduced in mice expressing scFv-E06 (GFP, *n* = 7; scFv-E06, *n* = 6). Statistical significance was determined by two-way analysis of variance (ANOVA), Spearman’s rank correlation, and Student’s *t* test (*P < 0.05).

To determine whether induced expression of scFv-E06 could be used as a therapeutic approach to prevent hepatic steatosis in mice, we inoculated Speer6-ps1^Tg(Alb-cre)21Mgn^/J (Alb-cre) mice via tail vein with AAV8-scFv-E06 or AAV8-GFP 2 weeks before feeding mice FPC diet for 6 weeks to induce hepatic steatosis (Fig. 2A). After 6 weeks, we confirmed the expression of myc-tagged scFv-E06 in the liver by Western blot (Fig. 2B) and mRNA by quantitative reverse transcription polymerase chain reaction (qRT-PCR) in the liver (fig. S2A) and confirmed detectable titers of scFv-E06 in the plasma by enzyme-linked immunosorbent assay (ELISA) (Fig. 2C). scFv-E06–expressing mice exhibited no difference in weight gain compared to GFP-expressing mice over 6 weeks (fig. S2B); however, scFv-E06 expression reduced body fat percentage starting at 4 weeks on FPC diet (fig. S2C). FPC diet feeding increased liver and adipose mass; however, there was no difference in organ mass between GFP- and scFv-E06–expressing mice (fig. S2, D to I). Histological assessment of liver sections by hematoxylin and eosin (H&E) or Oil red O staining revealed that treatment with AAV8-scFv-E06 reduced hepatic tissue damage and lipid accumulation compared to GFP controls (Fig. 2D). Hepatic triglyceride levels were significantly reduced in scFv-E06–expressing mice and negatively correlated with hepatic mRNA expression of scFv-E06 (Fig. 2, E and F). Moreover, plasma ALT (Fig. 2G) and AST (Fig. 2H) levels were significantly decreased in mice expressing scFv-E06, while alkaline phosphatase (ALP), cholesterol, low-density lipoprotein (LDL) and high-density lipoprotein (HDL) cholesterol, plasma triglycerides, albumin, and total protein were unchanged (fig. S2, J to R). These data demonstrate that AAV8-mediated expression of scFv-E06 protects mice from diet-induced hepatic lipid accumulation and liver damage.

Previously published studies have demonstrated that constitutive scFv-E06 expression reduced overall plasma reactivity with IgM E06 (*35*, *38*), indicative of reduced OxPC levels in plasma. However, the identity of OxPC species that are reduced by scFv-E06 expression and the degree of reduction remain unknown.

To identify OxPC species that are affected by scFv-E06 in plasma, we used an in silico platform, LPPTiger (*40*), to predict structures of possible oxidation products that can be generated from oxidation of PAPC and PLPC. We then validated predicted OxPC species by electrospray ionization liquid chromatography–mass spectrometry (ESI–LC-MS) using air- or copper (I) chloride–oxidized PAPC (OxPAPC) and PLPC (OxPLPC) (fig. S3, A and B), which resulted in validation of 48 PAPC- and 35 PLPC-derived oxidized individual analytes or groups of isobaric compounds (Fig. 2, I and J). Next, we assessed the presence of validated compounds in the plasma of GFP- or scFv-E06–expressing mice after 6 weeks on FPC diet (fig. S3, C and D). We identified 23 individual OxPC species and isobaric groups containing OxPCs and 6 nonoxidized PC and lyso-PC species in mouse plasma. Levels of nonoxidized PCs and lyso-PCs were not different in GFP- and scFv-E06–expressing mice; however, all identified OxPC species, including truncated and full-length OxPCs, were markedly decreased in plasma of mice expressing scFv-E06 (Fig. 2K). A similar pattern was observed in the liver; however, the differences in OxPC species were less pronounced between the two groups (fig. S2S).

In the plasma, the truncated and full-length OxPCs that were substantially decreased in scFv-E06–expressing mice included several previously described biologically active compounds containing specific functional groups: Among decreased truncated OxPC species were γ-keto/hydroxy OxPCs [mass/charge ratio (*m/z*) 650] (*22*, *41*), the aldehyde-containing 1-palmitoyl-2-(5′-oxo-valeroyl)-*sn*-glycero-3-phosphocholine (POVPC; *m/z* 594) (*42*), the 4-carbon aldehyde 1-palmitoyl-2-(4′-oxo-butanoyl)-*sn*-glycero-3-phosphocholine (POBPC; *m/z* 580) (*43*), the 8-carbon aldehyde 1-palmitoyl-2-(8′-oxo-octanoyl)-*sn*-glycero-3-phosphocholine (POOPC; *m/z* 636), the 9-carbon aldehyde 1-palmitoyl-2-(9′-oxo-nonanoyl)-*sn*-glycero-3-phosphocholine (*m/z* 650) (*44*, *45*), and the carboxylic acid–containing 1-palmitoyl-2-azelaoyl-*sn*-glycero-3-phosphocholine (PazPC; *m/z* 666) (Fig. 2J) (*45*, *46*). Among the most substantially reduced full-length OxPC were 1-palmitoyl-2-((E)-8′-hydroxyoctadec-12′-enoyl)-*sn*-glycero-3-phosphocholine (HODE-PC; *m/z* 776), an isobaric group with an *m/z* of 798 containing 1-palmitoyl-2-((5E,8E,11E,14E)-4′-hydroxyicosa-5′,8′,11′,14′-tetraenoyl (HETE-PC) (*47*), and an isobaric group with an *m/z* of 830 containing isoprostane-PC (Fig. 2K) (*48*, *49*). The structures of the identified compounds with the corresponding *m/z* values are represented in table S1.

Together, these data demonstrate that AAV8-dependent hepatic expression of scFv-E06 results in its secretion into the plasma, decreased plasma levels of defined OxPC species, and protection from diet-induced hepatic. scFv-E06 showed specificity toward OxPCs, but not nonoxidized phospholipids, recognizing a variety of oxidation-specific functional groups that are associated with previously reported biological functions (*50*, *51*).

### OxPCs regulate hepatocyte gene expression and mitochondrial bioenergetics

**Fig. 3.**
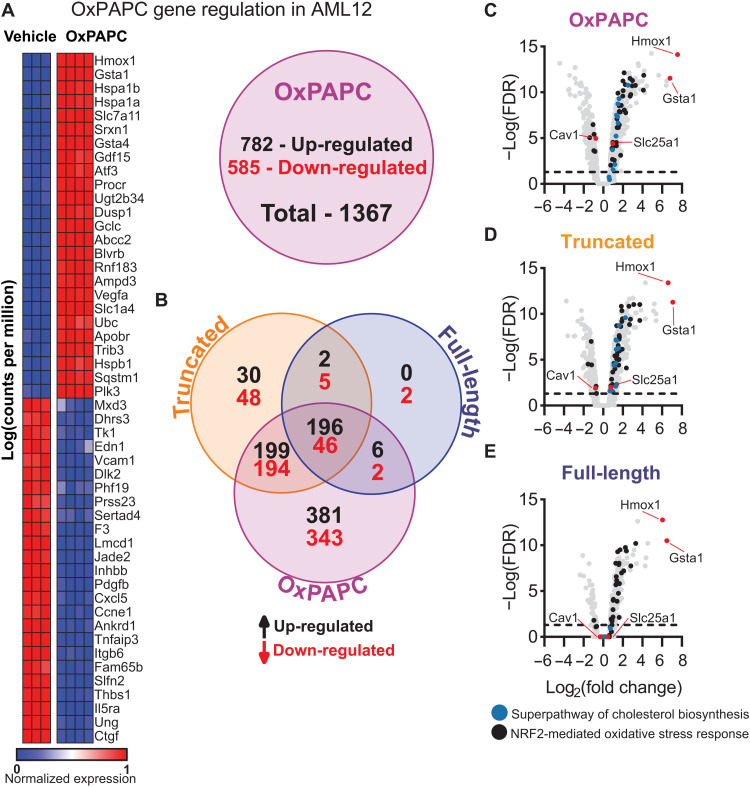
Truncated OxPLs shift AML12 hepatocytes to an anabolic metabolic programming. AML12 hepatocytes were treated with OxPAPC (100 μg/ml) for 4 hours, and gene expression was measured via RNA-seq. (**A**) OxPAPC regulated 1367 genes (782 up-regulated/585 down-regulated, fold change > |1.5| and adjusted *P* < 0.05; vehicle, *n* = 3; OxPAPC; *n* = 4). (**B**) Truncated OxPLs uniquely regulated 78 genes (30 up-regulated/48 down-regulated) compared to 2 down-regulated genes in full-length OxPL and 724 (381 up-regulated/343 down-regulated) in OxPAPC treatment with 242 regulated by all three treatments (196 up-regulated/46 down-regulated) (fold change > |1.5| and adjusted *P* < 0.05, *n* = 3 to 4). Volcano plot analysis of (**C**) OxPAPC, (**D**) truncated OxPAPC, and (**E**) full-length OxPAPC revealed that *Hmox1* and *Gsta1* were the most highly up-regulated genes in all three treatments along with genes associated with the NRF2-mediated oxidative stress response. *Slc25a1* and *Cav1* were regulated in OxPAPC and truncated OxPL treatment, but not in full-length OxPL treatment. The same pattern was observed for genes associated with the superpathway of cholesterol biosynthesis. Statistical significance was determined by one-way ANOVA and Student’s *t* test. Multiple comparisons were corrected by FDR or Dunnett’s multiple comparison correction.

These data imply a role of OxPCs in the development of hepatic steatosis; however, it is unknown if hepatocytes recognize and respond to OxPCs. To investigate whether OxPCs regulate hepatocyte function, we treated a murine hepatocyte cell line (AML12) with a mixture of full-length and truncated OxPCs (OxPAPC) (*42*) for 4 hours and analyzed changes in gene expression by RNA sequencing (RNA-seq). OxPAPC regulated the expression of 1367 genes in AML12 hepatocytes [fold change > 1.5, false discovery rate (FDR) < 0.05], of which 782 were up-regulated and 585 were down-regulated compared to vehicle-treated cells (Fig. 3A and table S2). EnrichR (*52*, *53*) Gene Ontology (GO) pathway analysis revealed that OxPAPC induced pathways associated with oxidative stress, including the “NRF2-mediated oxidative stress response,” the “unfolded protein response,” and the “aryl hydrocarbon receptor signaling pathway,” as well as the “superpathway of cholesterol biosynthesis” (fig. S4A), suggesting that OxPCs contribute to dysregulating cholesterol metabolism, a hallmark of NAFLD (*54*).

Since our data showed that levels of both truncated and full-length OxPCs were decreased by scFv-E06, we separated OxPAPC into two fractions enriched for either truncated or full-length OxPC species using a strong anionic solid phase exchange chromatography method that we previously described (*34*). Treatment of AML12 cells with truncated OxPCs resulted in regulation of 720 genes (427 up-regulated, 293 down-regulated), while full-length OxPCs regulated the expression of 259 (204 up-regulated, 55 down-regulated) genes (Fig. 3B). Of those, truncated OxPCs uniquely regulated 78 genes (30 up/45 down), while full-length OxPCs uniquely down-regulated 2 genes (Fig. 3B and table S2). Both truncated and full-length OxPAPC up-regulated genes associated with oxidative stress such as *Hmox1*, *Gsta1*, *Txnrd1*, *Hspa1a*, and *Hspa1b*, as well as *Ptgs2* (cyclooxygenase 2) (table S2). In addition, qRT-PCR confirmed that full-length OxPCs, truncated OxPCs, and OxPAPC up-regulated the expression of *Hmox1* (fig. S4D) and *Pgd* (fig. S4E), while only truncated OxPCs up-regulated *Acly* (fig. S4F), *Hmgcoas* (fig. S4G), and *Hmgcoar* (fig. S4H). GO Biological Pathway analysis revealed that, like OxPAPC, both truncated OxPCs and full-length OxPCs induced the “NRF2-mediated oxidative stress pathway”; however, only truncated OxPCs induced the “superpathway of cholesterol biosynthesis” (Fig. 3, D and E, and fig. S4A). Moreover, truncated OxPAPC down-regulated the expression of *Cav1* (caveolin 1), which has been shown to increase hepatic lipid droplet size in NAFLD (*55*–*57*), and up-regulated *Slc25a1*, which has recently been associated with hepatic steatosis and glucose intolerance by dysregulation of hepatocyte metabolism (*58*).

**Fig. 4.**
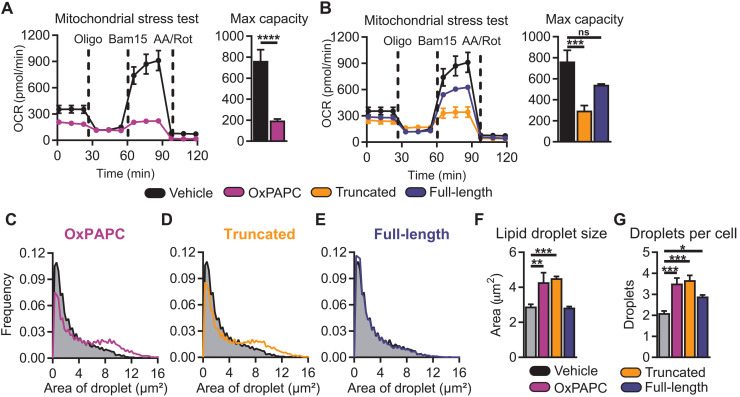
Truncated oxidized phospholipids dysregulate hepatocyte metabolism resulting in lipid droplet accumulation. (**A**) Mitochondrial stress test analysis of AML12 murine hepatocytes treated with OxPAPC (100 μg/ml) for 4 hours significantly inhibited maximum oxygen consumption in hepatocytes (*n* = 5). (**B**) Truncated, but not full-length, OxPLs significantly inhibited maximum mitochondrial oxygen consumption rate (*n* = 5). AML12 cells were treated with (**C**) OxPAPC (100 μg/ml), (**D**) truncated OxPAPC, and (**E**) full-length OxPAPC for 48 hours. (**F**) Lipid droplet size and (**G**) number were significantly increased in AML12 cells treated with OxPAPC and truncated OxPLs. Full-length OxPLs increased droplet number per cell (*n* = 4, three fields of view per biological replicate). Statistical significance was determined by one-way ANOVA and Mann-Whitney *U* test. Multiple comparisons were corrected by FDR or Dunnett’s multiple comparison correction (*P < 0.05, **P < 0.01, ***P < 0.001, and ****P < 0.0001). OCR, oxygen consumption rate.

To investigate the effect of OxPCs on hepatocyte metabolism, we treated AML12 cells with OxPAPC or the fractions enriched for truncated or full-length OxPCs for 4 hours and measured oxygen consumption rate via extracellular flux analysis. Treatment with OxPAPC significantly decreased maximal oxygen consumption rate (Fig. 4A), which was mimicked by truncated OxPCs (Fig. 4B). Impaired oxygen consumption in hepatocytes is indicative of mitochondrial dysfunction that precedes the transition from steatosis to NASH (*9*, *59*). To investigate whether this metabolic dysregulation would lead to increased lipid accumulation, we treated AML12 hepatocytes with OxPAPC, truncated OxPCs, or full-length OxPCs for 48 hours and then stained cells with Nile red to quantify lipid droplet numbers and size (*60*). Consistent with the effects on hepatocyte mitochondrial function, OxPAPC (Fig. 4C) and truncated OxPCs (Fig. 4D) increased average lipid droplet size in cells compared to vehicle, while full-length OxPCs (Fig. 4E) did not alter lipid droplet size (Fig. 4F). However, both fractions increased the number of lipid droplets per cell compared to vehicle control (Fig. 4G).

Together, these data demonstrate that distinct OxPC species differently regulate hepatocyte gene expression and metabolic function in vitro. While both truncated and full-length OxPCs regulate redox transcriptomic programming, only truncated OxPCs regulate anabolic gene programming, such as the superpathway of cholesterol biosynthesis, and inhibit mitochondrial oxygen consumption, resulting in increased lipid droplets.

### scFv-E06 after development of hepatic steatosis halts disease progression to fibrosis

**Fig. 5.**
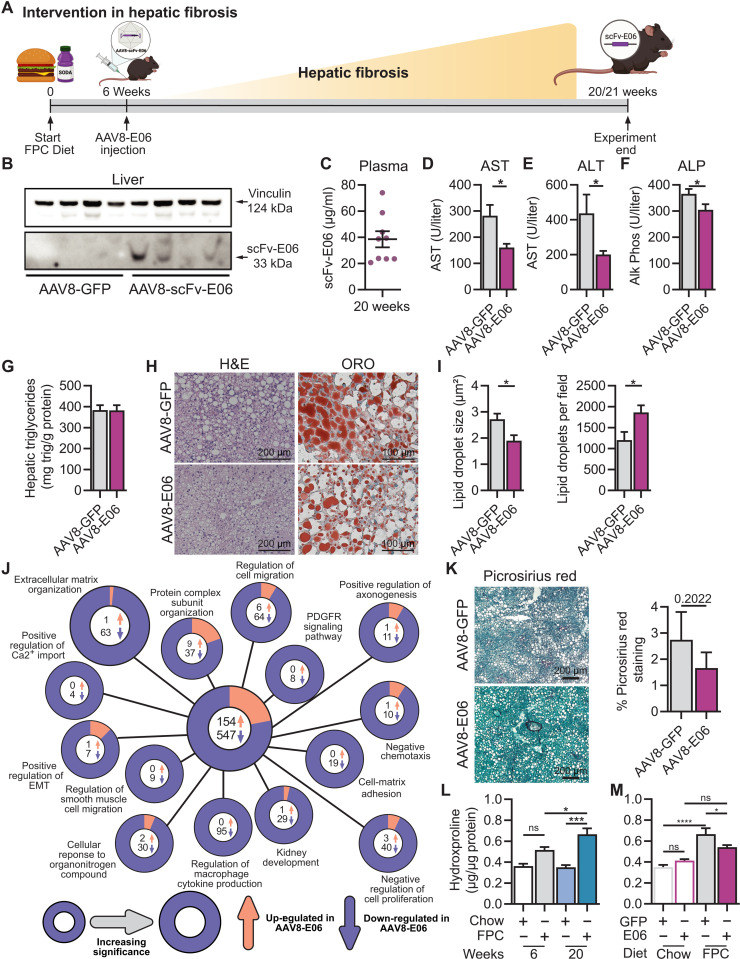
Passive genetic immunization against oxidized phospholipids inhibits liver fibrogenesis. (**A**) Schematic of experimental design. Mice were challenged with FPC diet for 6 weeks. After 6 weeks, mice were injected with AAV8-GFP or AAV8-E06 and fed FPC diet for an additional 14 weeks (total: 20 weeks) (created with BioRender.com). (**B**) Expression of scFv-E06 in mouse liver 14 weeks after injection. (**C**) scFv-E06 titer concentrations were estimated in plasma by competitive sandwich ELISA. (**D**) Plasma AST, (**E**) ALT, and (**F**) ALP levels were decreased in mice expressing scFv-E06 (*n* = 6). (**G** and **H**) H&E and ORO staining revealed no difference in hepatic triglycerides with an (**I**) increased droplet size but reduced number in mice expressing scFv-E06 (GFP, *n* = 6; scFv-E06, *n* = 6; 15 fields of view per biological replicate). (**J**) Pathway analysis of differential gene expression in bulk liver tissue identified 701 genes (154 up-regulated/547 down-regulated) that were significantly regulated by scFv-E06 expression (GFP, *n* = 3; scFv-E06, *n* = 3; fold change > |1.5| and FDR < 0.05). Extracellular matrix organization was the most significantly down-regulated pathway in scFv-E06–expressing mice. (**K**) Representative picrosirius red staining of livers from mice expressing GFP or scFv-E06 after 20 weeks of FPC diet. (**L**) Hydroxyproline concentration was measured in liver tissue from mice fed chow or FPC diet for 6 or 20 weeks. (**M**) Liver hydroxyproline concentration was decreased in mice expressing scFv-E06 after 20 weeks on FPC diet compared to GFP-expressing controls. Quantification of picrosirius red confirmed reduced staining in scFv-E06 mice (*n* = 4). Statistical significance was determined by one-way ANOVA, two-way ANOVA, Spearman’s correlation, and Student’s *t* test with Dunnett’s multiple comparisons correction (*P < 0.05). Statistical outliers were excluded using ROUT test with *Q* = 2%.

To test whether elimination of OxPCs through inducible expression of scFv-E06 during the transition from steatosis to NASH could halt the progression to fibrosis, we first fed age-matched Alb-cre mice an FPC diet for 6 weeks (Fig. 5A), which established hepatic steatosis without signs of fibrosis. Then, weight-randomized mice were injected with AAV8-scFv-E06 or AAV8-GFP via tail vein. To induce fibrosis, mice were fed FPC diet for an additional 14 weeks (Fig. 5A). Sustained scFv-E06 gene expression was confirmed in the liver (fig. S5A), and scFv-E06 protein was detected in the liver by Western blot and in the plasma by ELISA (Fig. 5, B and C) at the end of the experiment (20 weeks). As expected, FPC diet significantly elevated body mass and body fat percentage compared to chow control (fig. S5, B and C); however, there were no differences in body mass (fig. S5B); body fat percentage (fig. S5C); liver, adipose, lung, heart, kidney, and spleen mass (fig. S5, D to I); and fasting glucose and insulin tolerance between AAV8-scFv-E06– or AAV8-GFP–treated mice at 20 weeks (fig. S5, Q to S).

Notably, intervention with AAV8-scFv-E06 protected mice with established hepatic steatosis from further liver injury as evidenced by significantly reduced plasma AST (Fig. 5D), ALT (Fig. 5E), ALP (Fig. 5F), LDL:HDL ratio (fig. S5J), and cholesterol:HDL ratio (fig. S5K), indicating significantly improved liver function compared to control mice. Plasma cholesterol (fig. S5L), LDL (fig. S5M), HDL (fig. S5N), triglycerides (fig. S5O), and albumin (fig. S5P) were not changed by expression of scFv-E06. Total hepatic triglyceride levels were not affected by AAV8-E06 (Fig. 5G); however, histological analysis by H&E and Oil red O staining revealed a decrease in average lipid droplet size and a concomitant increase in lipid droplet number (Fig. 5, H and I) in the livers of mice expressing scFv-E06, a pattern of lipid droplet morphology suggestive of improved liver health (*6*).

To examine whether intervention with AAV8-scFv-E06 affects hepatic gene expression in mice with established steatosis during NASH progression, we performed RNA-seq in bulk liver tissue from mice that were treated with either AAV8-scFv-E06 or AAV8-GFP. Notably, 701 genes (154 up-regulated and 547 down-regulated in scFv-E06–expressing mice) were differentially expressed (fold change > 1.5, FDR < 0.05) between AAV8-scFv-E06– and AAV8-GFP–treated mice. EnrichR and GO Pathway Analysis for Biological Processes revealed that the most significantly down-regulated GO term in the scFv-E06–expressing group was “Extracellular Matrix Organization,” indicating down-regulation of genes associated with matrix production and consequently hepatic fibrosis (Fig. 5J). In addition, “Regulation of Cell Migration,” “PDGFR Signaling Pathway,” “Cell Matrix Adhesion,” and “Regulation of Macrophage Cytokine Production” were all down-regulated, suggesting a reduced fibrotic and inflammatory tone in the liver of mice expressing scFv-E06. Extracellular matrix protein (ECM) gene regulation was confirmed by qRT-PCR. Overall, expression of a panel of ECM proteins was significantly lower in livers of scFv-E06–expressing mice (fig. S6). To assess the extent of hepatic fibrosis in mice expressing scFv-E06, we quantified picrosirius red staining in livers after 20 weeks of FPC diet, which revealed a trend toward a decrease in positive staining in mice that received intervention with scFv-E06 (Fig. 5K). Liver hydroxyproline concentration was significantly increased in GFP-expressing mice fed FPC diet for 20 weeks compared to chow (Fig. 5L), and the FPC-induced increase in hydroxyproline was attenuated by intervention with scFv-E06 after 6 weeks on diet (Fig. 5M). Together, these data demonstrate that intervention with scFv-E06 in mice with established steatosis protects mice from further diet-induced liver damage and hepatic fibrosis.

### Biologically active oxidized phospholipids are reduced by interventional expression of scFv-E06 during the progression to hepatic fibrosis

**Fig. 6.**
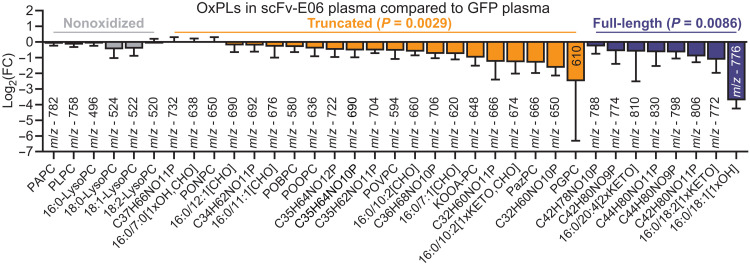
scFv-E06 expression decreases plasma OxPL levels in mice with hepatic fibrosis. OxPL levels were measured in the plasma by LC-MS. Both truncated and full-length OxPLs were significantly reduced by scFv-E06 expression. Nonoxidized phospholipids were unaffected by scFv-E06 expression. Statistical significance was determined by two-way ANOVA. Multiple comparisons were corrected by FDR.

To identify OxPC species that are affected by intervention with AAV8-scFv-E06 in the plasma of mice during the progression to fibrosis, we performed LC-MS as described above, using the in silico–predicted and in vitro–validated compound list (Fig. 2, I and J). We identified 29 OxPC analytes or groups of isobaric compounds and 6 nonoxidized PCs in the plasma of mice with hepatic fibrosis (Fig. 6A). A similar but less pronounced reduction in OxPCs was observed in the liver of scFv-E06 mice compared to GFP controls (sup. Fig. 6B). In addition, there was a slight but statistically significant decrease in nonoxidized PCs in the scFv-E06 liver (fig. S6B). In the plasma, the majority of both truncated and full-length OxPCs were significantly decreased in scFv-E06–expressing mice and included several previously described biologically active compounds containing specific functional groups. Among decreased truncated OxPC species were the carboxylic acid–containing 1-palmitoyl-2-glutaryl-*sn*-glycero-3-phosphocholine (PGPC; *m/z* 610) as well as PazPC (*m/z* 666) (*45*, *46*), a group of γ-keto/hydroxy OxPCs [C32H60NO10P (isobaric group containing HOOA-PC), *m/z* 650]; HODA-PC [1-palmitoyl-2-(9-hydroxy-12-oxo-10E-dodecenoyl)-*sn*-glycero-3-phosphocholine] (*m/z* 706); KOOA-PC (*m/z* 648); C32H60NO11P (isobaric group containing HOdiA-PC) (*m/z* 666) (*22*, *41*), the aldehyde-containing POVPC (*m/z* 594) (*42*), POBPC (*m/z* 580) (*43*), and POOPC (*m/z* 636) (Fig. 6A) (*44*, *45*). Among the most substantially reduced full-length OxPCs were HOME-PC (*m/z* 776) and three isobaric species containing HODE-PC (*m/z* 774), an isobaric group with an *m/z* of 798, containing HETE-PC (*61*), and an isobaric group with an *m/z* of 830, containing isoprostane-PC (Fig. 6A) (*48*, *49*). The predicted structures of the identified compounds with corresponding *m/z* values are shown in table S3.

Selectivity of scFv-E06 for oxidized PCs was further illustrated by the fact that expression of scFv-E06 specifically decreased levels of truncated and full-length OxPC species, while none of the other lipid classes were significantly affected, as demonstrated by comparison of the overall plasma lipidome between scFv-E06– and GFP-expressing mice after either 6 (steatosis) or 20 (fibrosis) weeks of FPC feeding (fig. S7A). Comparing the OxPC profiles at the different stages of disease progression not only demonstrated that individual OxPC species are differentially affected by scFv-E06 but also indicated that levels of OxPCs may be selectively affected during disease progression.

**Fig. 7.**
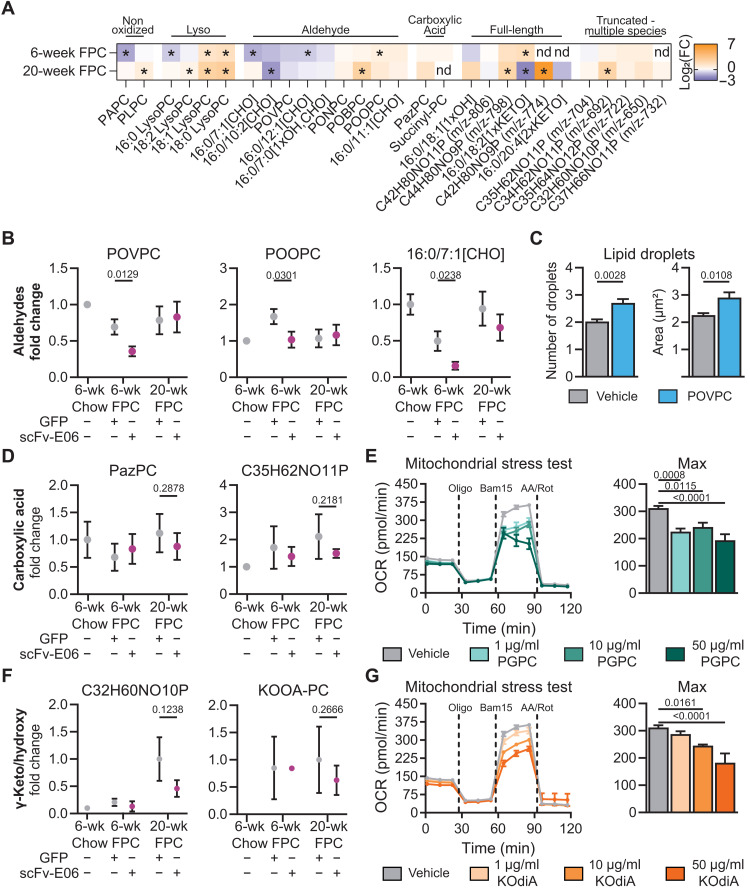
Bioactive oxidized phospholipids are differentially regulated during hepatic steatosis and fibrosis. (**A**) Semiquantitative analysis of OxPL species normalized to their chow counterparts demonstrates a complex, pathology-specific pattern. (**B**) Aldehyde-containing OxPLs, POVPC (6-week Chow GFP, *n* = 3; 6-week FPC GFP, *n* = 7; 6-week FPC scFv-E06, *n* = 6; 20-week FPC GFP, *n* = 10; 20-week FPC scFv-E06, *n* = 10), POOPC (6-week Chow GFP, *n* = 3; 6-week FPC GFP, *n* = 7; 6-week FPC scFv-E06, *n* = 6; 20-week FPC GFP, *n* = 10; 20-week FPC scFv-E06, *n* = 10), and 16:0/7:1[CHO] (6-week Chow GFP, *n* = 3; 6-week FPC GFP, *n* = 5; 6-week FPC scFv-E06, *n* = 5; 20-week FPC GFP, *n* = 7; 20-week FPC scFv-E06, *n* = 8), were decreased by scFv-E06 expression at 6 weeks. (**C**) POVPC, a representative of the aldehyde class, increased lipid droplet size and number after 48 hours in AML12 hepatocytes (*n* = 4). (**D**) Carboxylic acid–containing OxPLs were decreased by scFv-E06 after 20 weeks on diet (6-week Chow GFP, *n* = 3/1; 6-week FPC GFP, *n* = 7/4; 6-week FPC scFv-E06, *n* = 5/2; 20-week FPC GFP, *n* = 9/5; 20-week FPC scFv-E06, *n* = 9/6). (**E**) PGPC, a representative of the carboxylic acid class, decreased maximum oxygen consumption of AML12 hepatocytes after 4 hours (*n* = 8; 50 μg/ml, *n* = 6). (**F**) γ-Keto/hydroxy–containing OxPLs were decreased after 20 weeks on diet (6-week Chow GFP, *n* = 1/0; 6-week FPC GFP, *n* = 5/2; 6-week FPC scFv-E06, *n* = 2/1; 20-week FPC GFP, *n* = 9/2; 20-week FPC scFv-E06, *n* = 8/4), and (**G**) KOdiA-PC, a class representative, decreased maximum oxygen consumption in AML12 hepatocytes in a dose-dependent manner (*n* = 8). Statistical significance was determined by Student’s *t* test.

To study the changes in the levels of the different OxPC classes during the progression from hepatic steatosis to fibrosis, we compared OxPC levels in control mice (expressing AAV8-GFP) that had been fed FPC diet for 6 and 20 weeks to their chow-fed counterparts. Of the nonoxidized species, PAPC and 16:0 LysoPC were decreased after 6, but not 20, weeks on diet, while PLPC and 18:0, 18:1, and 18:2 LysoPC were increased after both 6 and 20 weeks on FPC diet (Fig. 7A). Of the truncated OxPC species, levels of a subset of aldehyde-containing OxPCs, including POVPC, were lower at both 6 and 20 weeks, while carboxylic acid–containing and other aldehyde-containing OxPCs, including POBPC, were increased in response to FPC diet (Fig. 7A).

Full-length OxPC species were differentially regulated at different time points. At 6 weeks, levels of 16:0/18:1[1xOH] trended to be lower compared to chow but higher at 20 weeks. 16:0/18:2[1xKETO] was higher at 6 weeks compared to chow and lower at 20 weeks. Isobaric groups C44H80NO10P and C42H80NO9P, which contain HETE-PCs and HODE-PCs, were significantly higher at 20 weeks. Together, these data show that levels of plasma nonoxidized and individual OxPC species are differently regulated at defined stages of NAFLD progression.

Comparison of OxPCs that were affected by AAV8-scFv-E06 in the plasma of mice at the initiation of hepatic steatosis (Fig. 2K) and during the progression to hepatic fibrosis (Fig. 6A) revealed 28 OxPC species that were decreased in both settings, while 1 OxPC was uniquely identified in hepatic steatosis and 7 OxPC species were uniquely detected in mice with hepatic fibrosis (fig. S7B). Of the seven unique species identified in hepatic fibrosis, four were decreased by more than 50% by scFv-E06. These four species include two previously identified biologically active OxPCs: KOOA-PC and the isobaric group C32H60NO11P containing HOdiA-PC (*41*).

To elucidate at which stage of disease progression these identified OxPC species may exert pathologic activity, we took a closer look at the timeline of scFv-E06–mediated reduction of their plasma levels. The aldehyde-containing species POVPC, POOPC, and 16:0/7:1[CHO] were preferentially reduced by scFv-E06 after 6 weeks on FPC diet (Fig. 7B), implying a role in the initiation of hepatic steatosis. To test whether aldehyde-containing OxPCs affect hepatocyte lipid storage, we treated AML12 hepatocytes with POVPC for 48 hours and assessed lipid droplet size and number per cell. POVPC significantly increased the number and size of lipid droplets (Fig. 7C). On the other hand, carboxylic acid–containing OxPC species (PazPC and C35H62NO11P) were affected by scFv-E06 predominantly after 20 weeks of FPC diet (Fig. 7D), implying a role for these compounds in NAFLD progression to NASH. Treatment of AML12 cells with PGPC (a carboxylic acid–containing OxPC) significantly decreased maximum oxygen consumption rate (Fig. 7E) and increased lipid droplet size and number in a concentration-dependent manner (fig. S8, A and B). Of the γ-keto/hydroxy OxPC species, the levels of KOOA-PC and isobaric group C32H60NO10P (containing HOOA-PC) were reduced by scFv-E06 specifically at 20 weeks of FPC diet (Fig. 7F). Treatment of AML12 hepatocytes for 4 hours with 1-palmitoyl-2-(5-keto-6-octene-dioyl)-*sn*-glycero-3-phosphocholine (KOdiA-PC), a representative γ-keto/hydroxy OxPC, significantly decreased maximum oxygen consumption rate of hepatocytes in a concentration-dependent manner (Fig. 7G). These data show that identified truncated OxPC species that are reduced by scFv-E06 promote a hepatocyte phenotype in vitro that is observed in hepatic steatosis and steatohepatitis.

Hepatic stellate cells are the primary cell niche in the liver that produce fibrotic matrix in response to liver injury (*15*, *62*). To determine whether individual OxPC species that were targeted by scFv-E06 could activate hepatic stellate cells, we challenged LX-2 human hepatic stellate cells with OxPAPC, full-length OxPCs, truncated OxPCs, POVPC, or KOdiA-PC. Treatment of LX-2 hepatic stellate cells for 4 hours with KOdiA-PC, OxPAPC, or POVPC significantly decreased maximum oxygen consumption rate of hepatic stellate cells (fig. S8C).

Previously, *Hmox1* has been implicated in fibrotic activation of hepatic stellate cells (*63*). We found that truncated OxPCs and OxPAPC increased the expression of the NRF2-dependent genes *Hmox1* and *Gclm* in LX-2 human hepatic stellate cells (fig. S8D).

**Fig. 8.**
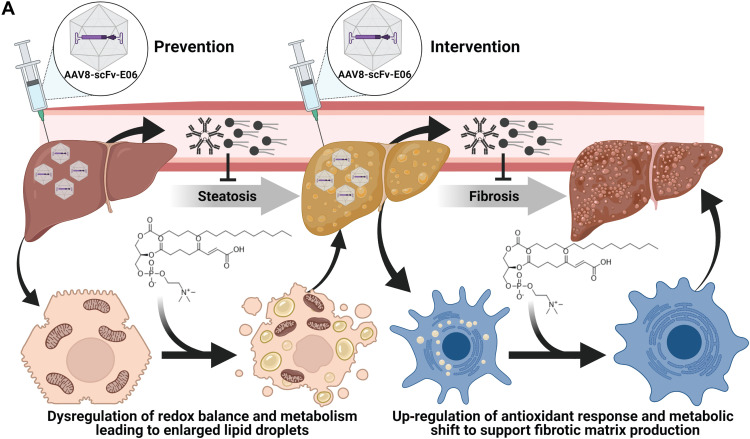
scFv-E06 expression protects mice from hepatic steatosis, and intervention with viral expression of scFv-E06 halts disease progression. (Created with BioRender.com)

Together, these data demonstrate that expression of scFv-E06 decreases levels of individual OxPCs in plasma. FPC diet results in multivariate changes in the OxPC lipidome, which suggest complex regulation of OxPC species in pathology. Identified OxPC species that were decreased by scFv-E06, including aldehyde-, carboxylic acid–, and γ-keto/hydroxy–containing OxPCs, regulate metabolism and gene expression in hepatocytes and hepatic stellate cells, suggesting that they are actively involved in the initiation and progression of hepatic steatosis and fibrosis (Fig. 8A).

## DISCUSSION

Here, we examined a potential therapeutic application of AAV-mediated hepatic expression of the oxidized phospholipid-binding antibody fragment scFv-E06 for the prevention of the initiation of NAFLD and, independently, of the progression to NASH and hepatic fibrosis. We show that AAV8-mediated gene transfer of scFv-E06 is sufficient to express scFv-E06 in a cre-dependent manner in the liver, which by itself leads to secretion of scFv-E06 protein into the plasma. Expression of scFv-E06 in mice fed a chow diet did not alter normal mouse physiology, which provided an excellent tool to interrogate the effect of OxPCs on diet-induced NAFLD initiation and disease progression, without secondary effects on mouse physiology or development, while providing a way to regulate dosing and timing of expression. We demonstrated that hepatic expression of scFv-E06 before the start of FPC diet feeding resulted in a marked reduction in individual OxPC species in plasma, which was sufficient to protect mice from diet-induced hepatic steatosis. Given the efficacy of this model in eliminating plasma OxPCs and subsequent protection from diet-induced hepatic steatosis, we leveraged the flexibility of virus-mediated gene transfer to intervene therapeutically with scFv-E06 expression after the establishment of diet-induced hepatic steatosis. We showed that, in a clinically relevant intervention model, scFv-E06 expression prevented further diet-induced liver damage and hepatic fibrosis independent of obesity and insulin resistance. Separately, scFv-E06 expression was sufficient to reduce a variety of OxPC species in plasma. At present, it is unclear whether reduction of OxPCs is protective in NASH pathogenesis or simply a biomarker of the therapeutic effect of scFv-E06. Using mass spectrometry, we identified individual OxPC species that are decreased by expression of scFv-E06 in mice. Identified OxPC species induced gene expression and metabolic changes in hepatocytes and stellate cells in vitro. These data suggest that plasma OxPCs regulate liver function uniquely at different stages of disease progression. For instance, expression of scFv-E06 before the start of FPC diet protected mice from hepatic steatosis, while intervention with scFv-E06 after established steatosis did not reduce lipid burden in the liver. Despite no overall decrease in lipid burden, scFv-E06–expressing mice had smaller hepatic lipid droplets. This may suggest that OxPCs are involved in dysregulation of cellular lipid storage vital to initiation of hepatic steatosis but not maintenance of steatosis. Together, this suggests that lowering the concentration of identified plasma OxPC species is necessary for preventing hepatic steatosis and progression to fibrosis in a normolipidemic mouse model of diet-induced NAFLD without genetic lipodystrophy. Using this approach, we provide the first evidence that therapeutic intervention after the development of diet-induced steatosis with virus-induced scFv-E06 expression halts progression to hepatic fibrosis in a clinically relevant model of NAFLD.

The natural IgM E06 has been shown to recognize oxidized products of PAPC in vitro and has been used clinically to assess total plasma OxPC levels (*36*, *64*). However, individual OxPC species that are eliminated by E06 in vivo had not been identified. Our findings demonstrate that scFv-E06 recognizes a variety of OxPC species irrespective of the type of oxidative modification or *sn*-1 position acyl-chain length while not affecting plasma levels of nonoxidized lipids in vivo. Further research is necessary to investigate binding affinities and specificity of scFv-E06 among OxPC species and to demonstrate the potency of scFv-E06 as a potential therapeutic in the context of NAFLD and other oxidative stress–induced diseases.

Oxidized phospholipids have previously been shown to regulate numerous cellular functions and biological processes (*20*, *21*, *27*, *29*, *30*, *34*, *35*, *38*, *65*–*67*). We show that individual OxPCs that are targeted by scFv-E06 activate hepatocytes, resulting in up-regulation of the evolutionarily conserved NRF2-dependent antioxidant program and metabolic dysregulation. While both truncated and full-length OxPCs up-regulated genes related to oxidative stress, only truncated OxPCs up-regulated prosteatotic anabolic pathways including cholesterol biosynthesis. Induction of de novo cholesterol synthesis leading to cholesterol accumulation has been shown to promote transition from hepatic steatosis to NASH (*68*) and may be one mechanism through which OxPCs promote disease progression. Furthermore, identified OxPCs inhibited mitochondrial oxygen consumption in hepatocytes, consistent with a switch to an anabolic cellular phenotype, and resulted in increased lipid droplet formation, a hallmark of hepatic steatosis (*6*). Together, our findings demonstrate that hepatocytes recognize and respond to OxPCs, and treatment of hepatocytes in vitro with truncated OxPCs phenocopies pathological alterations in mitochondrial bioenergetics and lipid droplet regulation seen during the development of hepatic steatosis. Differences in regulation of the transcriptome in mice expressing scFv-E06 compared to GFP during development of diet-induced hepatic steatosis may give key insights into the mechanisms by which OxPCs drive early metabolic changes in hepatocytes that promote disease initiation.

Levels of truncated OxPCs containing a γ-keto/hydroxy functional group were also reduced by scFv-E06 in vivo. Several biological functions have been described for these lipids (*22*, *41*); however, it is unknown if γ-keto/hydroxy–containing OxPCs play a role in NASH and hepatic fibrosis. Here, we show that KOdiA-PC inhibits mitochondrial oxygen consumption and extracellular acidification rate in hepatic stellate cells. In addition, truncated OxPCs induced expression of the NRF2-dependent genes *Hmox1* and *Gclm* in hepatic stellate cells. These data demonstrate that individual truncated OxPCs that are targeted by scFv-E06 regulate hepatic stellate cell bioenergetics and gene expression and may polarize the cells toward a redox-regulatory state.

Plasma levels of both truncated and full-length OxPC species were significantly reduced by scFv-E06 either at the initiation of hepatic steatosis or during the progression to hepatic fibrosis. These data demonstrate that scFv-E06 specifically recognizes individual oxidized phosphorylcholine phospholipids in vivo, without affecting levels of OxPCs or lyso-PCs, which builds on previous studies that have demonstrated similar specificity of E06 in vitro (*37*, *69*). We identified one unique OxPC species in the setting of hepatic steatosis and seven unique OxPC species in the setting of hepatic fibrosis that are targeted by scFv-E06. Identification of specific OxPC species that are eliminated by scFv-E06 during the different stages of disease progression suggests that plasma OxPCs could serve as noninvasive biomarkers of NAFLD severity. In human plasma, oxidized phospholipids are carried on LDLs and previous work has shown that the ratio of OxPCs to apoB-100 or apo(a) correlates with cardiovascular disease, calcific aortic valve disease, and aortic valve stenosis (*70*, *71*). These risk factors are assessed from lipoprotein-bound OxPCs. Our method assesses free or “unconjugated” OxPCs. Considering the pathologic role of OxPCs in NAFLD and the dearth of viable noninvasive biomarkers for NASH, analysis of individual unconjugated OxPC species may provide an additional diagnostic metric and possible alternative to more invasive techniques like liver biopsy.

In this study, we limited our analysis to validated OxPC species predicted by LPPTiger (*40*) and identified in air- and copper-oxidized PAPC and PLPC. Combining an in silico–based approach with in vitro validation allowed us to assess 83 validated analytes (individual lipid species and isobaric groups); however, this accounts for only a fraction of the possible oxidation species present in vivo. Given the numerous possible oxidative modifications that can arise as products of oxidation, many oxidized phospholipid species are isobaric requiring additional metrics to accurately distinguish individual species. In this study, we leverage both mass and chromatographic retention time to distinguish between OxPC species. Consequently, isobaric species with similar retention times were not uniquely identified. Future studies are necessary to identify unique fragmentation patterns for isobaric species that overlap chromatographically to confidently identify each species. In addition, expanding the OxPL panel to include not only other OxPC species but also phospholipids with other head groups, which have been shown to play critical roles in diverse biological processes ranging from thrombus formation (*72*) to ferroptosis (*31*) and apoptosis (*73*), is essential to understand how OxPLs regulate complex pathologies. Currently, our method is limited to semiquantitation of OxPL species because of the lack of deuterated and nondeuterated OxPL standards. Synthesis of OxPL standards is necessary to directly quantify the concentration of pathology-driving OxPLs in the plasma. In addition, our method measures OxPCs that can be isolated by liquid-liquid organic extraction, which likely reflects the free OxPC lipidome as it is likely that some electrophilic lipid species bind covalently with proteins. For example, POVPC has previously been shown to bind manganese superoxide dismutase (*35*).

We demonstrate that scFv-E06 expression lowers levels of OxPCs in the plasma, which are necessary for pathogenesis of both hepatic steatosis and fibrosis. Furthermore, although OxPC levels are lowered by scFv-E06 expression as measured by mass spectrometry, the mechanism through which this occurs remains unclear. We propose two possible mechanisms that will require further study: (i) elimination from the biological system by enzymatic activity or excretion or (ii) masking of OxPC inhibiting their biological effects. Distinguishing whether it is necessary to lower both plasma and liver OxPC species to inhibit disease progression as well as understanding the mechanism through which OxPC species are lowered by scFv-E06 expression will provide a deeper understanding of the role of OxPC species in NAFLD pathology and guide future therapeutic approaches.

In conclusion, our study establishes the translational potential of plasma OxPC elimination using AAV8-mediated gene transfer of scFv-E06 in a clinically relevant NAFLD model. This approach to target plasma OxPCs provides multimodal control over oxidized lipid-driven pathologies. Temporal and spatial regulation of scFv-E06 expression will allow for exploration of previously inaccessible OxPC-mediated biology and pathology. Last, assessment of plasma levels of individually identified, pathology-driving OxPC species during NAFLD initiation and progression may lead to the discovery of urgently needed noninvasive biomarkers.

## MATERIALS AND METHODS

### Study design

The objective of this study was to determine whether AAV8-induced expression of scFv-E06 was sufficient to prevent hepatic steatosis by decreasing plasma OxPCs, and further to intervene in progression from hepatic steatosis to hepatic fibrosis by decreasing plasma OxPCs. Group size was selected on the basis of similar studies previously reported in the literature. The number of mice included in all groups was selected before the start of study and not altered throughout the study. End points to assess hepatic steatosis (6 weeks on diet) and hepatic fibrosis (20 weeks on FPC diet) were defined before the start of the study. Mice were randomized into each group based on body mass and excluded from analysis if they did not gain weight in response to FPC diet feeding. The study was blinded for the duration of FPC diet feeding and after for all subjective analyses. All animal experiments were approved by the University of Virginia’s Animal Care and Use Committee (protocol #3444). For each analysis, statistical outliers were assessed using the robust regression and outlier removal method (ROUT) method (*Q* = 5%). Outliers were excluded from final analysis and determination of statistical significance. All in vitro experiments were conducted, at minimum, in duplicate, and representative results were reported.

### Generation of AAV8-scFV-E06

We developed a cre-dependent adeno-associated viral construct for expression of scFv-E06. The E06 coding region was synthesized by GenScript from the publicly available, published sequence (Piscataway, NJ, USA), containing a C-terminal myc- and His-tag and flanking 5′ Mlu I and 3′ Nhe I restriction sites. This DNA fragment was then cloned into the unique Nhe I–Asc I restriction sites of pAAV-EF1a-double floxed-hChR2(H134R)-EYFP-WPRE-HGHpA (gift from K. Deisseroth—Addgene, plasmid #20298). The subsequent construct contained the scFv-E06 fusion in the inverse orientation from the EF1a promoter. Cre recombinase expression then reverses the orientation of scFv-E06 and allows expression. AAV viral particles using serotype 8 were then prepared by the University of Pennsylvania Vector Core Facility.

### Mice

B6.Cg-Speer6-ps1^Tg(Alb-cre)21Mgn^/J (The Jackson Laboratory, 003574) (Alb-cre) were obtained from The Jackson Laboratory (Bar Harbor, ME) and housed in the Pinn vivarium at the University of Virginia Center for Comparative Medicine. Unless otherwise stated, animals were maintained in pathogen-free housing with a 12-hour light/dark cycle with ad libitum access to food and water. All animal experiments were approved by the University of Virginia’s Animal Care and Use Committee (protocol #3444). To assess the impact of scFv-E06 on hepatic steatosis, 6-week-old mice were injected with either AAV8-GFP (UNC Vector Core) or AAV8-scFv-E06 [10^11^ genome copies/100 μl of sterile phosphate-buffered saline (PBS)]. Mice, 8 weeks of age, were fed a high FPC diet (Teklad TD.190142) supplemented with 4.2% glucose (Thermo Fisher Scientific, D12-500)/fructose (Thermo Fisher Scientific, L95-500) water (55%/45%, w/w) for 6 weeks. At the end of the experiment, mice were euthanized via CO_2_ inhalation and cardiac puncture. To assess the impact of scFv-E06 expression on fibrosis and whether expression of scFv-E06 after the onset of hepatic steatosis could mitigate progression to hepatic fibrosis, mice, 6 weeks of age, were fed FPC diet for 6 weeks. After 6 weeks of feeding, mice were injected via tail vein with AAV8-GFP or AAV8-scFv-E06. Mice continued FPC diet for an additional 14 weeks, at which point they were euthanized via CO_2_ inhalation. Control mice were fed chow diet (Teklad 7912).

### Histology

PBS-perfused liver tissue was either fixed in 10% formalin (w/v) for 24 hours and transferred to 200 proof ethanol or cryopreserved in NEG-50 (Richard Allan Scientific, 6502) and stored at −80°C. Tissue samples were processed for histology at the Robert M. Berne Cardiovascular Research Center Histology Facility at the University of Virginia. Briefly, samples were embedded in paraffin, and 10-μm serial sections were stained with H&E, picrosirius red, or Oil red O. Images were collected using Olympus BX51 or Leica Thunder with a total internal reflection fluorescence microscope operating in bright field. Picrosirius red counterstained with Fast Green was quantified using ImageJ (*74*) to assess stained area in three sections from the liver tissue of each mouse. Positive staining was determined by thresholding while blinded after exclusion of perivascular collagen: Threshold settings were kept constant for all samples assessed.

### Triglyceride assay

Hepatic triglyceride concentration was quantified using triglyceride colorimetric assay (Pointe Scientific, T7532500). Liver tissue (20 to 30 mg) was lysed in 400 μl of 0.6% NaCl using Qiagen TissueLyser II (30 Hz for 15 min). Samples were diluted 1:4 in 0.6% NaCl and mixed with 4.4 parts chloroform (EMD UN1888/Fisher Scientific, C607-1) and 2.2 parts methanol (Sigma-Aldrich, 646377-1 L). Samples were vortexed vigorously and centrifuged for 10 min at 3000*g* to accelerate phase separation. The organic phase (500 μl) of each sample was transferred to a new tube and dried under N_2_ purge. The dried organic phase was resuspended in 200 μl of 95% ethanol (200 μl) (Fisher Scientific, 04-0355-223). Samples from mice on FPC diet were diluted 1:5 in 95% ethanol. Assay was conducted in triplicate according to the manufacturer’s recommendations.

### Plasma liver biomarkers

Whole blood was collected from mice after euthanasia via cardiac puncture and dispensed into heparin-coated plasma collection tubes (Becton Dickinson, 365985) and stored on ice. Plasma was separated via centrifugation at 2000*g* for 15 min at 4°C. Plasma was stored at −80°C. Plasma was diluted 1:1 in sterile 0.9% saline, and liver biomarkers were analyzed by UVA Clinical Laboratories.

### Cell culture

AML12 murine hepatocytes (American Type Culture Collection, CRL-2254) were cultured in Dulbecco’s modified Eagle’s medium (DMEM):F12 supplemented with 10% fetal bovine serum (FBS) (R&D Systems, S12450/Atlanta Biologicals, S11150) and 1% Anti-Anti (Gibco, 15240-062) and grown at 37°C with 5% CO_2_. Cells were passaged when they reached 90% confluency using 0.5% trypsin (Gibco, 15400-054). LX-2 human hepatic stellate cells (MilliporeSigma, SCC064) were cultured in DMEM supplemented with 2% FBS (R&D Systems,S12450/Atlanta Biologicals, S11150) and 1% penicillin-streptomycin (Gibco, 15140-122) and grown in DMEM (Gibco, 11965-092) at 37°C with 5% CO_2_. Cells were passaged when they reached 90% confluency using 0.25% trypsin (Gibco, 25200-056). For all experiments using the LX-2 cell line, cells were cultured on Matrigel (83 μg/ml) (Fisher Scientific, CB-40230) for 24 hours in DMEM containing 1% penicillin-streptomycin before the start of the experiment.

### Mitochondrial and glycolytic stress test

#### XF24 seahorse bioenergetics assay

AML12 hepatocytes (75,000 cells per well) were plated in complete medium in XF24 cell culture microplates (Agilent, 100777-004) and allowed to settle overnight. The following day, cells were treated for 4 hours with oxidized phospholipids (10 to 100 μg/ml) in DMEM:F12 supplemented as described. At the end of the experimental treatment, the medium was removed and replaced with assay appropriate medium: mitochondrial stress test medium (Corning, 50-003-PB). Oxygen consumption was measured via mitochondrial stress test. The rate of *P*O_2_ (partial pressure of oxygen) consumption was measured every 10 min for a 4-min interval preceded by a 3-min mixing and 3-min waiting interval. Oligomycin A (Sigma-Aldrich, 75351) (0.91 μM), BAM15 (Cayman Chemical Company 17811) (1.667 μM), antimycin A (Sigma-Aldrich, A8674) (7.692 μM), and rotenone (Sigma-Aldrich, R88751G) (7.692 μM) were used to interrogate basal, reserve, and maximum oxygen consumption capacity.

#### XFe96 seahorse bioenergetics assay

AML12 hepatocytes were plated (25,000 cells per well) in complete medium in XFe96 cell culture microplates (Agilent, 101085-004) and incubated for 1 hour at room temperature before settling overnight at 37°C. The following day, cells were treated for 4 hours with oxidized phospholipids (10 to 100 μg/ml) in DMEM:F12 supplemented as previously described. After treatment, the medium was removed and replaced with assay appropriate medium: mitochondrial stress test medium (Corning, 50-003-PB) and glycolytic stress test medium (Sigma-Aldrich, D5030) supplemented with 143 mM NaCl (Thermo Fisher Scientific, S671-3) and 2 mM l-glutamine (Gibco, 25030-081). Oxygen consumption was measured via mitochondrial stress test. The rate of *P*O_2_ consumption was measured every 10 min for a 4-min interval for 30 min before sequential challenge with (i) oligomycin A (Sigma-Aldrich 75351) (1 μM), (ii) BAM15 (Cayman Chemical Company, 17811) (2 μM), and (iii) antimycin A (Sigma-Aldrich, A8674) (10 μM) and rotenone (Sigma-Aldrich, R88751G) (10 μM). *P*O_2_ consumption was measured as described previously to analyze basal, reserve, and maximum oxygen consumption capacity. Glycolytic rate was measured via extracellular acidification rate. The rate of pH change was measured every 10 min for a 4-min interval for 30 min before sequential challenge with (i) glucose (Sigma-Aldrich, D9434) (20 mM), (ii) oligomycin A (Sigma-Aldrich, 75351) (1 μM), and (iii) 2-deoxyglucose (Sigma-Aldrich, D8375) (80 mM) to interrogate basal, reserve, and stressed glycolytic rate.

### Quantitative real-time PCR

#### In vitro

RNA was isolated from cells lysed in RLT lysis buffer using the RNeasy Mini Kit (Qiagen 74106). The manufacturer’s recommendations were followed for RNA isolation. RNA quantity and purity were analyzed by spectrometric analysis. cDNA was synthesized from 250 ng of total RNA using an iScript cDNA synthesis kit (Bio-Rad, 1708891) according to the manufacturer’s recommendations. SensiMIX SYBR Green (Bioline, QT615-05) was used to quantify gene expression. Relative gene expression was calculated using the ΔΔCq method normalized to cyclophilin A in AML12 cells and *Hprt* in LX-2 cells.

#### Ex vivo

Liver tissue was stored at −80°C in RNAlater (Sigma-Aldrich, R0901) until analysis. Liver tissue was lysed using Qiagen TissueLyser II (30 Hz for 15 min) in RLT lysis buffer. The manufacturer’s recommendations were followed for RNA isolation using the RNeasy Mini Kit. RNA quantity and purity were analyzed by spectrometric analysis (260/280, >1.8 and <2.2; 260/230, >1.8 and <2.2). cDNA was synthesized from 250 ng of total RNA using an iScript cDNA synthesis kit according to the manufacturer’s recommendations. SensiMIX SYBR Green was used to quantify gene expression. Relative gene expression was calculated using the ΔΔCq method normalized to β-2-microglobulin. Primer sequences were generated using NCBI Primer Blast and span an exon-exon junction to ensure mRNA specificity and synthesized by Eurofins or Integrated DNA Technologies (table S4).

### Air oxidation of PAPC

One milligram of PAPC (Avanti Polar Lipids, 850459C) was dried in a 13 × 100 borosilicate glass tube (Corning, 99445-13) with nitrogen, covered loosely with aluminum foil to allow gas exchange, and oxidized by exposure to air for 7 to 12 days to generate the oxidized phospholipid mixture, OxPAPC. Level of oxidation was monitored by LC-MS to maintain a consistent oxidation profile.

### Copper oxidation of PLPC

Ten micrograms of PLPC (Avanti Polar Lipids, 850458) was transferred to a tube and dried under N_2_. PLPC was resuspended in water containing CuCl (10.547 mM) and H_2_O_2_ (659 mM) and oxidized for 19 hours at 37°C to generate oxidized PLPC (OxPLPC). OxPLPC was extracted via liquid-liquid extraction using chloroform:methanol:water (1:1:1). Briefly, chloroform, methanol, and water were added to a glass tube. The extraction mixture was vortex vigorously and centrifuged at 805*g* for 10 min at 20°C. After centrifugation, the organic layer was transferred to a new a tube and dried under nitrogen. OxPLPC was resuspended in methanol.

### Oxidized phospholipid quantification by LC-MS/MS

#### OxPL extraction from plasma

Plasma was collected from mice as described above and stored at −80°C in 50 μM butylated hydroxytoluene to prevent ex vivo oxidation. Twenty-five microliters of plasma was added to 1.975 ml of high-performance LC (HPLC) water (Tedia, WS2211-001) in a 13 × 100 borosilicate glass tube (Corning, 99445-13). Two milliliters of chloroform (EMD, UN1888/Fisher Scientific, C607-1) and 2 ml of methanol (Sigma-Aldrich, 646377-1L) containing 16.24 nM 1-palmitoyl-2-glutaryl-*sn*-glycerol-3-phosphocholine-*d*6 (Cayman Chemical, 25746) were added and vortexed vigorously. Samples were centrifuged at 805*g* (20°C) for 10 min to accelerate phase separation. The organic phase was transferred to a new tube, and 2 ml of chloroform was added to the original tube. The extraction was repeated twice more for a total of three times. Samples were dried under nitrogen and resuspended in 200 μl of methanol and vortexed vigorously. Samples were transferred to sample vials for LC-MS analysis.

#### OxPL extraction from liver

Liver tissue was collected from mice and stored at −80°C in 50 μM in butylated hydroxytoluene to prevent ex vivo oxidation. Liver tissue was homogenized using Qiagen TissueLyser II (30 Hz for 15 min) in water. Thirty-five milligrams of tissue was used for extraction as described previously (see the previous section). Samples were resuspended in 200 μl of HPLC butanol (PHARMCO-AAPER 13050-03).

#### In silico phospholipid oxidation and method development

LPPTiger (*40*) was used to predict possible oxidation products of PAPC and PLPC. Oxidation level was set to level 3. Maximum modification site was set to 8, max keto was set to 8, max peroxy was set to 3, and max epoxy was set to 3. Nine hundred eighty oxidation species were predicted for PAPC, and 67 oxidation species were predicted for PLPC. Isobaric species were combined as a single analyte recording corresponding to the chemical formula. After combining isobaric species, there were 180 potential analytes for PAPC and 35 potential analytes for PLPC. Predicted analytes were validated by mass [<5-ppm (parts per million) variance from predicted mass] using PAPC oxidized by air and PLPC oxidized by copper (I) chloride.

#### Lipid analysis

Oxidized phospholipids were measured using Thermo Fisher Scientific Q Exactive coupled with a Vanquish UHPLC. Samples were separated by reversed-phase chromatography using C18 Phenomenex 4.6 μm × 100 mm with 69% methanol–31% water with 10 mM ammonium acetate (mobile phase A) and 50% methanol–50% isopropanol with 10 mM ammonium acetate (mobile phase B) at a flow rate of 0.5 ml/min using the following gradient: 0 to 4 min 0% B, 4 to 6 min 0 to 17.5% B, 6 to 12 min 17.5% B, 12 to 14 min 17.5 to 25% B, 14 to 21 min 25% B, 21 to 24 min 25 to 60% B, 24 to 33 min 60% B, 33 to 36 min 60 to 65% B, 36 to 40 min 65% B, 40 to 43 min 65 to 0% B, and 43 to 50 min 0% B. Q Exactive was operated in positive mode using parallel reaction monitoring mode with an inclusion list and the following settings: MS^2^ resolution, 17,500; automatic gain control (AGC) target, 1 × 10^5^; maximum injection time (IT), 100 ms; isolation window, 1.0 *m/z*; normalized collision energy, 27. Analyte detection was limited to inclusion list within a specified retention window determined from in vitro OxPAPC and PLPC. Peaks corresponding to individual oxidized phospholipid species or isobaric groups were identified using Xcalibur (v4.1) QuanBrowser based on mass (<5-ppm variance from predicted mass) and validated retentions times. Peak areas were normalized to PGPC-*d*6. Biological replicates were excluded from analysis for an individual analyte if the analyte was not detected.

### Lipidomics LC-MS and data analysis

The plasma lipidome was assessed using Thermo Fisher Scientific Q Exactive coupled with a Vanquish UHPLC. Samples were separated by reversed-phase chromatography using Thermo Scientific Acclaim 120 (C18 5 μm 120 Å 4.6 × 100 mm) with 50% acetonitrile, 50% water, and 0.1% formic acid with 10 mM ammonium formate (mobile phase A) and 88% isopropanol, 10% acetonitrile, 2% water, and 0.02% formic acid with 2 mM ammonium formate (mobile phase B) at a flow rate of 400 μl/min using the following gradient: 0 to 4 min 30 to 60% B, 4 to 10 min 60 to 80% B, 10 to 15 min 80 to 90% B, 15 to 24 min 90 to 100% B, 24 to 27 min 100% B, 27 to 27.1 min 100 to 30% B, 27.1 to 31 30% B. Q Exactive was operated in positive mode and collected spectra using full MS data-dependent MS^2^ mode with an inclusion list containing analytes in Splash Lipidomix Mass Spec Standard (Avanti, 330707) using the following settings: full MS settings: resolution, 35,000; AGC target, 1 × 10^5^, max IT, 128 ms; scan range, 200 to 1500 *m/z*; dd-MS^2^ settings: resolution, 17,500; AGC target, 2 × 10^5^; max IT, 64 ms, loop count, 5; normalized collision energy (NCE), 40. Data were analyzed using LipidSearch (version 4.1.16) with the following settings: search—database: Q Exactive, precursor tolerance, 5.0 ppm; product tolerance, 8.0 ppm; alignment—alignment method, mean; retention time tolerance, 0.25 min. Samples were normalized to the internal standard PGPC-*d*6 to control for extraction efficiency.

### In vitro quantification of lipid droplet

AML12 cells were fixed with 4% paraformaldehyde (diluted from 16%, Alfa Aesar, 43368-9M) and stained with Hoechst blue (Invitrogen, 953557) and Nile red (Invitrogen, N1142). Two images (Zeiss Axiovert 200 with QICAM Fast 1394), one of 4′,6-diamidino-2-phenylindole (DAPI) and one of Nile red, were taken at three locations in each well. Lipid droplet size and quantity were calculated from epifluorescent widefield micrographs using an ImageJ plugin, MRI Lipid Droplets (*75*). Lipid droplets were identified as areas larger than five pixels. MRI Lipid Droplets ImageJ plugin was used to identify nuclei in DAPI staining and count total cell number.

### In vitro and ex vivo RNA-seq

#### In vitro

AML12 hepatocytes (100,000 cells per well) were treated with OxPAPC (100 μg/ml), truncated OxPAPC (100 μg/m), and full-length OxPAPC (100 μg/ml) for 4 hours. RNA was isolated using the RNeasy Mini Kit (Qiagen 74106). RNA quantity and purity were analyzed by spectrometric analysis (260/280, >1.8 and <2.2; 260/230, >1.8 and <2.2; RNA integrity number ≥ 8). cDNA libraries were generated using the NEBNext Ultra II Directional RNA Library Prep Kit (New England Biosciences, E7760S). cDNA library fragment size was verified using Bioanalyzer 2100. Samples were sequenced by the UVA Genomics and Technology core with a read length of 75 base pairs (bp) and a target depth of 10 million reads using the Illumina NextSeq 500 Sequencing System.

#### Ex vivo

After dissection, liver tissue was stored at −80°C in RNAlater (Sigma-Aldrich, R0901) until use. Liver tissue was lysed using Qiagen TissueLyser II (30 Hz for 15 min) in RLT lysis buffer (Qiagen, 1015762). The manufacturer’s recommendations were followed for RNA isolation using the RNeasy Mini Kit (Qiagen, 74106). RNA quantity and purity were analyzed by spectrometric analysis (260/280, >1.8 and <2.2; 260/230, >1.8 and <2.2). RNA was shipped to GeneWiz (South Plainfield, NJ). RNA was sequenced in a strand-specific manner with a read length of 150 bp and a target depth of 20 million to 30 million reads.

#### Data analysis

Reads were aligned using UVA Rivanna Supercluster using Spliced Transcripts Alignment to a Reference (STAR) (*76*). Reads were trimmed and aligned to the mouse reference genome (mm10) with either single- or pair-end alignment where appropriate. Aligned reads were counted, and differential gene expression was calculated if reads exceeded 1 read per million using EdgeR (*77*) and RStudio. Genes were considered differentially expressed if they deviated from the control condition by 50% with a *P* value less than 0.05 (in vitro) or 0.1 (in vivo). EnrichR (*53*) was used to identify pathways that were up- or down-regulated on the basis of differentially regulated genes.

### Magnetic resonance imaging

Body composition analysis was performed by EchoMRI-100H on mice before the start of FPC diet feeding and weekly for the duration of feeding.

### Western blot

#### Tissue

Tissues were lysed using Qiagen TissueLyser II (30 Hz for 15 min) in radioimmunoprecipitation assay (RIPA) lysis buffer containing cOmplete Mini protease inhibitors (Roche, 37439120) and phosphatase inhibitors (Sigma-Aldrich, P5726 and P0044). Protein concentration was quantified via Pierce BCA Protein Assay (Thermo Fisher Scientific, 23225). Total protein (25 to 75 μg) was separated by SDS–polyacrylamide gel electrophoresis (PAGE) (8 to 12%) and transferred to nitrocellulose or polyvinylidene difluoride membranes. Membranes were blocked with Intercept Blocking Buffer (LI-COR Biosciences, 927-70001) or 5% bovine serum albumin in tris-buffered saline (TBS) or 5% milk powder in TBS with 0.1% Tween 20 for 1 hour at room temperature. Membranes were stained with goat anti-myc [horseradish peroxidase (HRP)] antibody (1:30,000; NovusBio, NB600-341) or rabbit anti-vinculin antibody (1:1000; CST, E1E9V) diluted in 1% milk powder in TBS with 0.1% Tween 20 overnight at 4°C. Next, membranes were washed in TBS containing 0.1% Tween 20, followed by incubation for 1 hour at room temperature with HRP-conjugated secondary antibodies (1:10,000 dilution in 1% milk in TBS with 0.1% Tween 20, CST), except for membranes incubated with the goat anti-myc [HRP] antibody. Membranes were washed in TBS containing 0.1% Tween 20 and imaged on the Odyssey Imager (LI-COR Biosciences) or incubated with ECL substrate (Thermo Scientific SuperSignal, 34580) for 5 min at room temperature and imaged using Amersham ImageQuant 800.

#### Plasma

Plasma was diluted in loading dye (150 μl/ml) and denatured at 95°C for 10 min. Heat-denatured samples were separated by SDS-PAGE gel and transferred to nitrocellulose membranes. Membranes were blocked with PBS Blocking Buffer (LI-COR Biosciences) and for 1 hour at room temperature and stained with a 1:1000 dilution of mouse anti-myc antibody (Millipore, 05-724) overnight at 4°C. The blot was washed three times for 15 min with PBS Blocking Buffer. The blot was stained with the secondary antibody IRDye 800CW Goat anti-Rabbit (1:10,000 in PBS) (LI-COR Biosciences, 926-32211) for 1 hour at room temperature followed by three washes with LI-COR PBS Blocking Buffer. The resulting blot was visualized using the Odyssey Imager (LI-COR Biosciences).

### HIS/myc competitive sandwich ELISA

Ninety-six–well Nunc MaxiSorp flat-bottom plates (Thermo Fisher Scientific) were coated with rabbit anti HIS-Tag antibody (1:250 in 1× PBS; CST, 2365S) overnight at 4°C. Coated wells were then aspirated and washed three times with 1× PBS (~1-min soaking in between). After blocking for 1 hour at room temperature with 1× ELISA/Elispot Diluent (Invitrogen, 19045636) diluted in double-distilled water, wells were washed as previously described. Plasma samples were diluted 1:20 in double-distilled water and added into the wells. For the HIS-competitive standard curve, 2 μg of HIS-protein ELISA standard (stock, 50 μg/ml; Cayman Chemical, 0556338) was serially diluted in double-distilled water and E06 plasma sample was added (1:20). The E06 plasma standard curve was generated by serial dilution of plasma. A HIS-protein competitive standard curve was generated by serial dilution of HIS-tagged 4EBP1 (11.1 mg/ml). Blanks were incubated with 1× ELISA/Elispot Diluent. Plates were sealed and incubated overnight at 4°C. Following five washing steps, the detection antibody goat anti–c-Myc HRP-coupled (1:5000 in 1× ELISA/Elispot Diluent; Novus P26) was added to the plates and incubated for 1 hour at room temperature. After washing seven times, 1× TMB ELISA Substrate Solution (eBioscience Inc., E00008-1655) was pipetted into the wells and incubated for 35 min. Absorbance was measured using Plate Reader (BioTek) at 450 and 570 nm.

### Glucose and insulin tolerance tests

Intraperitoneal glucose (GTT) and insulin tolerance tests (ITT) were performed on fasted mice at time points indicated. Mice were fasted for 6 hours, and body mass and basal blood glucose were measured via tail vein nick using a glucometer (CVS Health). For GTT, glucose (1 mg/g) in sterile water was administered by intraperitoneal injection, and blood glucose was recorded 15, 30, 45, 60, 90, and 120 min after injection. For ITT, Humulin R (100 U/ml, Lilly) was diluted in sterile 0.9% saline, 0.75 U/kg was administered by intraperitoneal injection, and blood glucose was recorded 15, 30, 45, 60, 90, and 120 min after injection.

### Cholesterol assay

Hepatic cholesterol was assessed using the Amplex Red Cholesterol Assay Kit (Invitrogen, A12216). Liver tissue was weighed and lysed in 400 μl of RIPA lysis buffer (EMD Millipore 20-188) using TissueLyser II. The protein concentration of the lysate was determined by Pierce BCA Protein Assay (Thermo Fisher Scientific, 23225). Lysates were diluted (15 μg per reaction) and analyzed in triplicate for free, esterified, and total cholesterol according to the manufacturer’s recommendations. Final hepatic cholesterol concentrations were reported as micrograms of cholesterol per milligram of liver protein.

### Hydroxyproline assay

Liver tissue (20 to 30 mg) was lysed in 200 μl of HPLC water (Sigma-Aldrich, 270733) using TissueLyser II shaking at 30 Hz for 15 min. Lysates were centrifuged at 20,817*g* at 4°C for 15 min. Supernatants were collected, and protein concentration was assessed by Pierce BCA Protein Assay (Thermo Fisher Scientific, 23225). Five hundred micrograms of protein was diluted 1:1 in 37% HCl (12.1 M) to a final concentration of 1.89 mg/ml in ~6 M HCl. The lysates were incubated at 95°C for 20 hours. After 20 hours, samples were cooled to room temperature and centrifuged at 13,000*g* for 10 min. Supernatants were collected and diluted to 4 M HCl. Hydroxyproline content was assessed using QuickZyme Sensitive Tissue Hydroxyproline Assay (QuickZyme Biosciences QZBTISHYP1) according to the manufacturer’s recommendations.

### Statistical analysis

Statistical analysis was performed using GraphPad Prism 9. Data are represented as the mean ± SEM. Statistical tests were applied as described in the figure legends. Statistical outliers were identified using the ROUT method (*Q* = 2 or 5%) and excluded from analyses.

## Notes

View/request a protocol for this paper from *Bio-protocol*.
