# Supplementary material for: Targeting oxidized phospholipids by AAV-based gene therapy in mice with established hepatic steatosis prevents progression to fibrosis
Source: Sci Adv. 2022 Jul 15;8(28):eabn0050. doi: 10.1126/sciadv.abn0050 (PMC9286512; doi:10.1126/sciadv.abn0050)
Supplement: Supplementary file 1 — Figs. S1 to S8 Tables S1 to S4 [file sciadv.abn0050_sm.pdf]

Science Advances
AAAS

Supplementary Materials for

Targeting oxidized phospholipids by AAV-based gene therapy in mice with established hepatic steatosis prevents progression to fibrosis

Clint M. Upchurch *et al.*

Corresponding author: Norbert Leitinger, nl2q@virginia.edu

Sci. Adv. **8**, eabn0050 (2022)
DOI: 10.1126/sciadv.abn0050

The PDF file includes:

Figs. S1 to S8
Tables S1 to S4
Legend for data file S1

Other Supplementary Material for this manuscript includes the following:

Data file S1

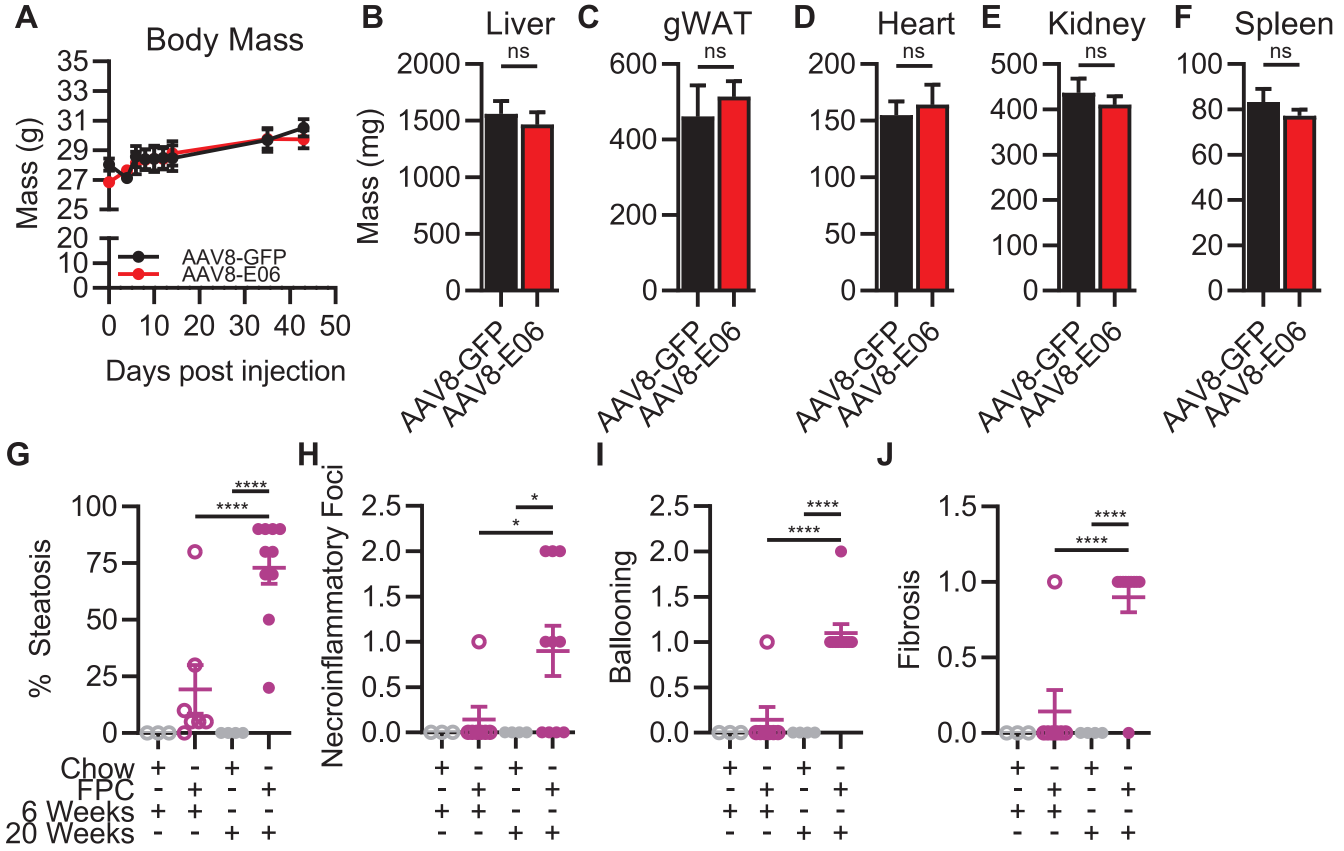
A
Body Mass
Mass (g)
AAV8-GFP
AAV8-E06
Days post injection
B
Liver
C
gWAT
D
Heart
E
Kidney
F
Spleen
Mass (mg)
ns
AAV8-GFP
AAV8-E06
G
% Steatosis
H
Necroinflammatory Foci
I
Ballooning
J
Fibrosis
Chow
FPC
6 Weeks
20 Weeks

Supplemental Figure 1. Adeno-associated virus serotype 8 scFv-E06 and FPC diet verification.

Mice were injected with either AAV8-GFP or AAV8-E06. There were no differences in **(A)** body mass, or mass of **(B)** liver, **(C)** gonadal white adipose tissue, **(D)** heart, **(E)** kidney, and **(F)** spleen (AAV8-GFP – n=5; AAV8-E06 – n=6). Mice injected with AAV8-GFP were fed chow or FPC diet for 6 or 20 weeks and histopathological assessment of **(G)** percent steatosis, **(H)** necroinflammatory foci, **(I)** hepatocellular ballooning, and **(J)** fibrosis was performed by a clinical pathologist. Statistical significance was determined by 2-way ANOVA (*=$p<0.05$, ****=$p<0.0001$).

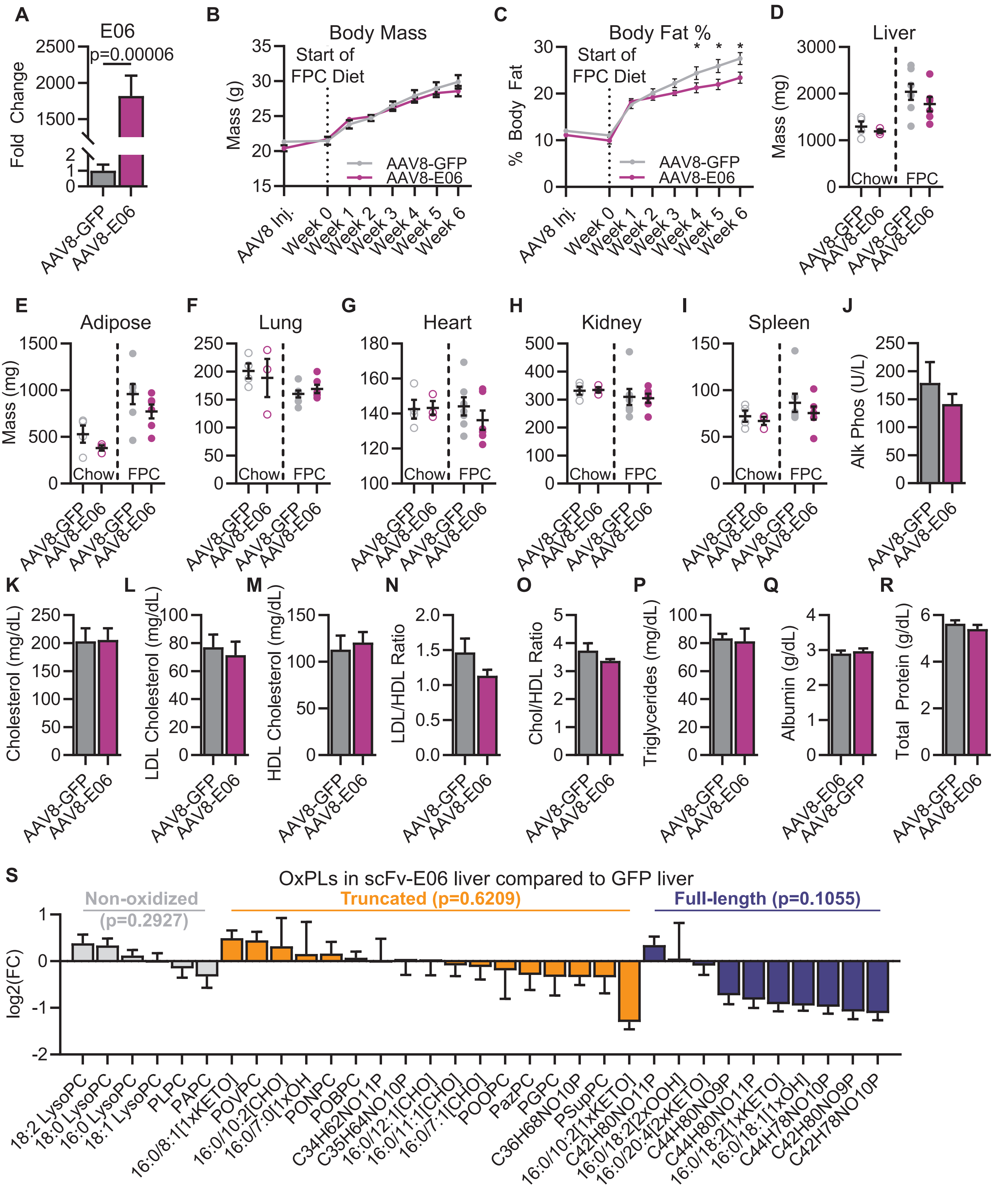
A
E06
p=0.00006
Fold Change
AAV8-GFP
AAV8-E06
B
Body Mass
Start of FPC Diet
Mass (g)
AAV8-GFP
AAV8-E06
AAV8 Inj.
Week 0
Week 1
Week 2
Week 3
Week 4
Week 5
Week 6
C
Body Fat %
Start of FPC Diet
% Body Fat
D
Liver
Mass (mg)
Chow
FPC
E
Adipose
F
Lung
G
Heart
H
Kidney
I
Spleen
J
Alk Phos (U/L)
K
Cholesterol (mg/dL)
L
LDL Cholesterol (mg/dL)
M
HDL Cholesterol (mg/dL)
N
LDL/HDL Ratio
O
Chol/HDL Ratio
P
Triglycerides (mg/dL)
Q
Albumin (g/dL)
R
Total Protein (g/dL)
S
OxPLs in scFv-E06 liver compared to GFP liver
Non-oxidized (p=0.2927)
Truncated (p=0.6209)
Full-length (p=0.1055)
log2(FC)
18:2 LysoPC
18:0 LysoPC
16:0 LysoPC
18:1 LysoPC
PLPC
PAPC
16:0/8:1[1xKETO]
POVPC
16:0/10:2[CHO]
16:0/7:0[1xOH
PONPC
POBPC
C34H62NO11P
C35H64NO10P
16:0/12:1[CHO]
16:0/11:1[CHO]
16:0/7:1[CHO]
POOPC
PazPC
PGPC
C36H68NO10P
PSupPC
16:0/10:2[1xKETO]
C42H80NO11P
16:0/18:2[2xOOH]
16:0/20:4[2xKETO]
C44H80NO9P
C44H80NO11P
16:0/18:2[1xKETO]
16:0/18:1[1xOH]
C44H78NO10P
C42H80NO9P
C42H78NO10P

Supplemental Figure 2. Characterization of mice treated with AAV8-scFv-E06 after six weeks of FPC diet.

Speer6-ps1$^{Tg(Alb\text{-}cre)21Mgn}$/J mice were injected with AAV8-GFP or AAV8-scFv-E06. Two weeks after injection, mice were fed FPC diet for six weeks (FPC AAV8-GFP, n=7; FPC AAV8-E06, n=6). **(A)** scFv-E06 mRNA expression was significantly increased in mice injected with AAV8-scFv-E06 (AAV8-GFP – n=6; AAV8-E06 – n=6). **(B)** Body mass (FPC AAV8-GFP, n=7; FPC AAV8-E06, n=6) and **(C)** body fat percentage (FPC AAV8-GFP, n=6; FPC AAV8-E06, n=6) were recorded weekly. While there was no difference in body mass, there was a significant decrease in body fat percentage between FPC diet-fed mice expressing scFv-E06 compared to mice expressing GFP after four weeks on diet. There was significant increase in body fat percentage between chow fed and FPC diet-fed mice after six weeks. After six weeks, mouse plasma was collected, and hepatic function and lipid profile were measured. (**D-I)** There were no significant differences in organ mass between AAV8-GFP and AAV8-scFv-E06 in either chow-fed or FPC-fed mice (FPC AAV8-GFP, n=7; FPC AAV8-E06, n=6). There was no significant difference in **(J)** alkaline phosphatase, **(K)** cholesterol, **(L)** LDL cholesterol, **(M)** HDL cholesterol, **(N)** LDL/HDL ratio, **(O)** cholesterol/HDL ratio, **(P)** triglycerides, **(Q)** albumin, and **(R)** total protein in the plasma of FPC diet-fed mice expressing scFv-E06 compared to GFP-expressing mice (AAV8-GFP – n=6; AAV8-E06 – n=6). **(S)** Truncated and full-length OxPCs extracted from liver tissue demonstrate a similar pattern to that observed in the plasma. Statistical significance was determined by 1-way and 2-way ANOVA and Student's T-test. Multiple comparisons were corrected by Dunnet or Tukey multiple comparisons correction (*=$p<0.05$, **=$p<0.01$, ***=$p<0.001$, ****=$p<0.0001$). Statistical outliers were excluded based on the ROUT test (Q=5%).

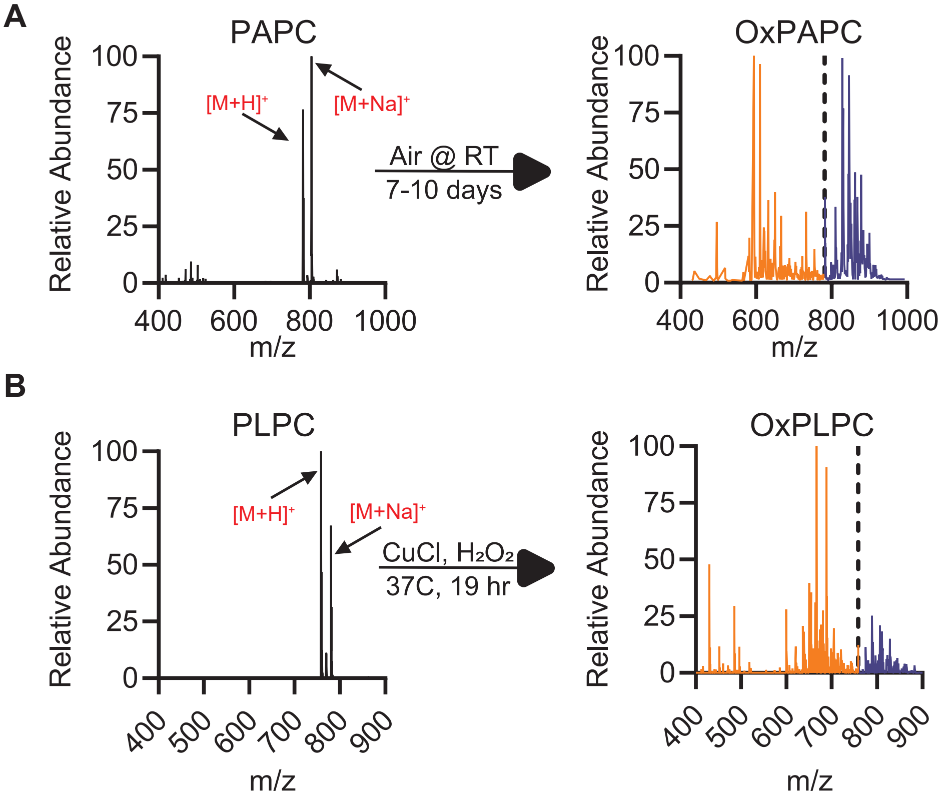

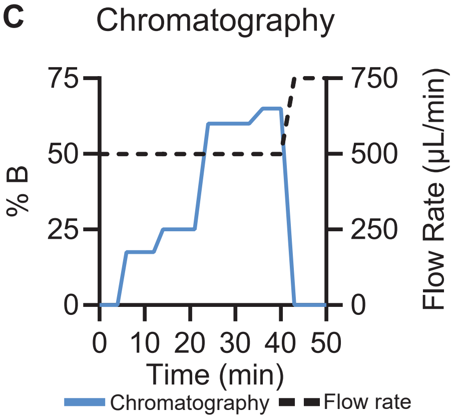

D

	Solvent A	Solvent B
Water	31%	---
Methanol	69%	50%
Isopropanol	---	50%
Ammonium acetate	10 mM	10 mM

Supplemental Figure 3. Oxidation and detection of oxidation products of PAPC and PLPC. Oxidized phospholipids were prepared for LCMS by *in vitro* oxidation. **(A)** PAPC was oxidized by air for 7-10 days. **(B)** PLPC was oxidized by Fenton-like copper reaction for 18 hours. **(C)** Schematic of chromatography for analysis mass spectrometric analysis of oxidized phospholipids. **(D)** Solvent system for separation of OxPL species by HPLC.

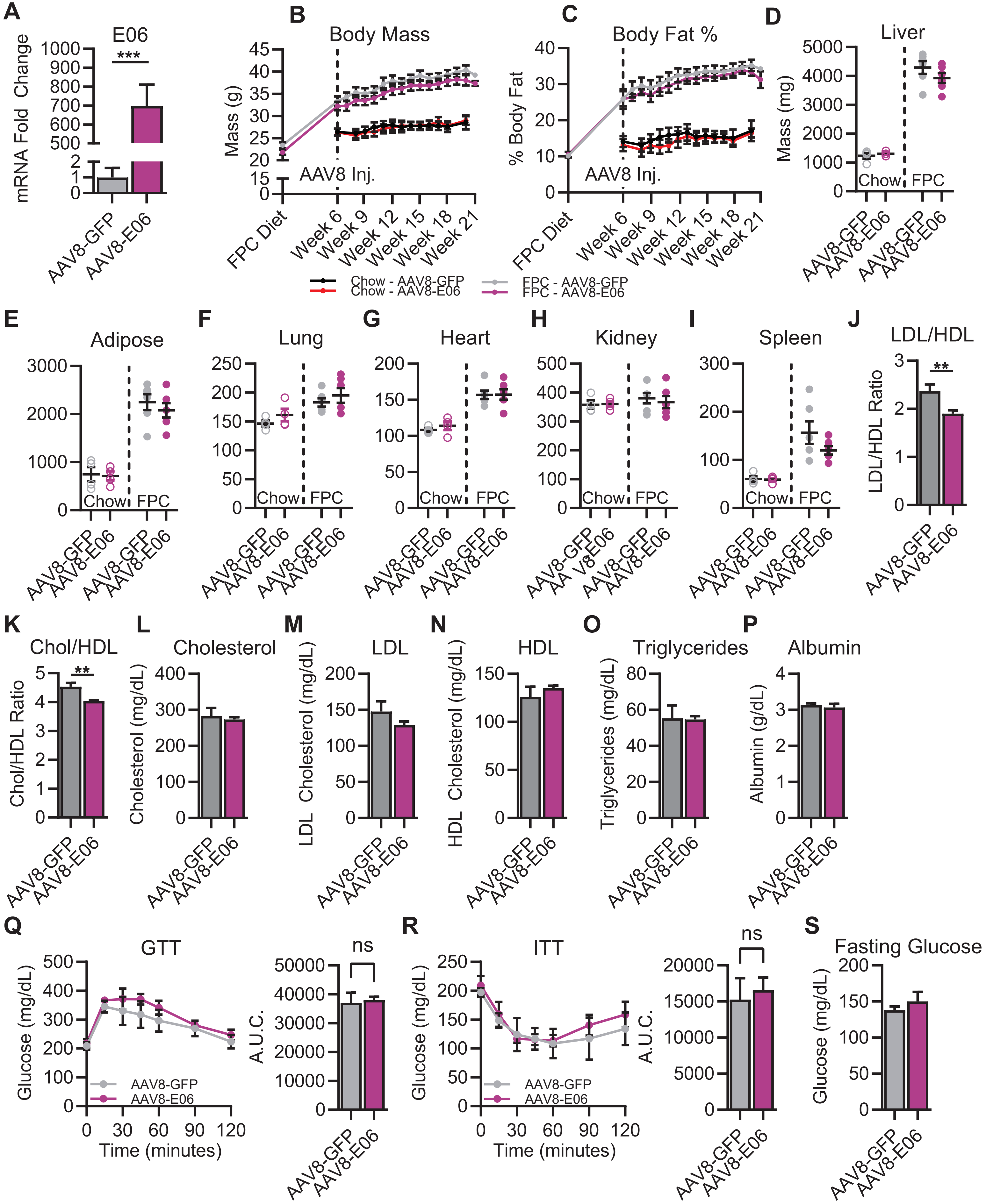

A
E06
***
mRNA Fold Change
AAV8-GFP
AAV8-E06
B
Body Mass
Mass (g)
AAV8 Inj.
FPC Diet
Week 6
Week 9
Week 12
Week 15
Week 18
Week 21
C
Body Fat %
% Body Fat
AAV8 Inj.
Chow - AAV8-GFP
Chow - AAV8-E06
FPC - AAV8-GFP
FPC - AAV8-E06
D
Liver
Mass (mg)
Chow
FPC
E
Adipose
F
Lung
G
Heart
H
Kidney
I
Spleen
J
LDL/HDL
LDL/HDL Ratio
**
K
Chol/HDL
Chol/HDL Ratio
**
L
Cholesterol
Cholesterol (mg/dL)
M
LDL
LDL Cholesterol (mg/dL)
N
HDL
HDL Cholesterol (mg/dL)
O
Triglycerides
Triglycerides (mg/dL)
P
Albumin
Albumin (g/dL)
Q
GTT
Glucose (mg/dL)
Time (minutes)
A.U.C.
ns
R
ITT
S
Fasting Glucose
Glucose (mg/dL)

Supplemental Figure 4. Gene ontology and gene expression of AML12 hepatocytes treated with truncated OxPAPC, full-length OxPAPC, and OxPAPC.

AML12 murine hepatocytes were treated with either OxPAPC, truncated OxPAPC, and full-length OxPAPC (100 μg/mL) for 4 hours and gene expression was measured via RNA-seq. GO Biological processes of upregulated genes (fold change > |1.5| and adjusted p-value < 0.05) identified by EnrichR for **(A)** OxPAPC, **(B)** truncated OxPAPC, and **(C)** full-length OxPAPC. Expression of **(D)** *Hmox1,* **(E)** *Pgd,* **(F)** *Acly,* **(G)** *Hmgcoas,* and **(H)** *Hmgcoar* in AML12 hepatocytes were confirmed by RT-qPCR (n=4). *Hmox1* and *Pgd* were regulated by all three oxidized phospholipid treatments; however, *Acly*, *Hmgcoas*, and *Hmgcoar* were exclusively upregulated by truncated OxPAPC. Statistical significance was determined by 1-way ANOVA. Multiple comparisons were corrected by Dunnet's multiple comparisons correction (*=p<0.05, **=p<0.01, ****=p<0.0001).

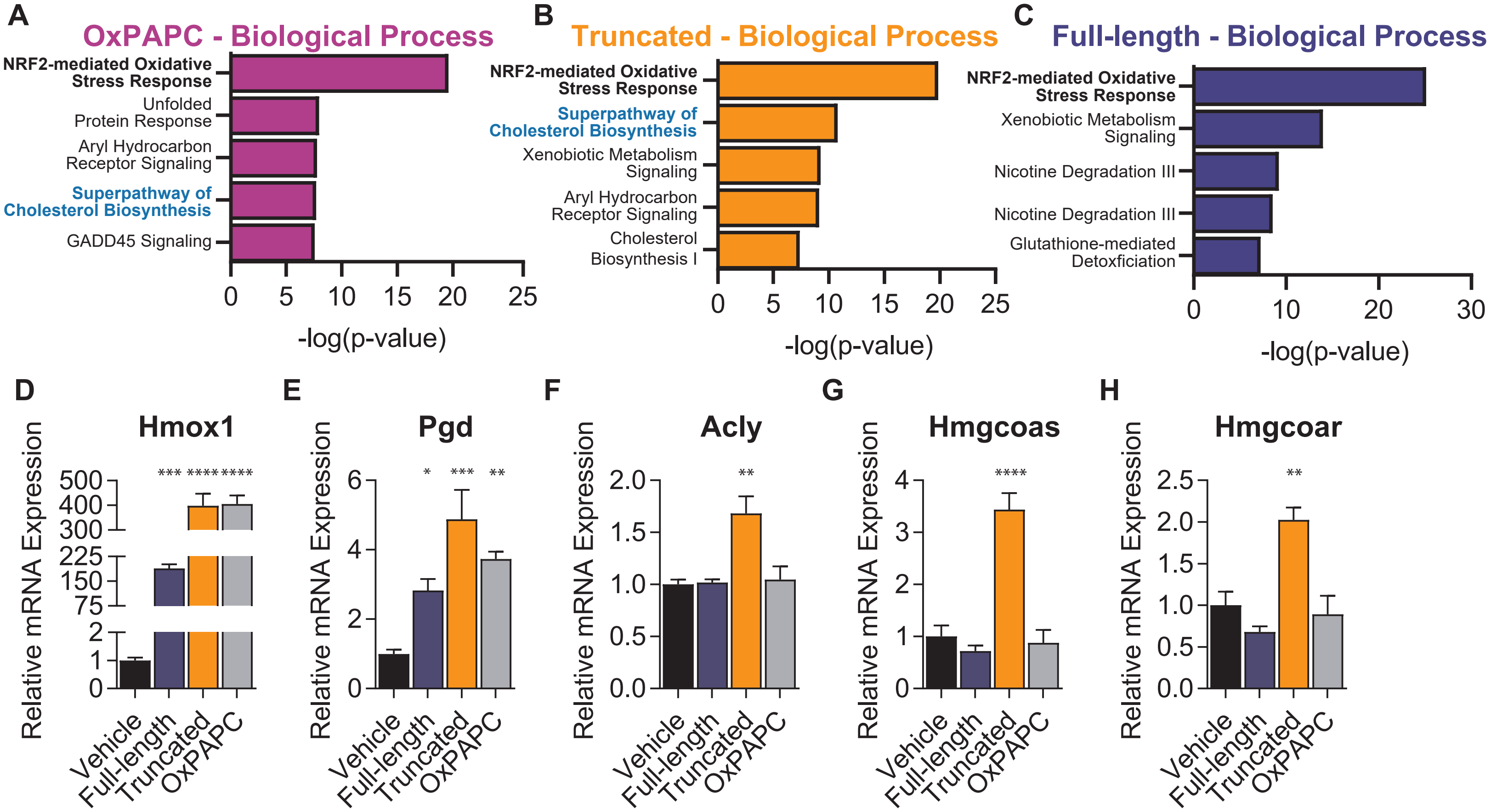
A
OxPAPC - Biological Process
NRF2-mediated Oxidative Stress Response
Unfolded Protein Response
Aryl Hydrocarbon Receptor Signaling
Superpathway of Cholesterol Biosynthesis
GADD45 Signaling
0 5 10 15 20 25
-log(p-value)
B
Truncated - Biological Process
NRF2-mediated Oxidative Stress Response
Superpathway of Cholesterol Biosynthesis
Xenobiotic Metabolism Signaling
Aryl Hydrocarbon Receptor Signaling
Cholesterol Biosynthesis I
0 5 10 15 20 25
-log(p-value)
C
Full-length - Biological Process
NRF2-mediated Oxidative Stress Response
Xenobiotic Metabolism Signaling
Nicotine Degradation III
Nicotine Degradation III
Glutathione-mediated Detoxficiation
0 10 20 30
-log(p-value)
D
Hmox1
E
Pgd
F
Acly
G
Hmgcoas
H
Hmgcoar
Relative mRNA Expression
Vehicle
Full-length
Truncated
OxPAPC

Supplemental Figure 5. Characterization of mice treated with AAV8-scFv-E06 after 20 weeks on FPC diet.

Speer6-ps1$^{Tg(Alb-cre)21Mgn}$/J mice were fed FPC or chow diet for six weeks. After six weeks mice were injected with either AAV8-GFP or AAV8-scFv-E06. Mice continued diet for a total of 20/21 weeks. **(A)** scFv-E06 gene expression was significantly increased in mice injected with AAV8-scFv-E06 ((FPC AAV8-GFP, n=5; FPC AAV8-E06, n=6). There were no significant differences in **(B)** body mass or **(C)** body fat percentage between GFP and scFv-E06 expressing mice on FPC or chow diet; however, there were a significant increase in both body mass and body fat percentage between diet groups (n=10). At the end of the experiment, plasma was collected, and hepatic function and lipid profile were measured. **(D-I)** There were no differences in organ mass between scFv-E06- and GFP-expressing mice fed chow or FPC diet (n=6). Ratios of **(J)** LDL/HDL and **(K)** Cholesterol/HDL in plasma were significantly reduced in mice expressing scFv-E06 compared to GFP after FPC diet feeding (n=6). **(L)** Cholesterol, **(M)** LDL, **(N)** HDL, **(O)** triglycerides, and **(P)** albumin levels in plasma were not significantly changed between scFv-E06 and GFP-expressing mice fed FPC diet (n=6). There were no differences in **(Q)** 6-hour fasted glucose and **(R)** insulin tolerance and **(S)** 18-hour fasting glucose in mice expressing GFP or scFv-E06 after 20 weeks FPC diet feeding (FPC AAV8-GFP, n=3; FPC AAV8-E06, n=4). Statistical significance was determined by 1-way and 2-way ANOVA and Student's T-test. Multiple comparisons were corrected by Dunnet or Tukey multiple comparisons correction (*=p<0.05, **=p<0.01, ****=p<0.0001).

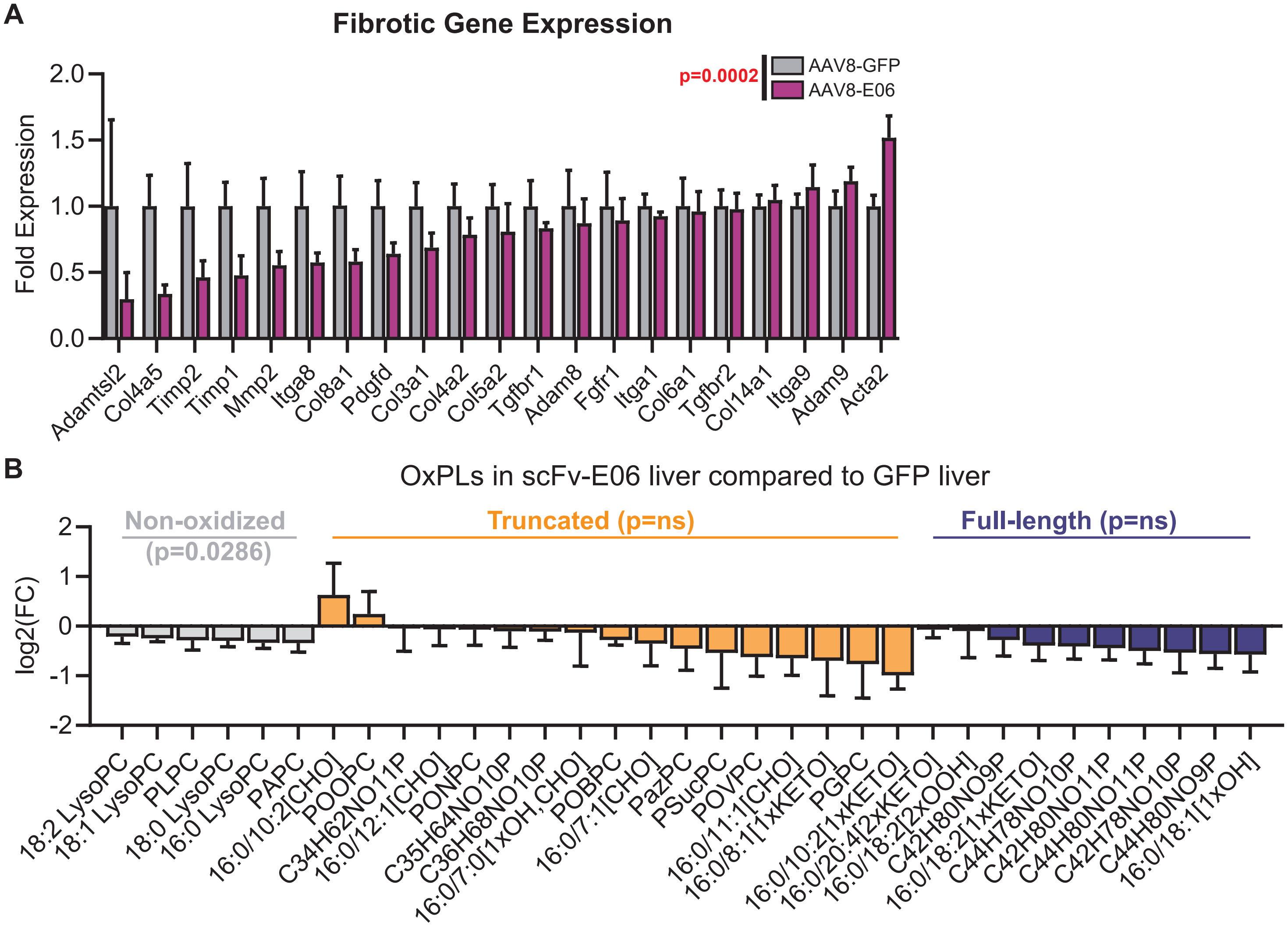
A
Fibrotic Gene Expression
Fold Expression
p=0.0002
AAV8-GFP
AAV8-E06
Adamtsl2
Col4a5
Timp2
Timp1
Mmp2
Itga8
Col8a1
Pdgfd
Col3a1
Col4a2
Col5a2
Tgfbr1
Adam8
Fgfr1
Itga1
Col6a1
Tgfbr2
Col14a1
Itga9
Adam9
Acta2
B
OxPLs in scFv-E06 liver compared to GFP liver
Non-oxidized (p=0.0286)
Truncated (p=ns)
Full-length (p=ns)
log2(FC)
18:2 LysoPC
18:1 LysoPC
PLPC
18:0 LysoPC
16:0 LysoPC
PAPC
16:0/10:2[CHO]
POOPC
C34H62NO11P
16:0/12:1[CHO]
PONPC
C35H64NO10P
C36H68NO10P
16:0/7:0[1xOH, CHO]
POBPC
16:0/7:1[CHO]
PazPC
PSucPC
POVPC
16:0/11:1[CHO]
16:0/8:1[1xKETO]
PGPC
16:0/10:2[1xKETO]
16:0/20:4[2xKETO]
16:0/18:2[2xOOH]
C42H80NO9P
16:0/18:2[1xKETO]
C44H78NO10P
C42H80NO11P
C44H80NO11P
C42H78NO10P
C44H80NO9P
16:0/18:1[1xOH]

Supplemental Figure 6. Fibrotic gene expression in mice treated with AAV8-scFv-E06.

(A) Hepatic gene expression of fibrogenic genes were confirmed by RT-qPCR (GFP – n=6, scFv – n=6). **(B)** Truncated and full-length OxPCs extracted from liver tissue demonstrate a similar pattern to that observed in the plasma, while non-oxidized phospholipids were significantly decreased. Statistical significance was determined by 2-way ANOVA with Dunnet's multiple comparisons correction. Statistical outliers were excluded with the ROUT test (Q=5%)

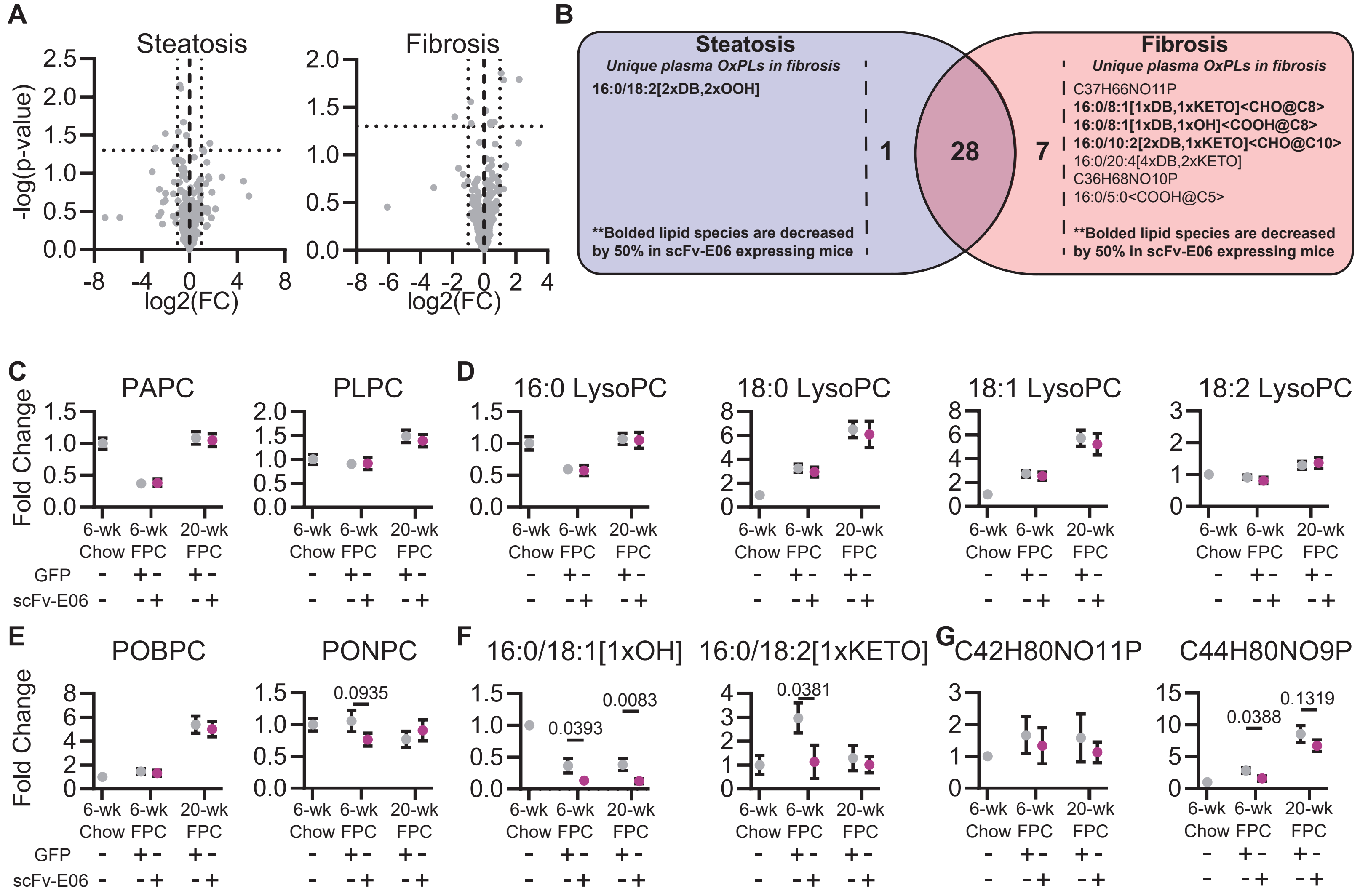
A
Steatosis
Fibrosis
-log(p-value)
log2(FC)
B
Steatosis
Unique plasma OxPLs in fibrosis
16:0/18:2[2xDB,2xOOH]
**Bolded lipid species are decreased by 50% in scFv-E06 expressing mice
1
28
7
Fibrosis
Unique plasma OxPLs in fibrosis
C37H66NO11P
16:0/8:1[1xDB,1xKETO]<CHO@C8>
16:0/8:1[1xDB,1xOH]<COOH@C8>
16:0/10:2[2xDB,1xKETO]<CHO@C10>
16:0/20:4[4xDB,2xKETO]
C36H68NO10P
16:0/5:0<COOH@C5>
**Bolded lipid species are decreased by 50% in scFv-E06 expressing mice
C
PAPC
PLPC
D
16:0 LysoPC
18:0 LysoPC
18:1 LysoPC
18:2 LysoPC
Fold Change
6-wk Chow
6-wk FPC
20-wk FPC
GFP
scFv-E06
E
POBPC
PONPC
0.0935
F
16:0/18:1[1xOH]
0.0393
0.0083
16:0/18:2[1xKETO]
0.0381
G
C42H80NO11P
C44H80NO9P
0.0388
0.1319

Supplemental Figure 7. Untargeted and targeted lipid analysis reveal a changing oxo-phospholipidome but no change in the overall lipidome.

The plasma lipidome of mice after **(A)** six (GFP – n=7, scFv-E06 – n=6) and twenty weeks (GFP – n=10, scFv-E06 – n=10) of FPC diet were unaffected by expression of scFv-E06. **(B)** Comparison of oxidized phospholipids species detected in steatosis and fibrosis revealed 1 unique plasma oxidized phospholipid in steatotic mice and 7 unique oxidized phospholipids in mice with hepatic fibrosis. **(C)** Non-oxidized phospholipids PAPC and PLPC were unaffected by expression of scFv-E06; though their levels were altered by FPC diet duration (6-wk Chow GFP – n=3, 6-wk FPC GFP – n=7, 6-wk FPC scFv-E06 – n=6, 20-wk FPC GFP – n=10, 20-wk FPC scFv-E06 – n=10). **(D)** 16:0 LysoPC (6-wk Chow GFP – n=3, 6-wk FPC GFP – n=7, 6-wk FPC scFv-E06 – n=6, 20-wk FPC GFP – n=10, 20-wk FPC scFv-E06 – n=10) decreased after 6-weeks of FPC diet feeding while 18:0, 18:1 (6-wk Chow GFP – n=3, 6-wk FPC GFP – n=7, 6-wk FPC scFv-E06 – n=6, 20-wk FPC GFP – n=7/6, 20-wk FPC scFv-E06 – n=7), and 18:2 LysoPC (6-wk Chow GFP – n=2, 6-wk FPC GFP – n=7, 6-wk FPC scFv-E06 – n=6, 20-wk FPC GFP – n=6, 20-wk FPC scFv-E06 – n=7) increased after 6 and 20 weeks on FPC diet compared to 6-week, chow-fed controls. **(E)** POBPC (6-wk Chow GFP – n=3, 6-wk FPC GFP – n=7, 6-wk FPC scFv-E06 – n=6, 20-wk FPC GFP – n=10, 20-wk FPC scFv-E06 – n=10) increased with longer duration FPC-feeding but was unaffected by scFv-E06, while PONPC (6-wk Chow GFP – n=3, 6-wk FPC GFP – n=7, 6-wk FPC scFv-E06 – n=6, 20-wk FPC GFP – n=10, 20-wk FPC scFv-E06 – n=10) was unaffected by feeding and scFv-E06 expression. **(F)** 16:0/18:1[1xOH] (6-wk Chow GFP – n=1, 6-wk FPC GFP – n=5, 6-wk FPC scFv-E06 – n=6, 20-wk FPC GFP – n=9, 20-wk FPC scFv-E06 – n=10) was decreased by expression of scFv-E06 at both 6-weeks and 20-weeks of FPC-diet feeding, while 16:0/18:2[1xKETO] was only decreased by scFv-E06 after six

weeks of feeding (6-wk Chow GFP – n=3, 6-wk FPC GFP – n=7, 6-wk FPC scFv-E06 – n=6, 20-wk FPC GFP – n=10, 20-wk FPC scFv-E06 – n=8). **(G)** The isobaric group C42H80NO11P (6-wk Chow GFP – n=3, 6-wk FPC GFP – n=7, 6-wk FPC scFv-E06 – n=6, 20-wk FPC GFP – n=10, 20-wk FPC scFv-E06 – n=10) was unaffected by diet and scFv-E06 expression while C44H80NO9P was increased after six and 20 weeks of FPC diet. scFv-E06 expression decreased levels of C44H80NO9P after 20 weeks of FPC diet (6-wk Chow GFP – n=3, 6-wk FPC GFP – n=7, 6-wk FPC scFv-E06 – n=6, 20-wk FPC GFP – n=10, 20-wk FPC scFv-E06 – n=10). Statistical significance was determined by 2-way ANOVA with Tukey multiple comparison correction or Student's t-test.

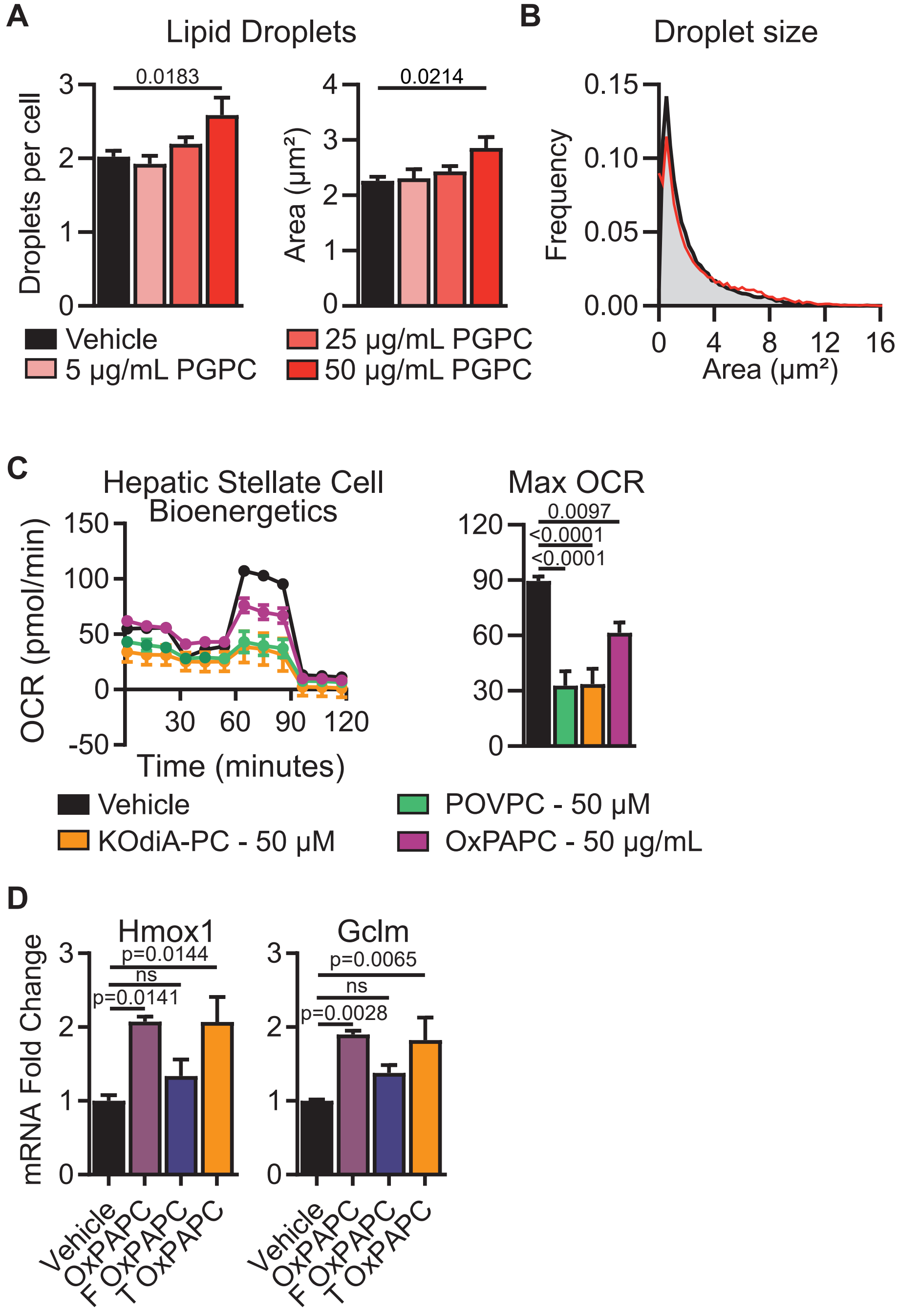

A
Lipid Droplets
0.0183
Droplets per cell
0.0214
Area (µm²)
Vehicle
5 µg/mL PGPC
25 µg/mL PGPC
50 µg/mL PGPC
B
Droplet size
Frequency
Area (µm²)
C
Hepatic Stellate Cell Bioenergetics
OCR (pmol/min)
Time (minutes)
Max OCR
0.0097
<0.0001
<0.0001
Vehicle
KOdiA-PC - 50 µM
POVPC - 50 µM
OxPAPC - 50 µg/mL
D
Hmox1
p=0.0144
ns
p=0.0141
mRNA Fold Change
Gclm
p=0.0065
ns
p=0.0028
Vehicle
OxPAPC
F OxPAPC
T OxPAPC

Supplemental Figure 8. Pathology-driving OxPCs regulate hepatocyte and hepatic stellate cell function *in vitro*.

AML12 hepatocytes treated with an increasing concentration of PGPC exhibited increased **(A)** lipid droplet number per cell and size resulting in a shift toward **(B)** a higher frequency of larger lipid droplets (n=4). **(C)** LX-2 hepatic stellate cell oxygen consumption rate was significantly decreased by POVPC, KOdiA-PC, and OxPAPC after 4 hours (Vehicle, n=6; POVPC, n=5; KOdiA-PC, n=5; OxPAPC, n=6), while **(D)** OxPAPC and truncated OxPAPC increased expression of *Hmox1* and *Gclm* after 4 hours (n=4). Statistical significance was determined by 1-way ANOVA with Dunnet's Multiple Comparison Correction or Student's t-test.

LPPTiger Nomenclature (previously identified species)	Parent Lipid	*m/z*	Formula	LPPTiger Predicted Structure(s)
1. PAPC	---	782	$C_{44}H_{80}NO_8P$	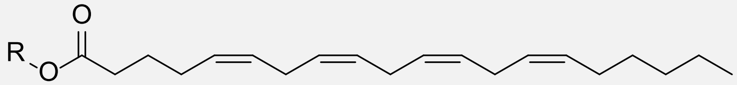
2. PLPC	---	758	$C_{42}H_{80}NO_8P$	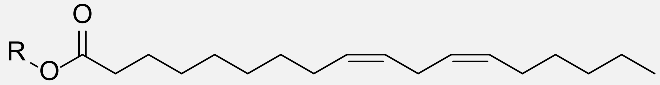
3. 16:0 LysoPC	---	496	$C_{24}H_{50}NO_7P$	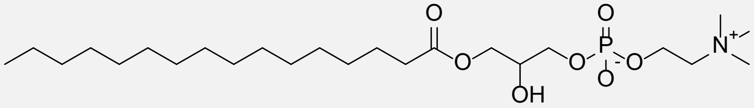
4. 18:1 LysoPC	---	522	$C_{26}H_{52}NO_7P$	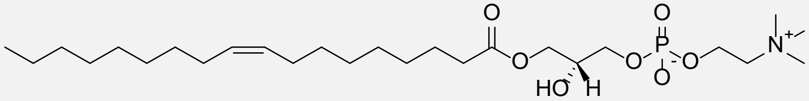
5. 18:2 LysoPC	---	520	$C_{26}H_{54}NO_7P$	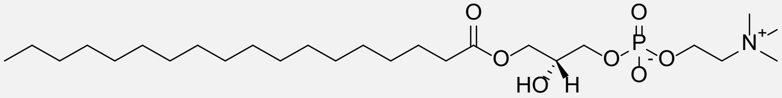
6. 18:0 LysoPC	---	524	$C_{26}H_{54}NO_7P$	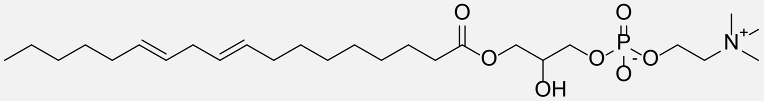
7. 16:0/12:1[CHO]	PLPC	690	$C_{36}H_{68}NO_9P$	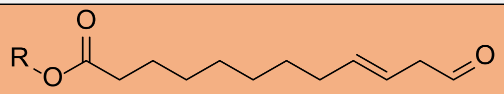
8. POBPC	PAPC	580	$C_{28}H_{54}NO_9P$	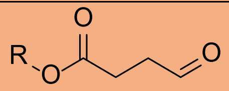
9. C35H62NO11P	PAPC	704	$C_{35}H_{62}NO_{11}P$	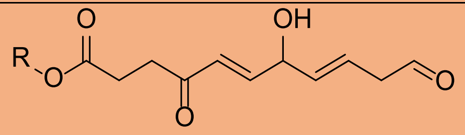 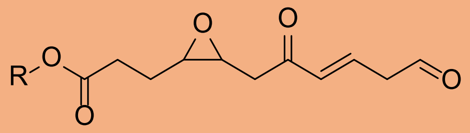 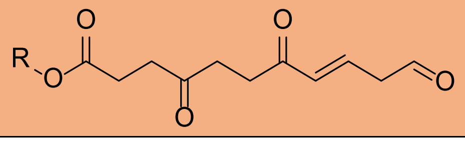

				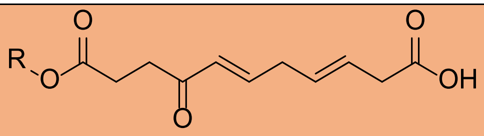
10. 16:0/7:0[1xOH,CHO]	PAPC	638	$C_{31}H_{60}NO_{10}P$	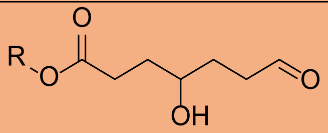
11. PONPC	PLPC	650	$C_{33}H_{64}NO_{9}P$	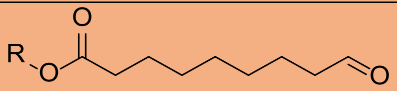
12. C35H64NO10P	PAPC	690	$C_{35}H_{64}NO_{10}P$	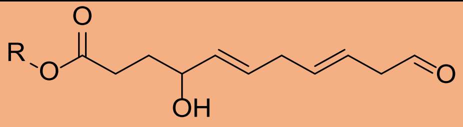 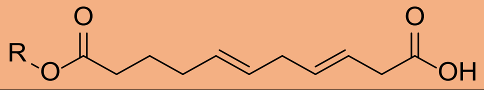
13. 16:0/11:1[CHO]	PLPC	676	$C_{35}H_{66}NO_{9}P$	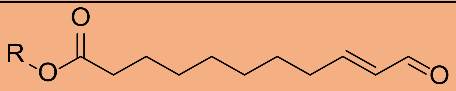
14. 16:0/8:0[CHO] (POOPC)	PLPC	636	$C_{32}H_{62}NO_{9}P$	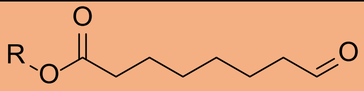
15. C35H64NO12P	PAPC	722	$C_{35}H_{64}NO_{12}P$	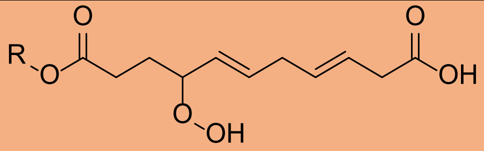 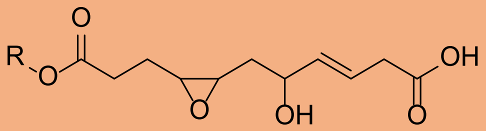 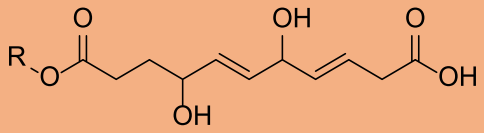 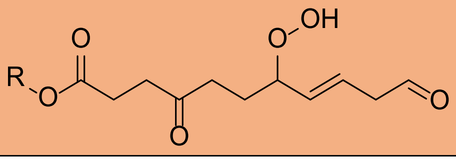

				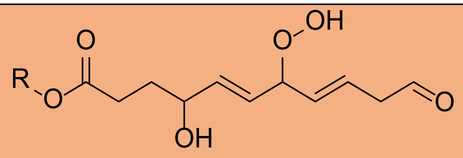 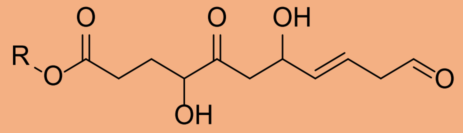 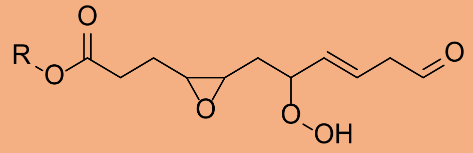 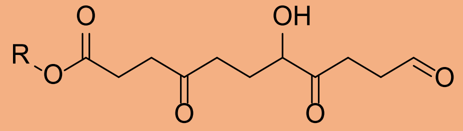 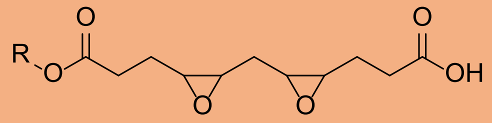 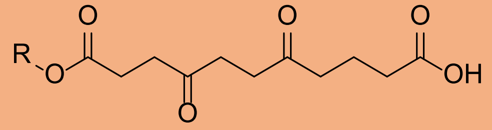 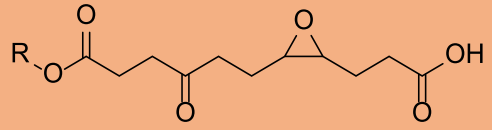 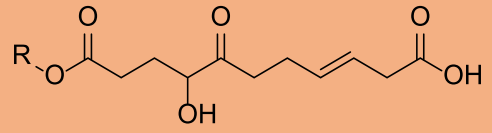 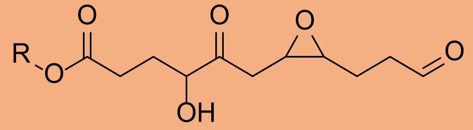
16. C32H60NO10P	PAPC	650	$C_{32}H_{60}NO_{10}P$	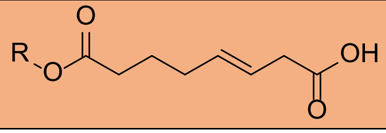

				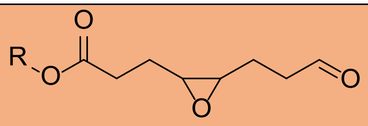<br>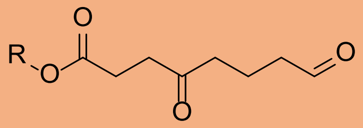<br>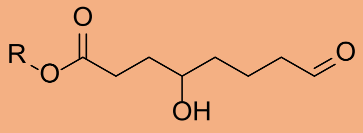
17. 16:0/10:2[CHO]	PAPC	660	$C_{34}H_{62}NO_9P$	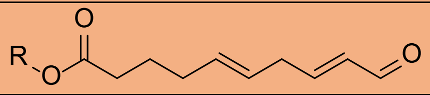
18. POVPC	PAPC	594	$C_{29}H56NO_9P$	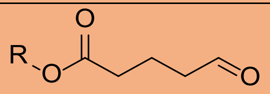
19. C34H62NO11P	PAPC	692	$C_{34}H_{62}NO_{11}P$	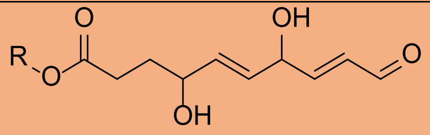<br>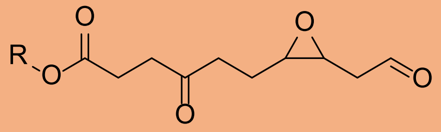<br>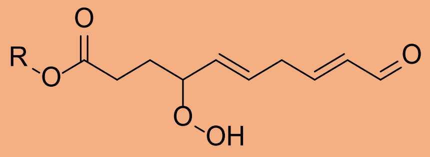<br>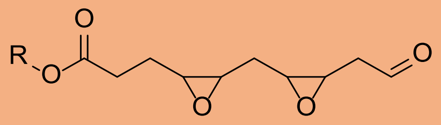<br>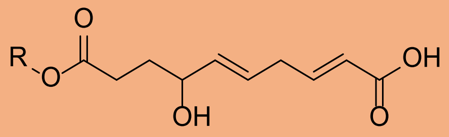<br>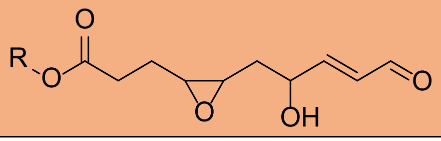

				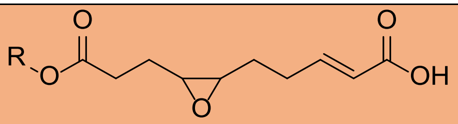 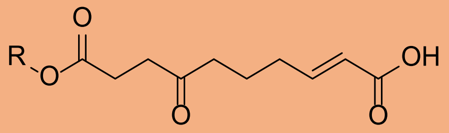 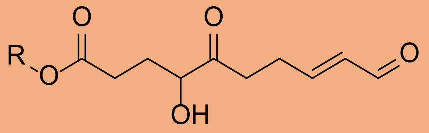 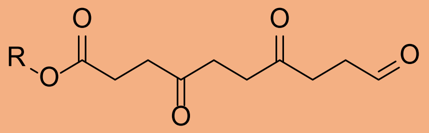
20. 16:0/7:1[CHO]	PAPC	620	$C_{31}H_{58}NO_9P$	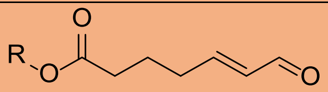
21. PazPC	PLPC	666	$C_{33}H_{64}NO_{10}P$	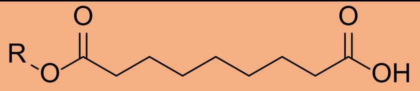
22. C42H80NO11P (HPODE-PC)	PLPC	806	$C_{42}H_{80}NO_{11}P$	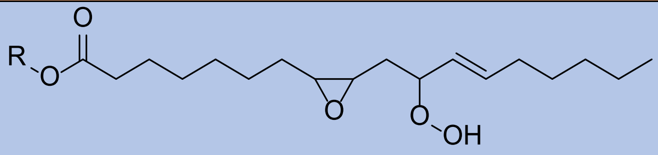 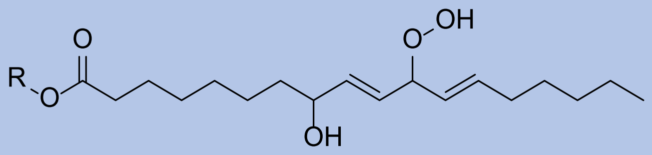 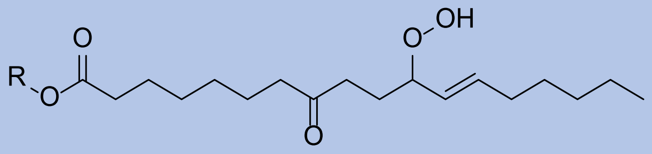
23. 16:0/18:2[2xOOH]	PLPC	822	$C_{42}H_{80}NO_{12}P$	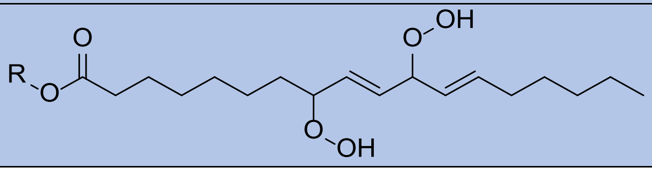

24. C42H78NO10P	PLPC	788	$C_{42}H_{78}NO_{10}P$	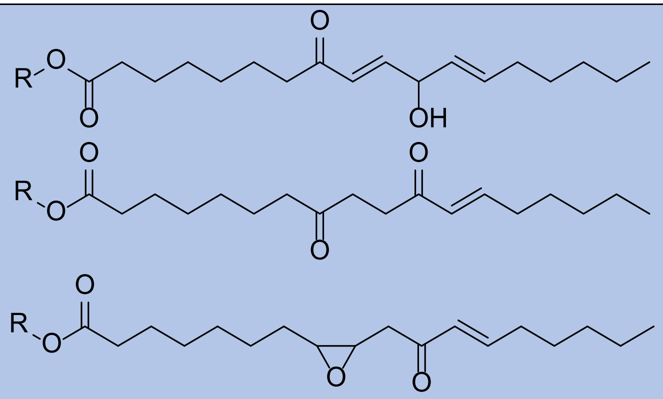
25. C44H80NO9P (HETE-PC)	PAPC	798	$C_{44}H_{80}NO_{9}P$	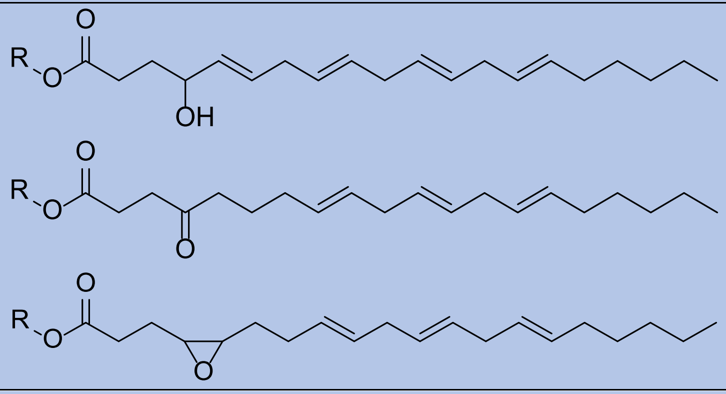
26. C44H80NO11P (Isoprostane-PC, Isoketal-PC)	PAPC	830	$C_{44}H_{80}NO_{11}P$	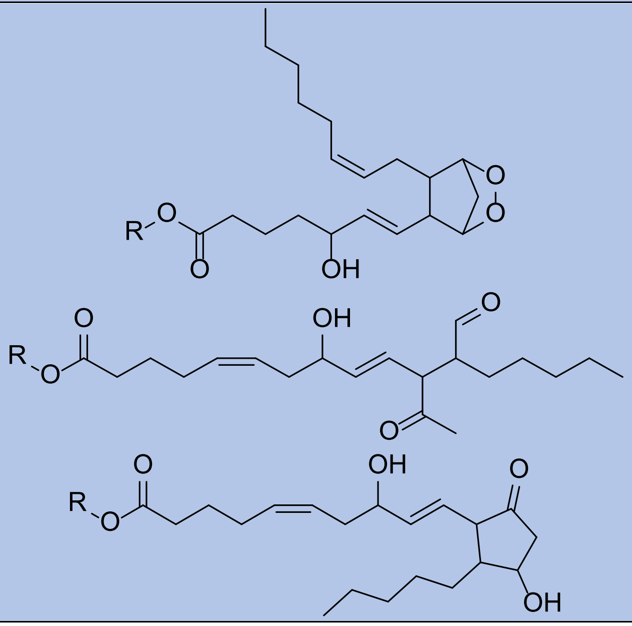

				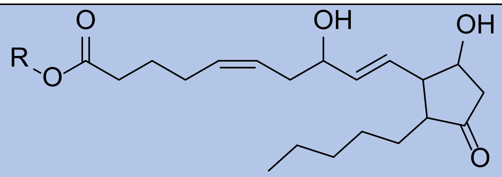
				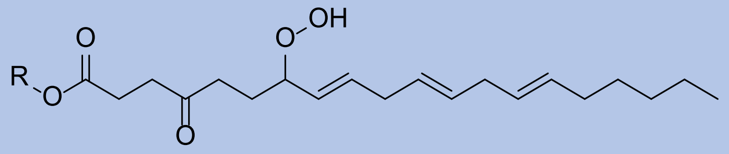
				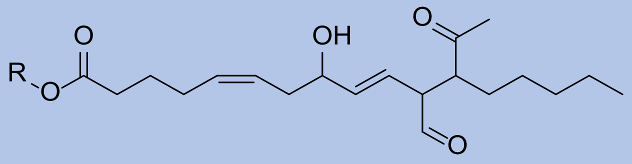
				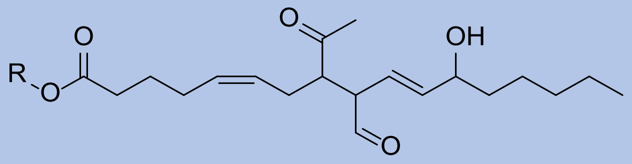
				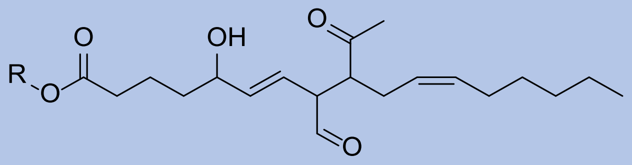
				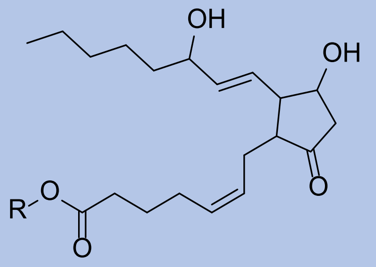
				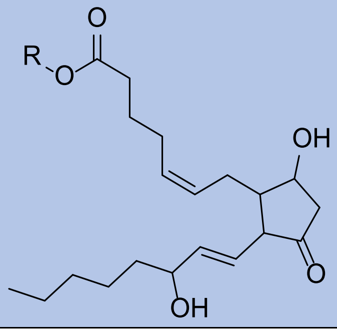

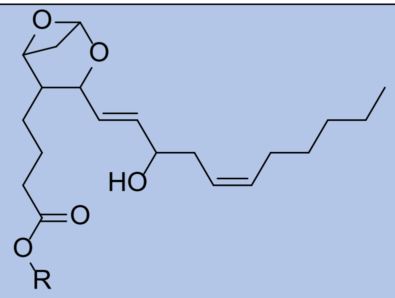
O
O
HO
O
O
R

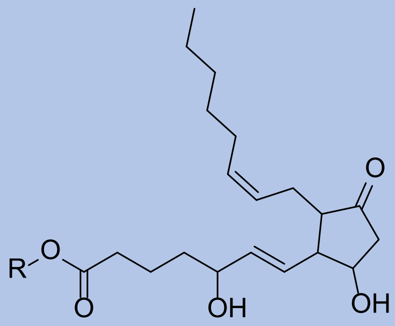
O
R
O
O
OH
OH

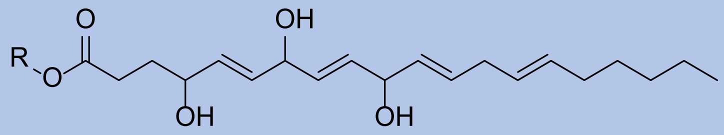
O
OH
R
O
OH
OH

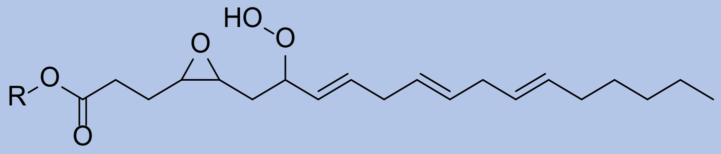
HO
O
O
O
R
O

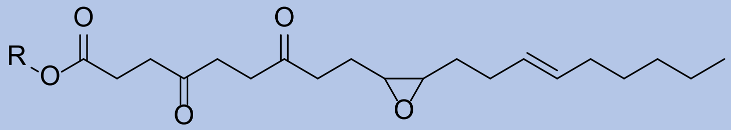
O
O
R
O
O
O

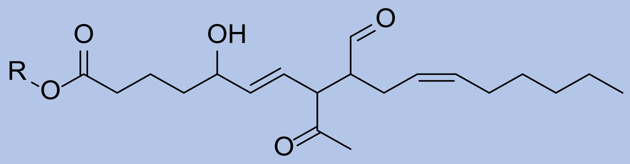
O
OH
O
R
O
O

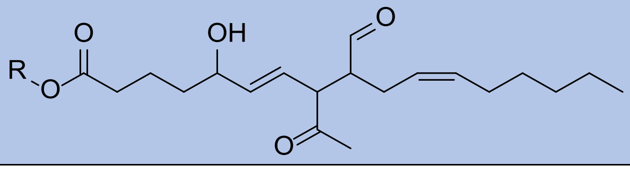
O
OH
O
R
O
O

				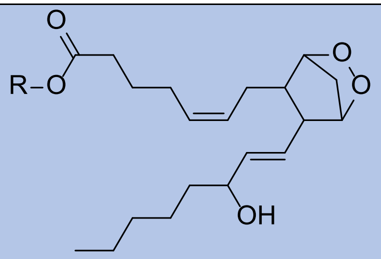<br>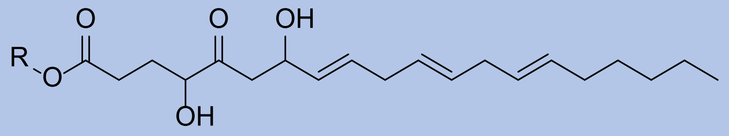<br>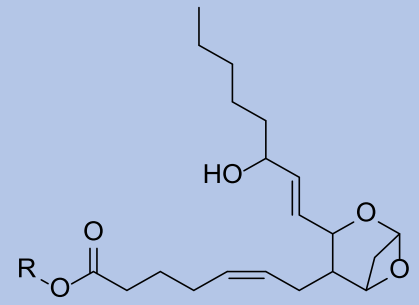<br>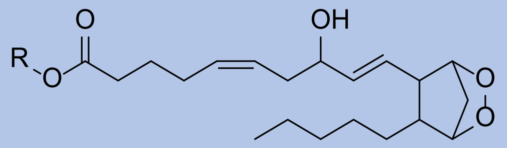<br>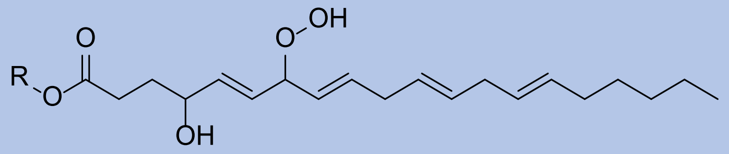<br>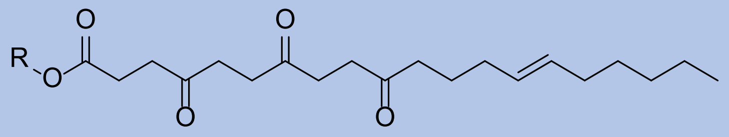<br>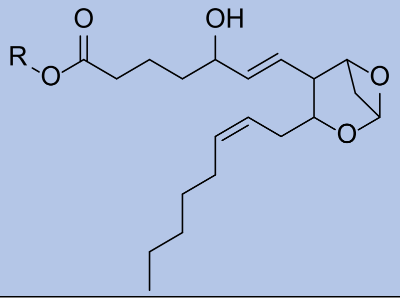

				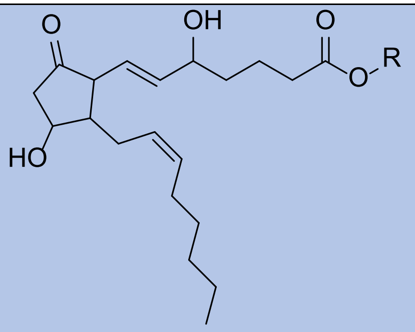<br>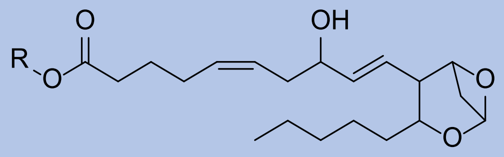<br>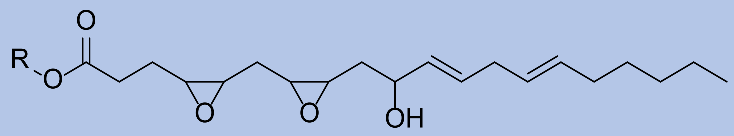<br>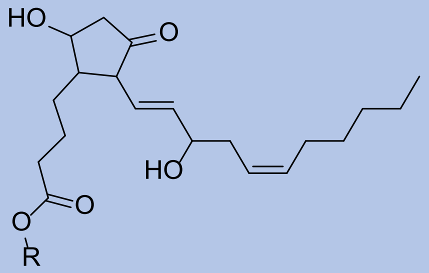<br>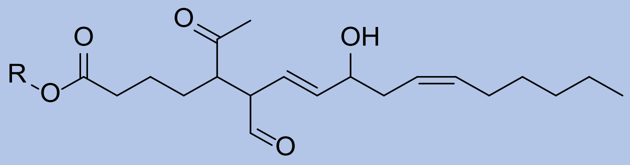<br>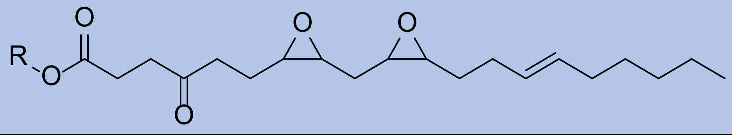

				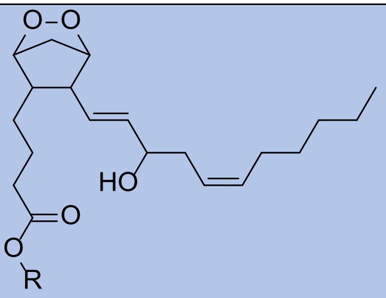 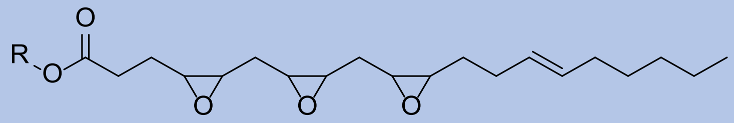 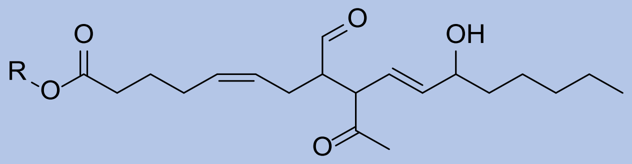 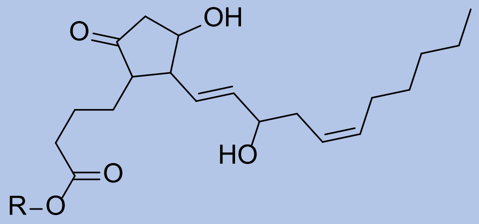 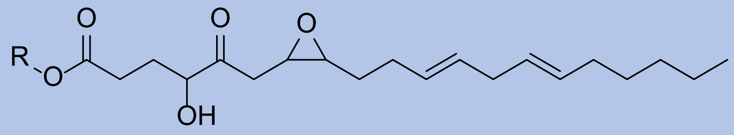 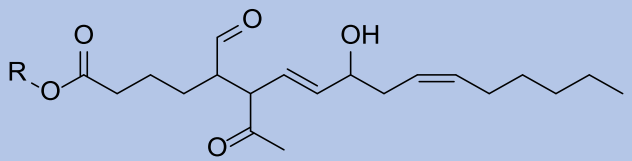 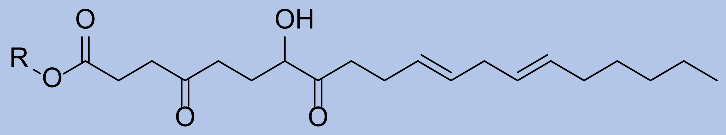
27. C42H80NO9P (HODE-PC)	PLPC	774	$C_{42}H_{80}NO_9P$	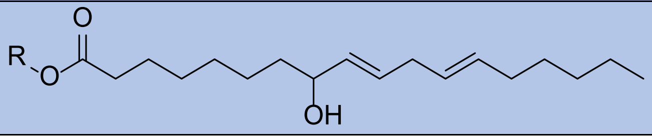

				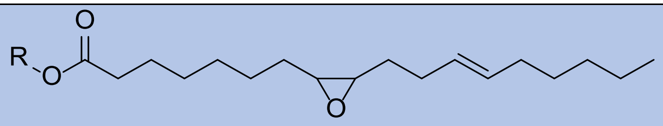<br>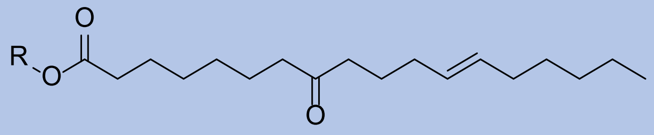
28. 16:0/18:2[2xDB,1xKETO] (KODE-PC)	PLPC	772	$C_{42}H_{78}NO_9P$	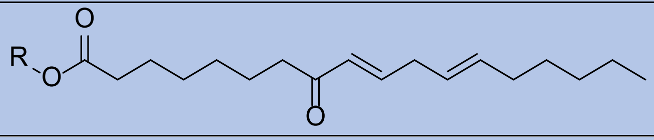
29. 16:0/18:1[1xDB,1xOH] (HOME-PC)	PLPC	776	$C_{42}H_{82}NO_9P$	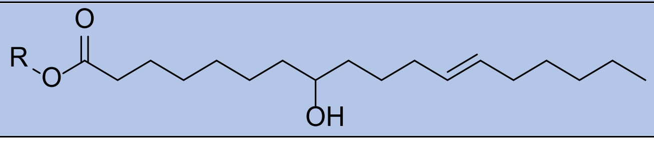

Supplemental Table 1. Oxidized phospholipids detected after development of hepatic steatosis. *In silico* predicted structures of oxidized phospholipids derived from PAPC or PLPC detected in mouse plasma after six weeks on FPC diet. CHO – aldehyde, COOH – carboxylic acid, OH – hydroxy, OOH – hydroperoxy, KETO – ketone, EPOXY – epoxide.

Gene Symbol	Trun-cated	Full-length	Ox-PAPC
1110002E22Rik	---	---	-1.06
1700011H14Rik	---	---	-0.83
1700066M21Rik	---	---	-0.74
1810011O10Rik	-1.11	---	---
2010111I01Rik	0.86	---	---
2310007B03Rik	---	---	-1.15
2310022B05Rik	---	---	-0.68
2410131K14Rik	0.77	---	0.86
2810417H13Rik	-0.77	---	-1.13
3010026O09Rik	---	---	-1.62
3110002H16Rik	---	---	0.72
3110043O21Rik	0.78	---	1.14
4921536K21Rik	---	---	1.63
4930427A07Rik	---	---	-0.82
4930430F08Rik	0.79	---	---
5031439G07Rik	---	---	0.75
5730508B09Rik	---	---	-0.97
9230110C19Rik	1.26	---	---
9330159F19Rik	---	---	0.97
9930012K11Rik	---	---	-0.95
AA415398	---	---	0.82
Aasdhppt	---	---	-0.75
Abca8b	2.50	1.43	2.74
Abcb6	2.03	1.35	1.77
Abcc1	1.90	1.58	2.02
Abcc2	3.72	2.81	4.08
Abcc4	1.53	1.37	1.71
Abcc5	1.03	---	1.04
Abcd4	---	---	0.87
Abhd4	1.36	1.03	1.64
Abhd5	---	---	0.92
Ablim1	-0.80	---	-0.77
Abr	1.05	---	1.20
Abtb1	1.24	---	1.02
Acan	-0.91	---	---
Acot2	1.01	---	1.38
Acox2	1.12	---	1.72
Acrbp	---	---	0.92
Actb	---	---	-0.67
Actg1	-0.72	---	-0.95
Actn1	-0.78	---	-0.72
Adamts1	-1.17	-1.36	---
Adamts6	-1.50	-1.58	-1.50
Adamts9	-1.73	-1.22	-1.73
Adap2	---	---	-1.26
Adgra2	-1.03	---	-0.81
Adgrg1	---	---	1.14
Adh7	2.75	1.86	1.86
Adprm	1.08	---	1.31
Adra1a	2.56	1.79	2.50
Adrm1	0.81	---	1.13
Afap1	---	---	0.85
Ahnak2	1.14	0.81	1.16
Ahr	-1.54	-0.92	-1.52
Ahsa1	0.79	---	0.84
Ahsa2	1.42	0.77	1.39
AI846148	---	---	0.88
Ajuba	---	---	-0.96
Akap1	---	---	-0.96
Akap12	-0.86	---	-0.99
Akap8l	0.79	---	1.05
Akr1c13	0.80	---	---
Akr1c14	2.30	1.37	1.73
Akr1c19	3.31	2.39	3.16
Akt1s1	---	---	0.78
Alas1	0.91	0.81	0.96
Aldh18a1	---	---	0.67
Aldh1a1	2.27	1.50	1.70
Aldh1l2	---	---	1.06
Aldh2	---	---	0.71
Aldh3a2	0.83	---	0.92
Aldoa	---	---	0.85
Alg6	---	---	-0.96
Als2	0.75	---	0.79
Amacr	---	---	-0.88
Ammecr1	---	---	-0.75
Amotl2	-0.86	---	-1.58
Ampd3	3.35	3.12	3.67
Anapc16	---	---	0.79
Angptl4	-0.97	---	-1.40
Angptl6	---	---	1.34
Ankle1	---	---	-1.24
Ankrd1	-2.23	-0.98	-2.75
Ankrd11	---	---	0.91
Ankrd12	---	---	1.05
Ankrd40	---	---	-0.63
Ankrd49	---	---	-0.80
Anln	-0.73	---	-0.91
Antxr2	-0.79	---	---
Anxa7	---	---	0.75
Aox1	2.67	2.07	2.79
Ap1ar	---	---	-0.76
Ap1s3	---	---	-0.84
Apba3	---	---	0.78
Apobr	2.59	1.14	3.37
Aqp9	1.84	---	2.08
Areg	---	---	1.37
Arhgap18	---	---	-0.82
Arhgap19	---	---	-1.35
Arhgap27	---	---	0.72
Arhgap8	-1.23	---	-1.22
Arhgef39	---	---	-1.01
Arid4b	---	---	0.70
Arid5a	---	---	1.36
Arih2	---	---	0.80
Arl4a	---	---	0.79
Arl4c	-1.13	-0.85	-1.65
Arl4d	1.19	---	---
Arl5b	---	---	0.93
Arl8a	---	---	0.95
Armcx3	---	---	0.96
Arrdc2	1.08	---	0.91
Arrdc3	0.66	---	---
Asns	0.69	---	0.78
Aspm	---	---	-1.38
Atad5	---	---	-1.02
Atf3	1.39	---	4.41
Atf4	---	---	1.11
Atf5	1.01	---	1.15
Atg12	---	---	0.65
Atg14	0.97	---	1.17
Atp10a	-1.01	---	---
Atp5sl	0.85	---	0.87
Atp6v0a1	---	---	1.09
Atp7a	---	---	0.79
Atxn1	---	---	-0.81
Atxn7l2	---	---	0.77
Aurkb	-0.73	---	-1.09
Avl9	---	---	0.82
B2m	---	---	0.73
B4galt7	---	---	0.75
B4gat1	---	---	-0.78
Bach1	1.63	1.01	1.98
Bag3	1.62	1.42	2.57
Bahcc1	-0.99	---	-1.36
Bahd1	---	---	0.65
Bbc3	---	---	0.86
Bbs12	---	---	-0.98

BC005537	0.71	---	0.92
BC025446	-0.88	---	-0.77
BC055324	---	---	-0.65
Bcar1	-1.31	---	-1.47
Bcl3	-0.86	---	---
Blcap	---	---	1.03
Blvrb	3.83	3.03	3.95
Bmf	---	---	-0.94
Bnip3	0.97	---	0.91
Bok	---	---	-1.16
Bora	-0.97	---	-1.31
Borcs5	---	---	-0.81
Brd2	---	---	0.89
Bsdc1	0.79	---	1.13
Btc	---	---	1.01
Bub1b	---	---	-0.88
C2cd4c	1.04	---	---
C2cd5	---	---	-0.80
Cachd1	2.24	1.67	2.61
Cacna1h	---	-1.03	-0.95
Cacnb2	-1.06	---	---
Cacybp	---	---	0.85
Cad	---	---	-0.83
Calcoco1	1.15	---	1.11
Calr3	2.88	2.24	2.91
Camkk1	---	---	-1.43
Camkk2	-0.89	---	-1.16
Camkmt	---	---	0.90
Capn5	-0.92	---	-0.74
Car15	---	---	1.41
Card10	-0.75	---	-1.45
Casc4	-0.97	---	---
Casp8ap2	---	---	-0.89
Cat	1.64	1.36	1.62
Cav1	-0.79	---	-0.76
Cav2	-0.88	---	-1.12
Cbr1	1.60	0.89	1.32
Cbx4	---	---	-0.81
Cbx6	---	---	-1.00
Cc2d2a	---	---	-0.86
Ccbe1	-1.42	---	-1.21
Ccdc117	0.83	---	0.75
Ccdc186	---	---	0.80
Ccdc28a	---	---	0.75
Ccdc71l	---	---	0.74
Ccdc88a	1.06	0.99	1.43
Ccdc92	---	---	0.79
Ccl2	-1.21	-1.12	-1.78

Ccna2	---	---	-0.99
Ccnb1	---	---	-1.05
Ccnb2	---	---	-0.84
Ccnd1	-0.96	---	-1.21
Ccnd3	-0.73	---	-0.93
Ccne1	-1.99	---	-2.41
Ccne2	-1.25	---	-1.64
Ccnf	---	---	-0.85
Ccng2	0.96	---	1.31
Ccsap	---	---	-1.36
Cd3eap	---	---	-1.11
Cd44	-0.88	---	---
Cd59a	0.94	---	1.06
Cd9	---	---	0.70
Cdc20	---	---	-0.87
Cdc25b	---	---	-0.74
Cdc25c	---	---	-0.91
Cdc42ep2	1.79	1.56	1.69
Cdc42ep5	---	---	0.93
Cdc6	-1.13	---	-1.35
Cdc7	-0.74	---	-0.99
Cdca2	---	---	-0.65
Cdca7	-1.22	---	-1.77
Cdca7l	---	---	-1.16
Cdk2	-0.75	---	-0.72
Cdk6	-0.84	---	-0.86
Cdkn1a	---	---	0.74
Cdkn2aipnl	---	---	-0.88
Cdkn2b	1.75	1.24	2.02
Cdkn2c	---	---	-0.79
Cdkn2d	---	---	-1.62
Cdr2l	---	---	0.95
Cds2	0.97	---	0.87
Cdt1	-0.87	---	-0.67
Cebpa	1.59	---	1.67
Cebpb	---	---	1.27
Cenpa	---	---	-1.10
Cenpf	---	---	-0.74
Cenpl	---	---	-0.78
Cep55	---	---	-0.85
Cep78	---	---	-0.71
Cep85l	---	---	1.61
Cep89	---	---	-0.69
Cfap43	-0.72	---	---
Cgn	---	---	-1.52
Cgnl1	1.48	1.15	1.53
Chac1	---	---	2.38
Chaf1b	-0.74	---	---

Chchd10	---	---	1.33
Chd2	---	---	1.12
Chka	-0.99	-0.76	-0.70
Chmp1b	---	---	0.73
Chn2	-0.86	---	---
Chordc1	0.97	0.82	1.06
Chpf2	2.13	1.39	2.03
Chrnb2	1.29	---	1.39
Chst15	---	---	-0.70
Cish	---	---	1.01
Ckap2	-0.77	---	-0.87
Clcf1	---	---	0.84
Clcn2	---	---	0.88
Clcn6	---	---	0.90
Cldn2	-0.80	---	-0.89
Clic4	---	---	0.93
Clip2	---	---	0.81
Clk1	---	---	0.82
Clk3	---	---	0.67
Clk4	---	---	0.80
Clmn	---	---	-0.79
Clspn	---	---	-0.73
Cnn2	-0.95	---	-1.18
Cnnm2	0.86	---	---
Cnot6	---	---	-0.68
Cnppd1	0.98	---	1.14
Cobl	---	---	0.93
Coil	---	---	-0.90
Col11a2	1.41	---	1.46
Col4a5	---	---	-0.80
Coro2a	---	---	1.01
Cotl1	---	---	-0.72
Cpeb2	---	---	1.13
Cpt1a	0.92	---	0.83
Cpt1b	---	---	0.87
Crb2	---	---	-1.19
Crebrf	---	---	0.90
Creg1	2.32	2.00	2.58
Creld1	0.90	---	---
Crem	0.84	---	1.41
Crim1	-0.72	---	-0.82
Crlf1	---	0.97	1.24
Crocc	---	---	-0.88
Crocc2	---	---	-1.12
Crtc1	1.05	0.94	1.34
Cryab	---	---	1.15
Csf1	---	---	0.73
Csnk1e	---	---	0.76

Csrnp2	0.72	---	0.80
Cstb	---	---	0.72
Ctdsp2	---	---	-0.67
Ctdspl	---	---	-0.90
Ctgf	-3.76	-2.09	-4.45
Cth	1.17	---	1.35
Ctns	---	---	0.97
Ctps	---	---	-1.04
Ctsb	---	---	0.65
Ctsd	0.92	---	1.07
Cxcl1	-1.26	---	---
Cxcl5	-1.53	-1.17	-2.39
Cyb5a	1.76	1.63	1.97
Cyb5r1	---	---	0.97
Cyld	---	---	-1.08
Cyp1a1	2.61	1.97	---
Cyp2j6	0.85	---	1.00
Cyp3a13	---	---	0.94
Cyp51	2.25	---	2.51
Cyr61	-1.36	---	-1.75
Cys1	---	---	1.17
D630003M21Rik	---	---	-1.42
D930048N14Rik	---	---	-1.10
Dact2	1.33	---	0.88
Dbt	---	---	-0.87
Dck	---	---	-0.84
Dcun1d3	---	---	1.01
Ddit3	1.60	---	2.82
Ddit4	0.98	---	2.10
Ddx11	---	---	-1.01
Ddx20	---	---	-0.88
Ddx41	---	---	-0.81
Ddx51	---	---	-0.72
Dedd2	2.46	1.48	2.77
Dennd4a	---	---	1.07
Depdc1a	---	---	-1.04
Depdc7	1.05	---	1.49
Deptor	1.06	---	0.91
Dgat2	---	---	1.09
Dhcr24	0.67	---	---
Dhodh	---	---	-0.86
Dhrs13	---	---	-1.09
Dhrs3	-1.34	-1.18	-1.91
Dhrs9	-1.26	---	---
Dimt1	-0.78	---	-1.08
Dip2b	---	---	0.81
Dkc1	-0.74	---	-0.80
Dlg5	---	---	0.99
Dlgap4	---	---	0.98
Dlk2	-1.04	---	-1.98
Dna2	-1.13	---	-1.22
Dnaja1	1.20	0.88	1.63
Dnaja4	1.51	0.99	2.74
Dnajb1	1.38	0.99	2.96
Dnajb2	1.47	0.84	1.41
Dnajb4	1.96	1.60	2.23
Dnajb9	---	---	1.48
Dnajc27	-1.16	---	-1.15
Dnase2a	1.46	1.19	1.40
Dnhd1	---	---	1.14
Dnph1	---	---	-1.00
Dock5	---	---	0.84
Dpp7	---	---	0.90
Dpy19l1	---	---	-0.97
Dsel	---	---	-0.79
Dtl	-1.29	---	-1.29
Dtx2	---	---	0.78
Dusp1	2.31	2.06	4.15
Dusp10	-0.79	---	-0.66
Dusp14	-1.07	---	-1.03
Dusp18	1.46	1.08	1.44
Dusp4	1.36	0.80	1.86
Dusp5	-1.21	---	---
Dusp6	---	---	0.98
Dusp7	-1.22	---	-0.84
Dut	---	---	-0.86
Dync1h1	---	---	0.77
Dync1li1	---	---	0.67
Dyrk1b	1.12	---	1.44
Dzip1l	---	---	-0.76
E2f6	0.96	0.89	1.08
E2f7	-0.88	---	-0.95
E2f8	-1.51	---	-1.61
E330009J07Rik	1.11	---	1.19
Eaf1	---	---	0.89
Ecm1	---	---	0.74
Ect2	-0.76	---	-1.03
Edn1	---	---	-1.95
Eea1	---	---	0.83
Eef2kmt	---	---	-0.71
Eepd1	-0.83	---	-1.18
Efcab8	---	---	0.95
Efnb1	1.00	---	1.22
Efnb2	-1.42	-0.68	-1.13
Egfr	-0.96	---	---
Egr1	-3.62	-2.38	---
Eid3	1.97	1.82	2.33
Eif2s2	---	---	0.83
Eif4ebp1	0.83	---	1.16
Elf1	---	---	1.01
Elmo1	---	---	0.97
Eml4	-0.86	---	-0.83
Emp1	-0.89	---	-0.80
Enc1	-1.22	---	-1.05
Endod1	---	---	-0.66
Engase	---	---	-1.29
Enpp1	-0.88	-0.84	-0.86
Enpp4	---	---	-0.83
Entpd5	1.52	1.07	1.46
Epb41	---	---	0.86
Epha7	---	---	-1.04
Ephx1	1.63	1.29	1.82
Eprs	---	---	0.73
Ercc6l	---	---	-0.80
Ereg	---	---	0.81
Eri1	---	---	-0.70
Eri2	---	---	-0.79
Ern1	---	---	1.00
Ero1l	---	---	0.77
Ero1lb	1.15	0.86	1.12
Errfi1	-1.20	-0.89	---
Esd	1.61	1.40	1.54
Espl1	---	---	-1.03
Esyt1	---	---	0.72
Etaa1	---	---	-0.97
Ets1	-0.84	---	-0.83
Exo1	-0.74	---	-1.01
Ext1	-0.78	---	-0.79
Eya2	---	---	1.14
Ezr	---	---	0.71
F2rl1	-1.35	---	-1.29
F3	-2.17	-0.89	-2.19
Faap100	-0.84	---	-0.98
Faap24	---	---	-0.84
Fadd	---	---	-0.83
Fads1	---	---	0.89
Fads2	0.99	---	1.23
Fam102a	---	---	1.09
Fam102b	-1.32	-0.87	-1.06
Fam118a	---	---	-0.78
Fam126a	---	---	0.74
Fam13a	2.35	1.44	2.43

Fam21	0.91	---	1.07
Fam214b	1.04	---	1.17
Fam219a	1.98	1.44	2.02
Fam63a	---	---	0.72
Fam64a	---	---	-1.08
Fam65b	-1.92	-1.29	-2.88
Fam73b	---	---	-0.76
Fam83d	---	---	-1.10
Fam83g	---	---	1.29
Fam83h	---	---	0.87
Fam84b	---	-0.83	---
Fanca	---	---	-0.80
Fancb	---	---	-0.92
Fancd2	---	---	-0.68
Fbxl20	0.77	---	0.76
Fbxo11	---	---	0.72
Fbxo30	1.01	---	1.11
Fbxo31	0.98	---	1.03
Fbxo32	1.06	---	0.89
Fbxo48	---	---	-1.23
Fbxo5	-1.08	---	-1.55
Fbxo9	1.06	0.82	0.82
Fchsd1	0.97	---	---
Fchsd2	-1.08	---	-1.09
Fdft1	1.32	---	1.44
Fech	---	0.79	0.80
Fen1	---	---	-0.93
Fermt1	-1.00	-0.75	-1.14
Fermt2	-0.73	---	-0.87
Fermt3	---	---	1.73
Fgd3	1.18	0.93	---
Fgf1	2.37	1.98	2.17
Fgfrl1	---	---	0.77
Fhdc1	---	---	1.10
Fhl2	-0.96	---	-1.22
Fhl3	-0.79	---	---
Fignl1	-0.71	---	-1.34
Fip1l1	---	---	0.74
Fjx1	---	---	-0.80
Flcn	---	---	0.77
Flrt2	-2.03	---	-1.72
Flrt3	---	---	0.91
Flywch1	1.13	---	1.00
Fndc4	---	---	-0.94
Fopnl	1.14	1.00	0.98
Fosl2	---	---	0.91
Foxj1	---	---	-1.10
Foxn3	---	---	1.02

Foxo3	---	---	0.88
Foxq1	1.74	1.46	1.45
Fpgt	0.78	---	---
Frmd4a	-0.85	---	---
Frmd6	-1.32	-0.80	-1.24
Fth1	1.66	1.64	2.14
Ftl1	1.37	0.89	1.52
Fxyd5	---	---	1.00
Fzd4	---	---	-0.90
Fzd7	1.09	0.75	1.06
G0s2	---	---	-1.12
G6pdx	0.79	---	1.23
Gaa	0.72	---	0.93
Gabarapl1	2.43	1.61	2.64
Gabpb2	---	---	-0.76
Gadd45a	1.68	---	2.29
Gadd45b	---	---	-1.32
Gart	---	---	-0.81
Gas2l3	---	---	-0.88
Gata3	---	---	1.52
Gba	---	---	0.73
Gbe1	1.44	0.91	1.60
Gch1	1.32	---	1.60
Gclc	3.72	3.31	4.12
Gclm	2.61	2.26	2.72
Gcnt2	---	---	0.76
Gcnt3	---	---	-0.91
Gdf11	---	---	0.89
Gdf15	1.93	1.48	4.41
Gemin4	---	---	-0.95
Gemin8	-1.11	---	-0.99
Ggta1	-0.71	---	-0.75
Ghdc	---	---	0.80
Ghitm	---	---	0.66
Gimd1	---	---	-0.79
Gjb1	1.21	1.18	1.59
Gjb3	-0.89	---	-1.55
Gla	0.95	---	1.11
Glis2	---	---	-0.92
Glmp	---	---	0.77
Glul	1.12	0.77	1.28
Glyat	2.50	2.01	2.81
Gm10073	---	---	1.23
Gm21949	---	---	-1.15
Gm21972	---	---	0.93
Gm42878	---	---	0.72
Gm42906	---	---	-1.22
Gm43518	---	---	-0.86

Gm43552	1.15	0.82	1.10
Gm45208	---	---	0.69
Gm7694	1.05	---	1.08
Gm8797	---	---	1.26
Gm9938	---	---	-0.94
Gmnn	---	---	-0.90
Gnal	---	---	0.77
Gpam	-0.77	---	-1.19
Gpcpd1	1.72	1.12	1.93
Gpd1l	---	---	-0.70
Gpi1	---	---	0.76
Gpr137b	0.81	0.88	1.12
Gpr180	---	---	-0.98
Gprc5a	-1.34	---	---
Gpt2	---	---	0.88
Gpx1	0.82	---	---
Gramd1a	---	---	0.79
Gramd3	---	---	0.96
Gramd4	-0.91	-0.71	-0.88
Grasp	---	---	0.80
Grik5	---	---	1.01
Grina	---	---	0.72
Grk6	---	---	-0.81
Grn	0.78	---	0.82
Gsg2	-0.99	---	-1.51
Gsr	1.36	1.26	1.48
Gss	2.37	1.60	2.38
Gsta1	7.09	6.46	6.79
Gsta3	3.11	2.35	2.96
Gsta4	5.13	4.49	4.69
Gstm1	1.91	1.55	2.28
Gsto1	---	---	0.77
Gstp1	2.35	1.63	1.76
Gtf2ird1	---	---	0.99
Gxylt2	-1.18	---	-1.13
H1f0	-1.11	---	-1.51
H2-DMa	1.39	---	1.40
Hap1	---	---	-0.90
Haus5	---	---	-1.07
Havcr1	-1.50	-0.92	-1.54
Hax1	---	---	0.87
Hbegf	-1.10	---	-0.90
Hbp1	0.97	---	1.24
Hdac4	---	---	0.74
Heatr3	---	---	-0.68
Heca	---	---	0.95
Hells	-1.38	---	-1.41
Herpud1	---	---	1.16

Hes1	-0.97	---	-1.41
Hfe	2.39	1.88	2.22
Hgs	---	---	0.87
Hhipl1	---	---	1.31
Hid1	---	---	1.39
Hist1h1c	1.16	---	1.05
Hist3h2a	0.96	---	0.81
Hivep2	-1.05	---	---
Hk2	-1.17	---	---
Hmga2	-0.84	---	---
Hmgcr	1.29	---	1.48
Hmgcs1	1.37	---	1.35
Hmmr	---	---	-0.87
Hmox1	6.60	6.03	7.55
Hnf1b	-1.02	---	---
Hnrnpr	---	---	-0.75
Homer3	0.97	0.71	1.13
Hook1	---	---	-0.76
Hpdl	---	---	-1.05
Hr	---	---	1.12
Hs3st3b1	1.17	---	---
Hsd17b7	---	---	0.87
Hsf2	---	---	0.81
Hsp90aa1	1.19	0.86	1.71
Hsp90ab1	0.81	---	1.21
Hspa1a	5.39	4.26	6.42
Hspa1b	5.38	4.36	6.45
Hspa2	---	---	0.80
Hspa4l	1.17	---	1.12
Hspa8	1.14	1.00	1.61
Hspb1	2.25	1.58	3.30
Hspb8	1.84	1.47	2.11
Hsph1	2.12	1.46	2.65
Htatip2	2.00	1.05	1.66
Htr1b	-1.25	---	-1.37
Hyal1	1.03	---	1.15
Hyal3	1.09	0.83	1.37
Icam1	-1.15	---	-0.85
Ick	1.64	1.52	2.04
Id2	-1.00	---	-1.45
Idh1	2.64	1.48	2.54
Idi1	1.27	---	0.98
Ier3	---	---	0.67
Iffo2	-0.72	---	-0.74
Ifi202b	0.85	---	1.47
Ifi203	---	---	1.03
Ifi47	---	---	-1.20
Ifit2	---	---	-1.44
Ifngr1	---	---	-0.74
Ifnlr1	---	---	-1.01
Igfbp3	-0.86	---	-0.67
Igip	---	---	-1.15
Igsf9	-0.90	---	-1.16
Igtp	1.52	1.24	1.33
Ikbkg	1.45	1.06	1.06
Ikzf2	---	---	-1.38
Il11	---	---	1.26
Il18rap	---	---	-1.01
Il1r1	---	---	-1.17
Il24	-2.11	---	-1.70
Il33	---	---	0.72
Il4ra	0.96	0.91	1.15
Il5ra	-2.91	-2.00	-3.48
Il7	---	---	0.99
Impact	1.41	1.00	1.75
Inhba	-1.43	---	-0.87
Inhbb	-1.15	---	-2.33
Inpp4b	-1.81	---	-1.21
Inpp5j	0.91	---	1.25
Insig1	2.28	---	3.06
Ipp	---	---	-0.93
Iqck	---	---	0.89
Iqgap3	-0.77	---	-0.89
Irs2	1.09	1.03	0.75
Itga3	-0.81	---	---
Itga6	---	---	-0.64
Itga7	1.33	1.09	1.84
Itgb6	-1.59	-1.04	-2.88
Itpripl1	---	---	-0.75
Itsn1	---	---	0.70
Jade2	-1.78	-1.07	-2.28
Jade3	0.85	---	1.07
Jag1	-1.18	-0.78	-1.12
Jmjd6	---	---	0.75
Jrk	---	---	-0.82
Jun	---	---	2.46
Junb	-1.18	---	-0.97
Jup	---	---	0.74
Kank3	---	---	-1.40
Kbtbd8	---	---	-1.19
Kcmf1	---	---	0.91
Kcnab1	---	---	-0.71
Kcnk5	---	---	-0.84
Kctd18	0.92	---	---
Kdm2a	---	---	0.83
Kdm3a	---	---	0.83
Kdm4a	0.83	---	0.89
Keap1	0.97	---	0.95
Kif11	---	---	-0.82
Kif14	---	---	-1.21
Kif15	---	---	-0.82
Kif18a	-0.81	---	-1.04
Kif1b	0.76	---	0.91
Kif20a	---	---	-0.63
Kif20b	---	---	-0.70
Kif21a	0.94	---	1.32
Kif23	---	---	-0.68
Kif24	---	---	-0.99
Kif2a	1.03	0.94	1.40
Kif2c	---	---	-0.71
Kif3c	0.83	---	1.10
Kif7	---	---	-1.21
Kifc3	---	---	-0.90
Kitl	1.07	0.83	0.85
Klc4	0.91	---	0.78
Klf11	0.89	---	1.17
Klf15	---	---	-1.62
Klf16	-0.95	---	-0.89
Klf4	---	---	1.43
Klf5	---	---	0.81
Klf6	---	---	0.69
Klf7	-0.99	---	-1.00
Klf9	---	---	1.02
Klhl15	---	---	0.73
Klhl21	---	---	0.74
Klhl24	1.46	---	1.58
Klhl26	---	---	0.79
Klhl5	---	---	-0.70
Krt7	---	---	-0.88
Krt80	-1.04	---	-1.83
Ksr1	1.26	0.97	1.19
L2hgdh	---	---	-0.68
L3mbtl2	---	---	-0.86
Lancl2	---	---	-0.63
Lancl3	1.28	1.24	1.13
Lasp1	---	---	-0.71
Layn	1.25	1.05	1.71
Lcmt2	---	---	-0.83
Ldlr	1.51	---	1.72
Lfng	---	---	-1.02
Lgals8	0.77	---	1.26
Lgalsl	---	---	-0.99
Lgr6	-1.36	---	-1.73
Lhfpl2	---	---	0.76

Lif	-1.20	---	-1.04
Lig1	-0.80	---	-0.87
Lin37	---	---	0.85
Lipe	0.95	---	1.03
Lmbr1l	---	---	0.72
Lmcd1	-1.96	-1.15	-2.26
Lmln	---	---	-1.45
Lmnb2	---	---	-0.86
Lonrf3	---	---	0.83
Lpar2	0.69	---	---
Lpcat1	-0.91	---	-1.37
Lpcat4	-0.84	---	-0.74
Lpin1	1.99	---	2.41
Lpin2	1.31	---	1.61
Lrig1	-0.76	---	-0.64
Lrp8	1.38	1.28	1.66
Lrr1	---	---	-1.20
Lrrc49	---	---	0.73
Lrrc8c	-1.21	---	---
Lrrc8d	---	---	0.97
Lrrfip2	---	---	0.94
Lrtm2	1.44	---	---
Lss	1.09	---	0.93
Lurap1l	---	---	-0.80
Ly75	---	---	-0.79
Ly96	---	---	1.27
Lyar	-0.76	---	---
Maff	---	---	1.07
Mafg	1.19	0.80	1.39
Mafk	---	---	0.96
Maml2	-0.96	---	---
Man2a1	---	---	0.68
Maoa	1.08	---	0.90
Map1b	---	---	1.44
Map1lc3b	1.13	---	1.34
Map2k6	-1.55	-0.95	-1.38
Map3k4	---	---	-0.69
Map4k4	---	---	0.78
Mapk9	0.78	---	0.96
Mapkapk5	---	---	0.72
Mars2	---	---	-1.09
Masp1	-0.92	---	-1.08
Mat2a	---	---	-0.97
Mb21d2	---	---	0.91
Mccc2	---	---	-0.90
Mcm2	-0.90	---	-0.87
Mcm3	-0.88	---	-0.91
Mcm4	-0.91	---	-0.90
Mcm6	---	---	-0.69
Mcoln1	---	---	0.73
Mdfic	---	---	0.65
Mdm1	---	---	-1.00
Mdm2	---	---	0.96
Mecom	-1.52	-1.07	-1.55
Mef2d	---	---	0.90
Melk	---	---	-0.79
Mettl21a	---	---	-0.83
Mettl7a1	1.85	1.46	1.76
Mettl7b	1.15	1.18	1.34
Mfsd6	0.90	---	1.25
Mgat3	---	---	-1.35
Mgea5	0.69	---	0.85
Mgst1	1.79	1.33	1.62
Mgst2	1.57	1.41	1.33
Mia3	---	---	0.68
Mib2	---	---	0.82
Mical2	-0.99	---	-0.81
Micall2	---	---	-0.85
Mid1ip1	---	---	-0.93
Mipol1	---	---	-0.70
Mkx	---	---	-1.02
Mlkl	0.85	0.80	1.14
Mllt11	1.13	1.31	2.20
Mlycd	---	---	-0.89
Mmachc	-1.16	---	-1.29
Mmp11	0.82	---	---
Mmp13	---	---	1.14
Mms22l	---	---	-0.83
Mndal	---	---	0.88
Mns1	---	---	-0.89
Mocos	2.53	1.75	2.86
Mocs1	1.11	---	1.23
Mogs	---	---	-1.09
Mplkip	---	---	-0.83
Mpp4	1.14	1.06	1.38
Mpzl3	---	---	0.83
Mras	---	---	0.91
Mrc1	-1.77	-1.04	-1.76
Mre11a	---	---	-0.68
Mrpl14	1.03	---	0.88
Mrps6	0.96	---	1.02
Msantd3	1.15	1.05	1.30
Msantd4	0.73	---	---
Msh6	-1.09	---	-1.53
Msmo1	1.54	---	1.51
Msrb3	---	---	-0.76
Mtcl1	-0.81	---	---
mt-Co1	---	---	1.06
Mthfd1	---	---	-0.71
Mthfr	1.19	---	1.31
Mtmr10	-1.72	-0.75	-1.70
Mtmr3	---	---	0.71
Mttp	---	---	0.88
Mturn	1.45	---	1.32
Mvd	0.92	---	---
Mxd1	---	---	0.95
Mxd3	-1.39	---	-1.89
Mxd4	0.73	---	---
Myc	-0.75	---	-0.63
Myo5c	0.75	---	0.72
N4bp3	---	---	-0.84
Naif1	---	---	0.82
Nampt	1.07	---	1.49
Nanos1	-1.19	---	-1.01
Napa	---	---	0.68
Napb	1.10	---	1.16
Napepld	---	---	-1.02
Narf	0.89	---	0.72
Nars	---	---	0.73
Nat6	1.05	---	1.29
Nbeal2	-0.78	---	-1.01
Ncaph	-0.68	---	-0.80
Ncbp2	---	---	-0.77
Ndel1	---	---	0.75
Ndrg1	1.60	0.85	2.41
Neil3	---	---	-0.81
Nek6	-0.84	---	-0.78
Net1	-0.95	---	-0.70
Neurl1b	---	---	-1.01
Neurl3	---	-1.06	---
Nfatc4	1.18	---	---
Nfe2l1	---	---	0.83
Nfkb2	-0.83	---	---
Nfkbia	-1.00	-0.77	---
Nfkbie	---	---	-0.75
Nfkbiz	-0.85	---	-1.05
Ngf	1.47	1.50	2.29
Nid1	0.95	1.26	1.36
Nif3l1	1.06	---	1.41
Noa1	---	---	-1.06
Nolc1	-0.73	---	---
Notch2	---	---	0.77
Npc1	---	---	0.91
Npcd	---	---	-0.99

Nploc4	---	---	0.94
Npnt	-0.93	---	-0.89
Nr1h4	-1.15	---	-1.29
Nr4a1	-1.21	---	---
Nrarp	---	---	-1.35
Nrep	0.98	---	---
Nrg1	-0.90	---	---
Nsdhl	1.29	---	1.28
Nsfl1c	---	---	0.69
Nuak1	---	---	-0.80
Nuak2	---	---	-1.44
Nub1	0.78	---	0.82
Nucks1	---	---	-0.67
Nudt12	0.95	---	---
Nuf2	-0.75	---	-1.03
Numbl	2.19	1.74	2.31
Nup107	---	---	-0.73
Nup210l	---	---	0.69
Nup37	---	---	-0.97
Nup85	---	---	-0.83
Nupl2	---	---	-1.10
Nupr1	1.55	---	2.14
Nusap1	---	---	-0.93
Nxt1	---	---	-0.83
Nyap1	---	---	1.09
Obfc1	-0.66	---	---
Oip5	---	---	-0.76
Olfr56	---	---	-1.19
Onecut1	---	---	-1.64
Osbpl9	---	---	0.69
Oser1	---	---	1.40
Osgin1	2.10	1.87	2.75
Otud4	-0.93	---	-0.79
P2ry1	---	---	-1.01
P4ha1	1.35	0.79	1.66
P4ha2	---	---	0.74
Pacsin2	---	---	0.72
Padi2	-0.81	---	-1.10
Pafah2	0.91	---	0.68
Pag1	-1.20	---	-1.35
Palld	-1.09	---	-0.85
Palmd	-0.96	---	-1.10
Pank1	---	---	-0.83
Papd5	---	---	0.74
Papd7	---	---	-0.66
Papss2	1.73	---	1.98
Parp1	---	---	-0.66
Parp8	-1.00	---	---

Parpbp	-0.79	---	-1.01
Pax3	---	---	0.97
Pbk	---	---	-1.19
Pcdh1	-0.79	---	---
Pcdh7	-1.60	-0.98	-1.41
Pcdh9	---	---	1.55
Pcsk9	1.50	---	1.35
Pcx	1.54	---	1.31
Pcyt2	0.84	---	0.74
Pdcd4	0.80	---	---
Pddc1	---	---	-0.83
Pde4b	---	1.38	1.09
Pdgfb	-1.72	-1.09	-2.37
Pdk4	1.35	---	1.12
Pdlim7	---	---	1.04
Pdss1	-0.77	---	-0.73
Pdxk	-1.02	-1.03	-1.36
Pex13	1.09	---	1.12
Pfas	---	---	-0.87
Pgd	2.41	1.99	2.39
Pgm2l1	0.91	---	0.77
Phf1	0.99	---	1.19
Phf10	---	---	1.04
Phf19	-1.62	-0.90	-2.02
Phlda1	---	0.84	1.67
Pif1	-0.91	---	-1.59
Pik3ap1	0.92	---	0.74
Pilra	-0.73	---	---
Pilrb1	-0.94	---	---
Pim3	---	---	-0.81
Pla2g4a	1.13	0.91	0.86
Plat	---	---	0.71
Plau	-1.47	---	-1.30
Plaur	---	---	0.85
Pld3	---	---	0.73
Plek2	-1.03	---	-0.80
Plekha2	---	---	-0.75
Plekha7	-0.98	---	-1.00
Plekho1	---	---	0.74
Plekho2	-0.73	---	---
Plk1	-0.72	---	-1.08
Plk3	1.50	1.29	3.27
Plk4	---	---	-0.72
Plod1	---	---	0.68
Plod2	1.32	0.82	1.84
Plscr2	---	---	-1.01
Plxnd1	-0.78	---	---
Pmaip1	---	---	0.77

Pmvk	1.37	---	1.17
Pnpla8	---	---	0.71
Pnrc1	1.07	---	1.81
Pold1	---	---	-0.83
Pole2	-1.01	---	-0.98
Polh	-0.81	---	-1.08
Por	---	---	0.74
Pou6f1	1.04	---	1.22
Ppard	1.09	1.02	1.73
Ppat	-0.76	---	-0.94
Ppl	-0.71	---	-0.96
Ppp1r13b	---	---	0.91
Ppp1r13l	---	---	-0.89
Ppp1r15a	1.17	---	2.72
Ppp1r3e	---	---	-0.87
Ppp3ca	---	---	0.89
Pqlc2	1.22	0.82	1.34
Prc1	---	---	-0.85
Prdm2	---	---	0.90
Prdx1	0.93	---	0.92
Prdx6	2.18	1.58	2.28
Prex2	---	---	-0.88
Prim1	---	---	-0.63
Prim2	-0.81	---	---
Prkci	-0.69	---	-0.84
Prob1	1.01	---	0.84
Procr	3.31	2.74	4.38
Prodh	---	---	-0.99
Prorsd1	---	---	-1.00
Proser2	1.75	1.65	2.02
Prox1	---	---	1.03
Prr13	1.16	1.05	1.67
Prss23	-1.24	-0.90	-2.03
Prx	---	---	0.83
Psap	0.84	---	0.79
Psmb3	---	---	1.00
Psmc6	---	---	0.74
Psmd11	0.90	0.78	1.12
Psmd4	---	---	0.79
Psmd5	0.90	0.72	0.84
Psme4	0.74	---	0.83
Psph	1.01	---	1.06
Ptger4	---	---	1.26
Ptgr1	1.42	---	1.11
Ptgs2	2.08	2.10	3.18
Ptprb	-0.90	---	-1.36
Ptrh2	---	---	-0.89
Purb	---	---	-0.71

Pvr	---	---	1.27
Pxmp2	-1.35	---	-1.31
Pxylp1	---	---	-1.24
Pycr1	---	---	0.73
Rab17	---	---	-1.05
Racgap1	---	---	-0.71
Rad18	---	---	-0.73
Rad23b	---	---	0.76
Rad51ap1	-0.73	---	-0.99
Radil	2.05	1.86	2.30
Raf1	---	---	0.76
Ralgps2	-0.84	---	-0.73
Ranbp6	---	---	-0.75
Rap2b	---	---	0.91
Raph1	---	---	1.32
Rasgrf2	-0.92	---	---
Rasgrp3	---	-0.86	-1.53
Rasl12	---	---	-0.84
Rassf6	1.37	1.18	1.50
Rassf8	0.78	---	0.69
Rbbp6	---	---	0.78
Rbm33	---	---	0.76
Rbpms2	---	---	0.80
Rccd1	---	---	-0.80
Rcl1	---	---	-0.66
Rdh10	---	---	-1.16
Recql	---	---	-0.91
Reep6	---	---	1.48
Relb	-1.21	-1.21	-1.04
Rem2	-1.20	---	-1.17
Reps1	---	---	0.90
Rfc3	-0.88	---	-0.71
Rfc5	-0.73	---	-0.92
Rgs17	---	---	0.83
Rgs20	-0.85	---	---
Rhbdd1	---	---	1.06
Rhbdf2	---	---	1.06
Rhob	---	---	0.89
Rhobtb3	---	---	-0.74
Rhpn2	1.10	0.86	1.14
Riok3	0.73	---	0.84
Rit1	1.46	0.90	1.48
Rmi2	-1.11	---	-1.08
Rnd1	-1.25	---	-0.75
Rnd2	1.09	---	1.04
Rnf115	---	---	0.95
Rnf145	-1.50	-0.76	-1.86
Rnf183	3.14	2.69	3.72

Rnf19b	---	---	1.05
Rnf39	-1.08	---	-1.42
Rnf41	---	---	0.68
Rock2	---	---	0.79
Ror1	-1.36	---	-1.39
Rora	1.38	---	2.18
Rorb	---	---	0.93
RP23-145I16.3	0.94	---	0.99
Rragd	1.36	---	1.28
Rrm1	---	---	-0.64
Rrm2	-0.75	---	-0.82
Rrp12	---	---	-0.93
Rrp1b	-1.06	---	-1.09
Rsrp1	---	---	0.75
Rtkn2	---	---	-1.10
Runx2	-1.10	---	-1.19
Rusc2	---	0.77	1.14
Rybp	---	---	0.95
Ryr3	---	---	1.78
S1pr2	-1.07	---	-1.12
Saraf	0.98	---	0.93
Sars	---	---	0.78
Sat1	---	---	1.13
Sbds	---	---	1.17
Sc5d	---	---	0.89
Scamp5	---	---	-1.00
Scarb1	0.85	---	0.89
Scd2	1.36	---	0.68
Schip1	---	---	-1.15
Scpep1	0.77	---	0.85
Sdpr	-0.84	---	-1.41
Sec24d	---	---	1.29
Sel1l3	0.92	---	1.28
Sema6c	1.06	---	---
Sept10	---	---	-0.73
Sepw1	1.19	---	---
Serinc3	---	---	0.86
Serpinb9	1.48	1.16	1.52
Serpinb9b	1.13	1.06	1.33
Serpind1	-1.63	---	-1.39
Serpinh1	---	---	0.74
Sertad2	---	---	0.80
Sertad3	0.91	---	0.97
Sertad4	-1.77	-1.21	-2.08
Sesn2	---	---	1.38
Sesn3	1.30	0.91	1.39
Sf1	---	---	0.71

Sfxn4	---	---	0.78
Sgk1	1.09	1.13	1.10
Sgk3	---	---	0.82
Sgtb	1.15	---	1.70
Sh3bgrl2	---	---	0.86
Sh3bp1	-1.07	---	-0.90
Sh3bp2	2.10	1.54	2.65
Sh3bp5	-0.72	---	-0.95
Sh3tc1	0.92	---	0.77
Shank2	---	---	-0.93
Shcbp1	---	---	-0.80
Shmt1	-0.89	---	-0.94
Siglecg	---	---	-0.98
Sim2	---	---	0.74
Six4	1.40	1.30	1.29
Six5	---	---	-1.03
Ska1	---	---	-0.84
Ska3	---	---	-0.84
Skp2	---	---	-0.83
Slbp	---	---	-0.80
Slc12a2	---	---	-0.78
Slc16a10	---	---	1.58
Slc16a13	1.05	---	0.82
Slc16a6	1.11	---	1.17
Slc19a2	---	---	0.77
Slc1a4	3.01	2.00	3.54
Slc20a1	---	---	1.09
Slc22a23	1.51	1.27	1.74
Slc22a4	1.91	1.51	2.07
Slc25a1	0.77	---	0.96
Slc25a12	---	---	-0.66
Slc25a15	---	---	-0.99
Slc25a27	---	---	1.04
Slc25a33	0.93	---	1.25
Slc25a45	---	---	1.00
Slc25a48	-1.01	---	-1.63
Slc27a6	-1.31	---	-1.13
Slc29a2	-1.45	---	-1.24
Slc2a1	0.97	0.77	1.72
Slc2a2	---	---	-1.47
Slc30a1	---	---	-1.05
Slc35b2	-0.97	---	-1.15
Slc35d1	---	---	0.95
Slc35e2	---	---	0.81
Slc35e4	---	---	0.96
Slc35g1	---	---	-0.71
Slc36a4	---	---	-0.77
Slc38a2	---	---	0.97

Slc38a3	1.20	1.08	1.16
Slc38a7	0.92	---	1.24
Slc39a4	0.83	---	1.11
Slc3a2	1.83	1.22	2.56
Slc40a1	1.81	1.45	1.46
Slc43a2	0.93	---	0.98
Slc48a1	2.55	2.42	3.04
Slc5a3	1.20	---	1.30
Slc5a6	---	---	-0.96
Slc6a8	---	---	0.65
Slc6a9	2.12	1.32	2.83
Slc7a1	---	---	0.97
Slc7a11	5.19	4.54	5.63
Slc7a5	---	---	0.80
Slc8b1	1.28	---	1.02
Slco3a1	---	---	-0.71
Slco4a1	-1.17	-1.06	-0.98
Slfn2	-2.96	-1.76	-3.38
Slfn9	-1.72	---	-1.77
Sltm	---	---	0.95
Slu7	---	---	0.68
Smad5	---	---	-0.64
Smad7	-0.84	---	-0.84
Smcr8	---	---	0.98
Smim14	0.83	---	0.88
Smim3	---	---	0.97
Smox	---	---	0.75
Smpd3	-0.85	---	-0.99
Smtn	-1.01	---	-0.66
Smtnl2	---	---	-0.85
Smyd4	1.18	0.80	0.80
Snai2	-1.35	---	---
Snca	2.05	1.25	2.21
Snx10	1.33	0.93	1.37
Snx30	0.80	---	1.13
Soat2	1.41	---	2.24
Socs5	-0.84	---	-0.93
Sod1	0.97	---	1.11
Sorbs2	---	---	-0.78
Sord	0.91	---	0.74
Sox13	-0.97	---	-1.00
Spata1	---	---	0.98
Spc25	---	---	-1.02
Spcs3	---	---	-0.88
Spns2	-0.99	---	-1.33
Spns3	---	---	-1.17
Spp1	-0.76	---	-0.67
Sppl2b	---	---	-0.79

Sprr1a	-1.57	---	---
Spry1	---	---	-0.78
Spry2	-0.85	---	-0.95
Spry4	---	---	0.95
Spsb4	-1.12	---	-1.82
Sqle	1.63	---	1.61
Sqrdl	2.25	1.72	2.06
Sqstm1	2.77	1.85	3.27
Srd5a1	-0.89	---	-0.99
Srebf2	---	---	0.78
Srf	-1.01	---	-1.17
Srgap3	-1.87	-1.39	-1.77
Srm	-0.86	---	-0.87
Srr	---	---	1.20
Srrm4	---	---	0.86
Srsf10	---	---	-0.73
Srxn1	4.29	3.51	4.90
Ss18l1	---	---	0.83
Ssbp2	-0.92	---	-0.77
St13	---	---	0.89
Stambpl1	-1.09	---	-0.73
Stard13	-0.82	---	-1.20
Stard4	1.19	---	0.91
Stard9	---	---	0.98
Stat1	---	---	-0.94
Stat2	0.86	---	0.79
Stip1	---	---	1.00
Stk10	0.75	---	1.26
Stk40	---	---	0.68
Stx3	---	---	0.78
Stxbp1	---	---	1.04
Stxbp6	---	---	-0.86
Styk1	0.86	---	0.85
Suv39h2	---	---	-0.75
Swap70	---	---	0.67
Swt1	---	---	0.99
Syvn1	---	---	0.73
Tacc2	1.23	0.72	1.39
Tada1	---	---	-0.75
Tada2a	-0.72	---	-0.72
Taf7	1.46	1.23	2.04
Taldo1	0.91	---	0.87
Tapbpl	0.85	---	0.78
Tbc1d1	-0.77	---	---
Tbc1d9	---	---	0.97
Tbce	---	---	0.67
Tbcel	1.05	0.81	1.15
Tbl2	---	---	-1.04

Tcf19	-1.23	---	-1.44
Tcp11l2	1.00	---	1.26
Tdrp	1.14	---	1.06
Tef	0.86	---	---
Tex9	-1.04	---	-0.92
Tfap2a	1.03	---	1.58
Tfe3	---	---	1.21
Tfrc	-1.90	-1.12	-1.62
Tgif2	---	---	-1.17
Tgoln1	---	---	0.69
Thbs1	-2.60	-1.32	-3.45
Them4	---	---	1.04
Thnsl1	---	---	-0.78
Thrb	1.79	1.67	2.08
Tiam2	-0.74	---	-0.69
Ticam1	---	---	1.00
Ticrr	---	---	-0.98
Tigit	-0.90	---	---
Timeless	-0.79	---	-1.03
Tipin	-1.02	---	-1.31
Tk1	-1.21	---	-1.93
Tkt	0.71	---	0.91
Tlk2	---	---	0.76
Tm4sf4	---	---	-1.40
Tmco4	---	---	1.04
Tmem117	2.23	1.53	2.38
Tmem171	-1.60	---	-1.10
Tmem18	---	---	-0.92
Tmem194b	---	---	-1.03
Tmem2	---	---	-0.73
Tmem216	---	---	-0.76
Tmem37	1.19	1.34	1.25
Tmem38a	---	---	0.93
Tmem41b	0.85	---	1.19
Tmem55a	0.87	---	0.91
Tmem57	---	---	0.76
Tmem63b	---	---	0.71
Tmie	1.62	1.23	1.19
Tmpo	---	---	-0.87
Tmppe	0.75	---	0.69
Tnc	-0.99	-0.78	---
Tnfaip2	-0.83	---	---
Tnfaip3	-2.24	-1.44	-2.86
Tnfrsf1a	---	---	0.84
Tnfsf9	---	---	1.15
Tnnc1	1.41	---	1.85
Tns1	---	---	-1.11
Tob1	---	---	1.12

Tob2	0.67	---	0.97
Tollip	---	---	0.85
Tom1	---	---	1.16
Tom1l1	---	---	0.75
Top2a	-0.69	---	-0.99
Tor1aip2	---	---	0.75
Tor4a	---	---	-0.68
Tox	---	---	0.85
Tpx2	---	---	-0.68
Trdmt1	---	---	-0.79
Trib1	-0.86	---	-0.79
Trib3	2.39	---	3.31
Trim16	---	0.88	1.35
Trim17	0.97	---	0.86
Trim36	---	---	1.40
Trim46	---	---	1.12
Trim59	---	---	-0.76
Trim6	0.92	---	1.08
Trio	---	---	0.88
Trip10	---	---	0.92
Trmt1l	---	---	0.85
Trmt2a	---	---	-0.78
Trnt1	---	---	-0.67
Trp53bp2	---	---	0.70
Trp53inp1	1.51	---	2.05
Trp53inp2	---	---	0.91
Tsc22d1	---	---	-0.78
Tsc22d2	---	---	0.94
Tsc22d3	0.97	---	1.16
Tsfm	---	---	-0.91
Tshz1	1.12	1.12	1.48
Tsku	1.61	1.83	1.60
Tspan8	---	---	-1.01
Tspyl2	0.99	---	1.38
Ttc26	---	---	-0.79
Ttc30b	---	---	-0.79
Ttc7	-0.82	---	-0.74
Ttk	---	---	-0.71
Tuba1a	---	---	1.22
Tuba4a	0.81	0.92	1.19
Tubb2a	1.64	1.09	2.01
Tubb2b	---	---	0.78
Tubb6	-0.87	---	-0.78
Txnrd1	1.90	1.71	2.00
Ubap1	0.87	---	1.36
Ubash3b	-1.14	---	-1.02
Ubb	1.21	1.10	2.20
Ubc	1.72	1.34	3.52

Ube2h	---	---	0.75
Ube2j1	---	---	0.69
Ube2o	1.02	---	1.22
Ube4b	---	---	0.79
Ubl3	0.80	0.78	0.80
Ubqln1	---	---	0.81
Ubr4	---	---	0.87
Ubr7	---	---	-1.13
Ubtd1	---	---	0.96
Ubxn4	1.17	0.95	1.39
Ubxn8	---	---	0.65
Ugdh	1.48	1.17	1.52
Ugt1a1	1.16	0.87	0.94
Ugt1a10	1.10	0.88	0.97
Ugt1a2	1.16	0.87	0.94
Ugt1a5	1.16	0.87	0.95
Ugt1a6a	1.10	0.85	0.93
Ugt1a6b	1.09	0.87	0.96
Ugt1a7c	1.10	0.88	0.97
Ugt1a8	1.10	0.88	0.97
Ugt1a9	1.10	0.88	0.97
Ugt2b34	4.29	3.39	4.22
Uhrf1	-1.04	---	-1.17
Ulk1	0.73	---	0.95
Ung	-3.05	-0.90	-3.56
Usp1	---	---	-0.82
Usp14	---	---	0.67
Usp20	0.91	---	---
Usp22	---	---	0.96
Usp35	1.27	---	1.24
Usp43	-1.03	---	-0.89
Uspl1	---	---	0.69
Uts2b	---	---	-1.56
Vamp1	1.04	---	1.04
Vash2	---	---	0.88
Vasn	1.03	0.77	1.36
Vcam1	-1.86	-1.42	-1.96
Vegfa	2.39	1.74	3.57
Vegfc	-0.99	-0.96	-0.82
Vgll3	-0.96	---	-1.14
Vil1	-1.09	---	-1.31
Vipas39	---	---	0.78
Vldlr	1.07	---	1.16
Vnn1	1.03	---	---
Vps18	1.08	---	1.08
Vps37b	0.85	---	1.63
Vstm5	-1.17	---	-0.92
Wbp2	---	---	0.70

Wbscr27	1.40	---	0.96
Wdhd1	-0.84	---	-1.13
Wdr35	---	---	-0.78
Wdr5b	---	---	-1.27
Wdr6	-0.77	---	-1.50
Wdr76	-0.80	---	-1.14
Wdr77	---	---	-0.70
Wdr81	1.15	---	0.99
Wee1	---	---	-0.92
Whamm	0.97	---	1.12
Wipi2	---	---	0.66
Wnt10a	-0.95	---	-1.22
Wnt4	1.50	1.15	1.33
Wnt7b	-1.42	-0.75	-1.41
Wrb	---	---	-0.99
Xkr9	2.05	1.30	2.05
Xpo1	---	---	0.72
Xpot	---	---	0.65
Xrcc2	---	---	-1.06
Xrcc6	---	---	-0.69
Xrcc6bp1	---	---	-0.92
Xxylt1	---	---	-0.86
Ypel2	---	---	1.10
Ypel5	2.39	1.44	2.61
Ywhag	0.94	---	1.27
Zadh2	0.82	0.69	0.70
Zbtb10	---	---	1.14
Zbtb12	---	---	-0.96
Zbtb2	---	---	1.09
Zbtb21	---	---	1.01
Zbtb37	---	---	-0.94
Zbtb45	---	---	-0.73
Zbtb7b	---	---	0.90
Zc2hc1c	1.16	---	0.92
Zdhhc18	1.52	1.28	1.65
Zeb2	---	---	0.98
Zfand2a	1.90	1.22	3.27
Zfand5	1.43	0.89	1.68
Zfp142	---	---	1.21
Zfp185	---	---	1.14
Zfp324	---	---	-1.46
Zfp36	---	---	1.91
Zfp365	1.19	---	1.23
Zfp367	-0.88	---	-1.06
Zfp385a	---	---	0.74
Zfp39	---	---	-1.02
Zfp395	---	---	-0.86
Zfp41	---	---	-0.82

Zfp418	---	---	0.99
Zfp516	---	---	0.91
Zfp617	---	---	0.72
Zfp651	---	---	-0.89
Zfp703	---	---	0.79
Zfp867	---	---	-0.93
Zfp874a	1.00	---	0.83
Zfp874b	1.04	---	1.36
Zfp945	---	---	0.96
Zfp958	---	---	-0.88
Zfp961	---	---	-0.76
Zfpm1	---	---	1.12
Zfpm2	-1.20	---	---
Zfyve1	0.84	---	1.06
Zgrf1	---	---	-1.07
Zhx3	-0.93	---	-1.09
Zik1	---	---	-1.24
Zmynd19	---	---	-0.74
Zrsr1	2.11	1.61	2.54
Zswim4	---	---	1.01
Zswim6	---	---	1.12
Zwint	0.94	0.82	1.15
Zyx	-1.14	---	---

Supplemental Table 2. Gene expression of AML12 hepatocytes treated with truncated OxPAPC, full-length OxPAPC, and OxPAPC. Gene expression represented as log_2 of the fold change compared to vehicle. Genes regulated single treatment are highlighted in orange (truncated OxPAPC), blue (full-length OxPAPC), and purple (OxPAPC).

LPPTigr Nomenclature (previously known species)	Parent Lipid	*m/z*	Formula	LPPTigr Predicted Structure(s)
1. PAPC	---	782	$C_{44}H_{80}NO_8P$	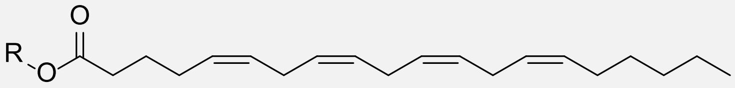
2. PLPC	---	758	$C_{42}H_{80}NO_8P$	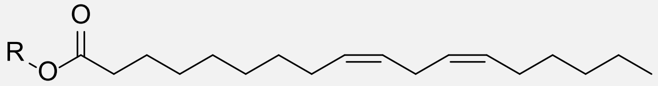
3. 16:0 LysoPC	---	496	$C_{24}H_{50}NO_7P$	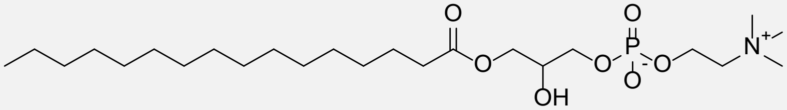
4. 18:0 LysoPC	---	524	$C_{26}H_{54}NO_7P$	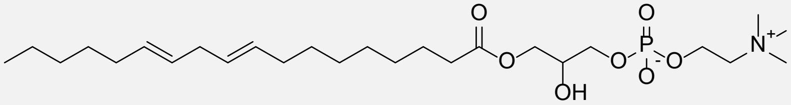
5. 18:1 LysoPC	---	522	$C_{26}H_{52}NO_7P$	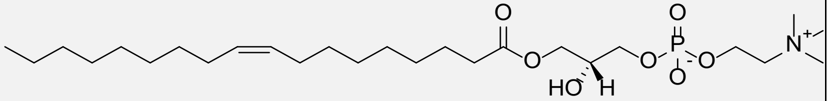
6. 18:2 LysoPC	---	520	$C_{26}H_{54}NO_7P$	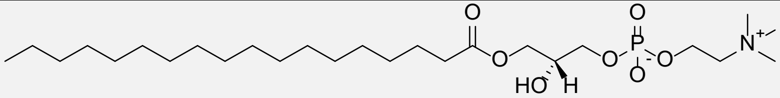
7. C37H66NO11P	PAPC	732	$C_{37}H_{66}NO_{11}P$	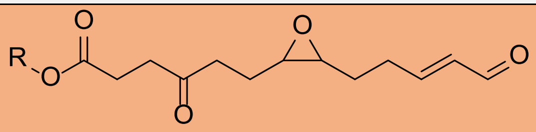 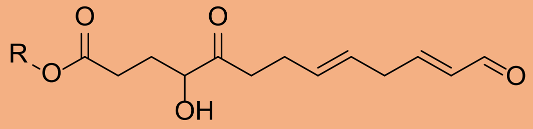 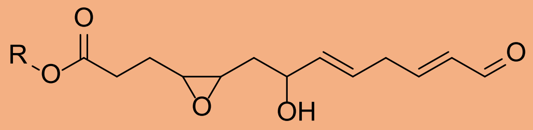 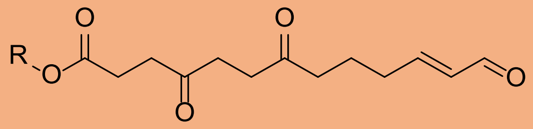 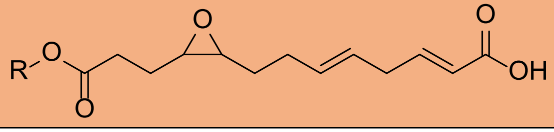

				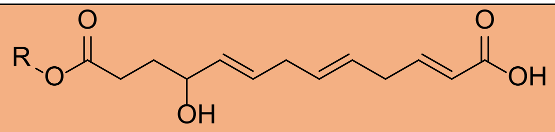 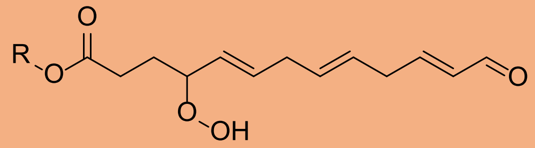 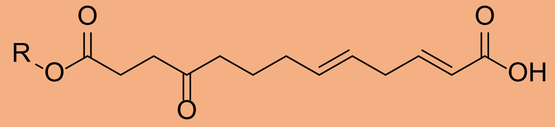 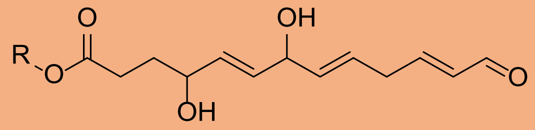 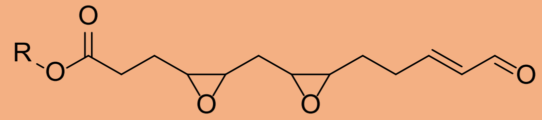
8. 16:0/7:0[1xOH,CHO]	PAPC	638	$C_{31}H_{60}NO_{10}P$	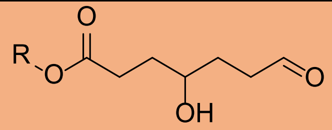
9. PONPC	PLPC	650	$C_{33}H_{64}NO_{9}P$	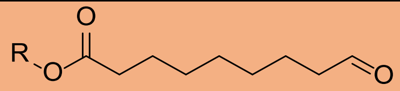
10. 16:0/12:1[CHO]	PLPC	690	$C_{36}H_{68}NO_{9}P$	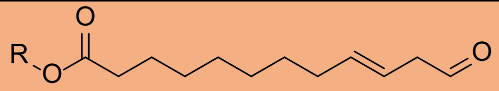
11. C34H62NO11P	PAPC	692	$C_{34}H_{62}NO_{11}P$	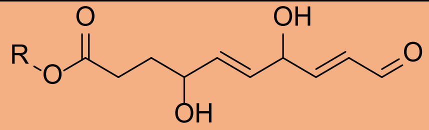 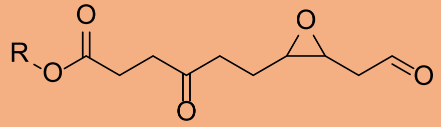 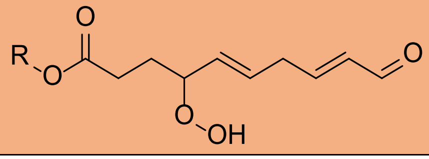

				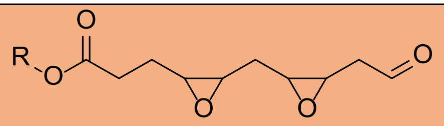 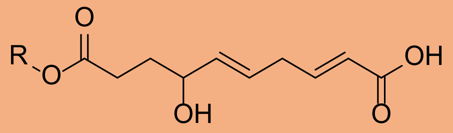 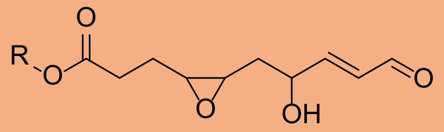 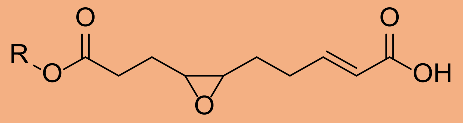 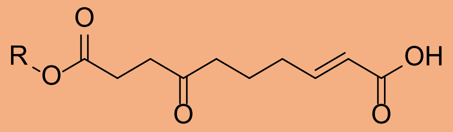 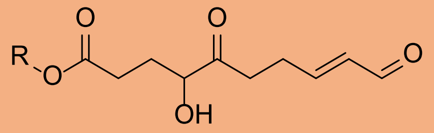 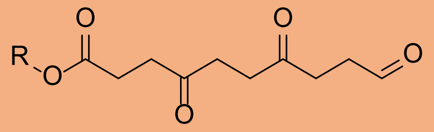
12. 16:0/11:1[CHO]	PLPC	676	$C_{35}H_{66}NO_9P$	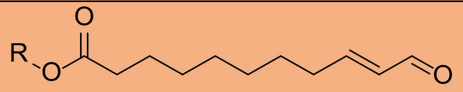
13. POBPC	PAPC	580	$C_{28}H_{54}NO_9P$	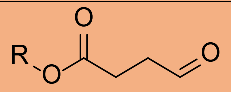
14. 16:0/8:0[CHO] (POOPC)	PLPC	636	$C_{32}H_{62}NO_9P$	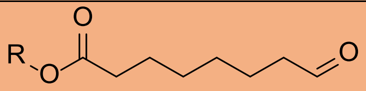
15. C35H64NO12P	PAPC	722	$C_{35}H_{64}NO_{12}P$	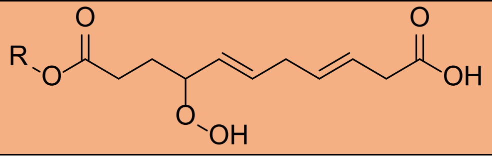

				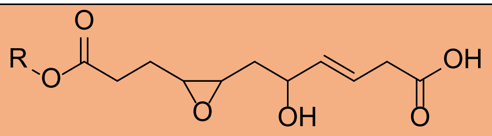<br>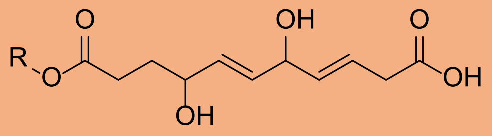<br>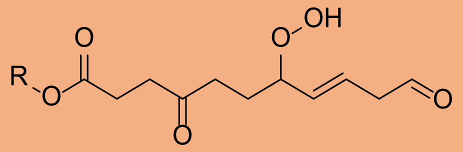<br>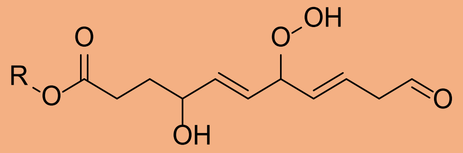<br>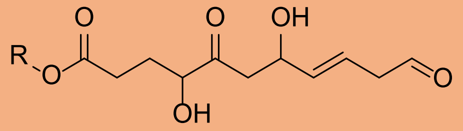<br>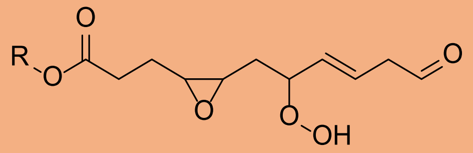<br>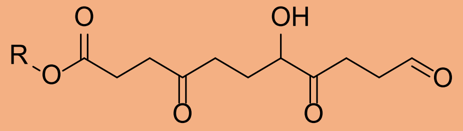<br>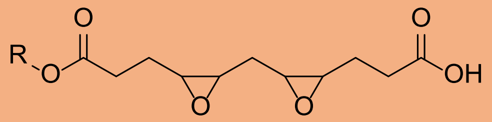<br>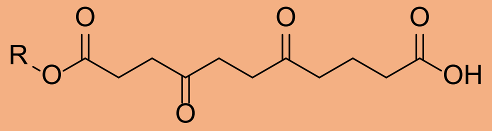<br>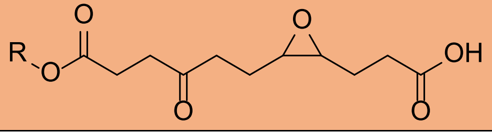

				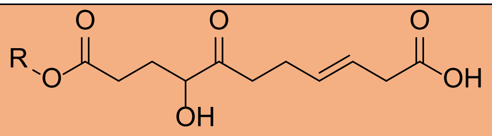 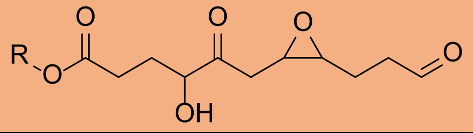
16. C35H64NO10P	PAPC	690	$C_{35}H_{64}NO_{10}P$	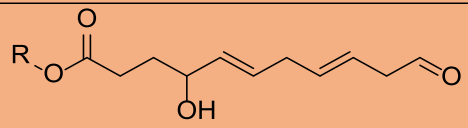 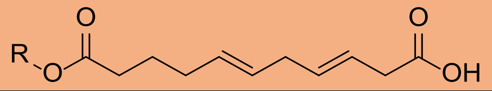
17. C35H62NO11P	PAPC	704	$C_{35}H_{62}NO_{11}P$	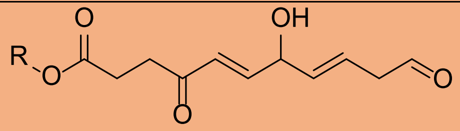 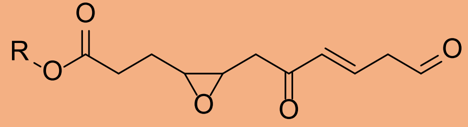 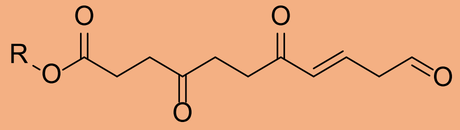 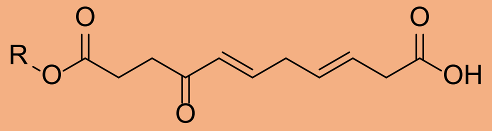
18. POVPC	PAPC	594	$C_{29}H56NO_{9}P$	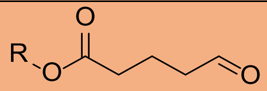
19. 16:0/10:2[CHO]	PAPC	660	$C_{34}H_{62}NO_{9}P$	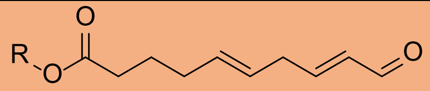
20. C36H68NO10P (HODA-PC)	PLPC	706	$C_{36}H_{68}NO_{10}P$	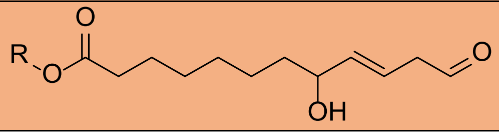

				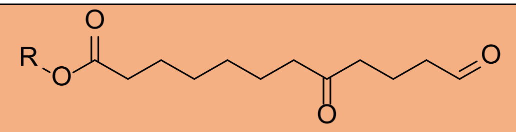 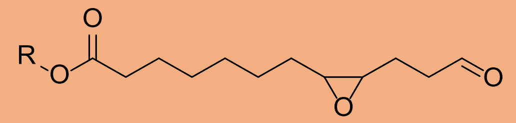 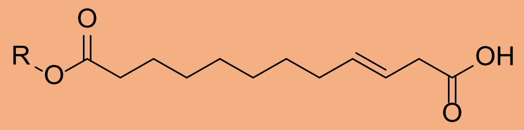
21. 16:0/7:1[CHO]	PAPC	620	$C_{31}H_{58}NO_9P$	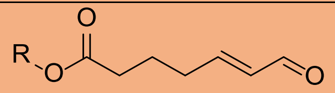
22. KOOA-PC	PAPC	648	$C_{32}H_{58}NO_{10}P$	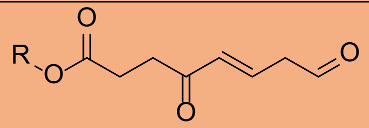
23. C32H60NO11P (HOdiA-PC)	PAPC	666	$C_{32}H_{60}NO_{11}P$	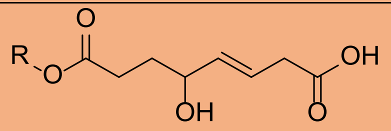 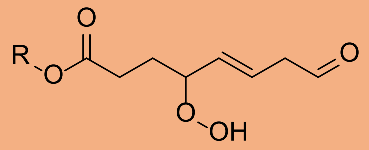 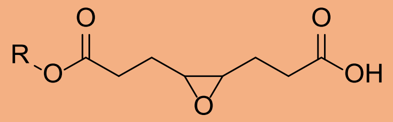 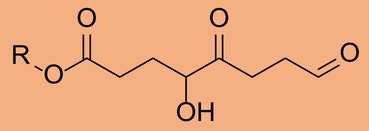 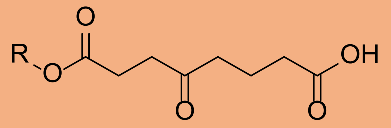
24. 16:0/10:2[1xKETO,CHO]	PAPC	674	$C_{34}H_{60}NO_{10}P$	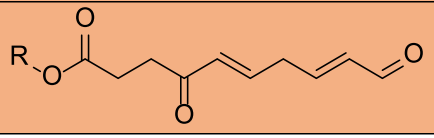

25. PazPC	PLPC	666	$C_{33}H_{64}NO_{10}P$	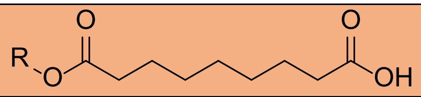
26. C32H60NO10P (HOOA-PC)	PAPC	650	$C_{32}H_{60}NO_{10}P$	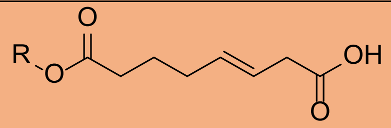 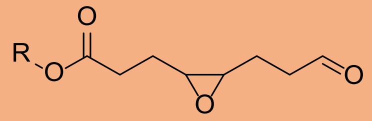 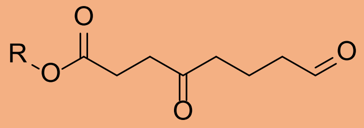 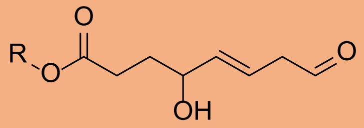
27. PGPC	PAPC	610	$C_{29}H_{56}NO_{10}P$	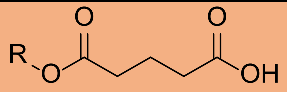
28. C42H78NO10P	PLPC	788	$C_{42}H_{78}NO_{10}P$	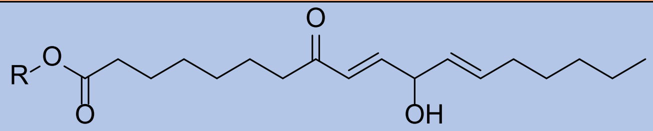 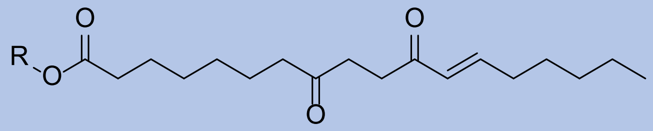 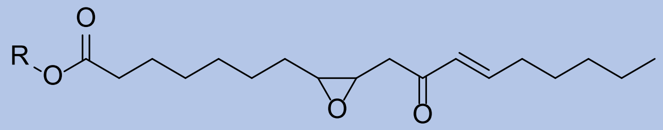
29. C42H80NO9P (HODE-PC)	PLPC	774	$C_{42}H_{80}NO_{9}P$	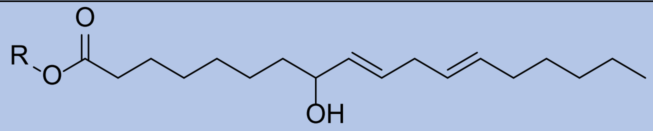 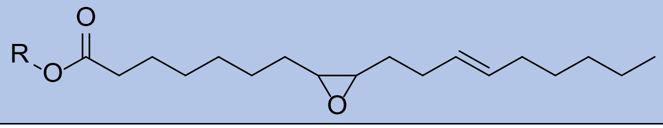

				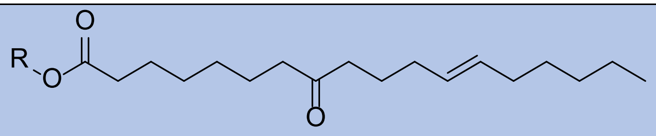
30. 16:0/20:4[2xKETO]	PAPC	810	$C_{44}H_{76}NO_{10}P$	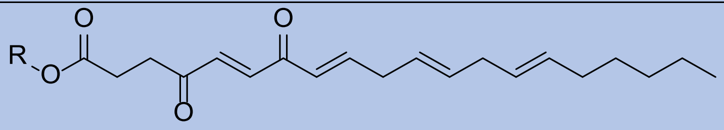
31. C44H80NO11P	PAPC	830	$C_{44}H_{80}NO_{11}P$	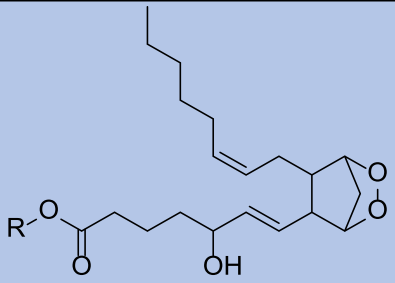 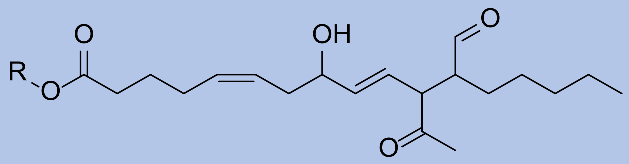 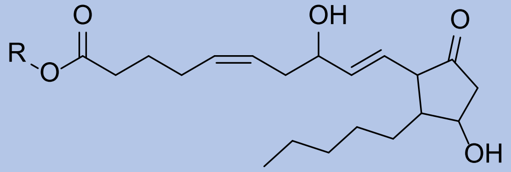 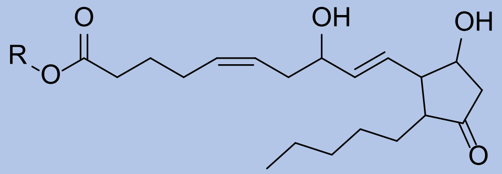 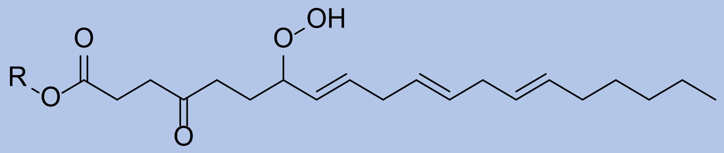 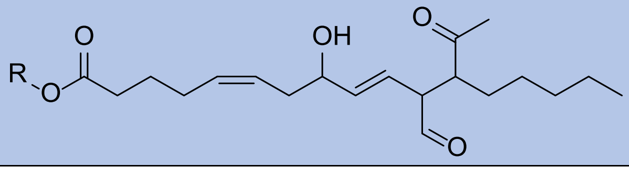

				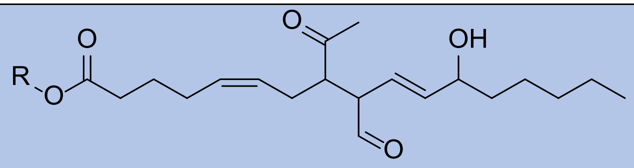 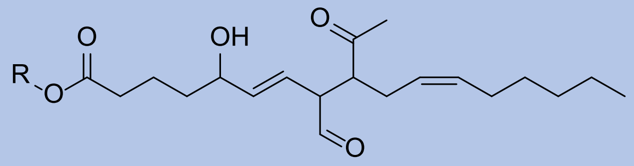 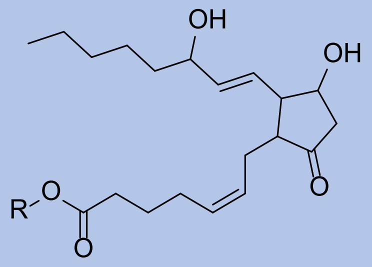 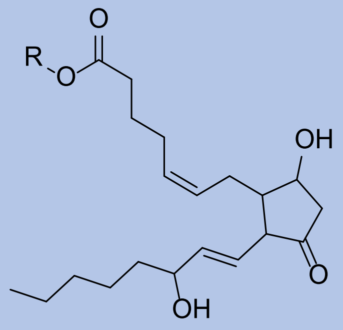 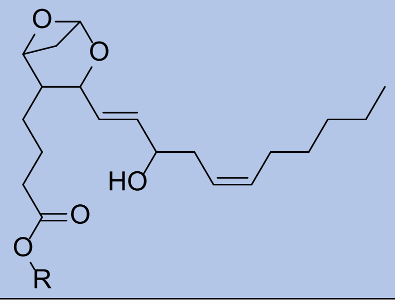

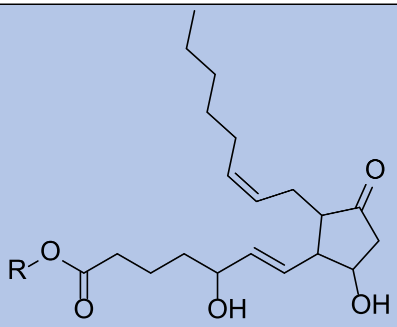

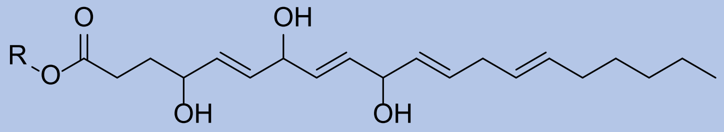

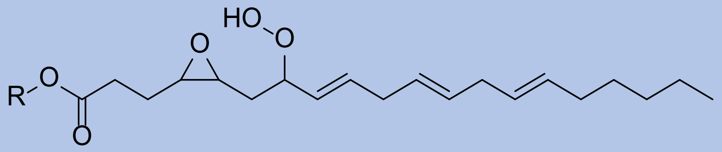

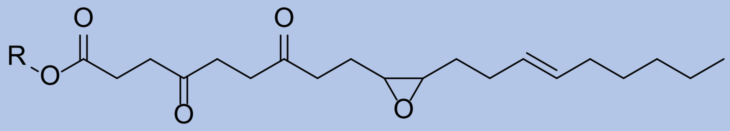

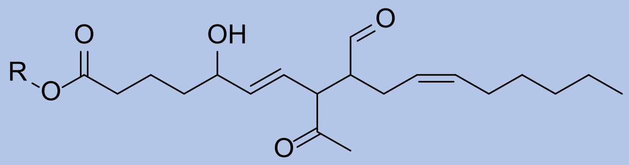

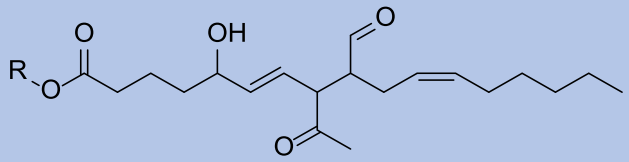

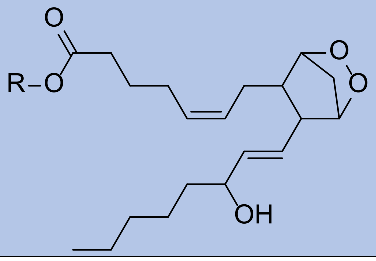

				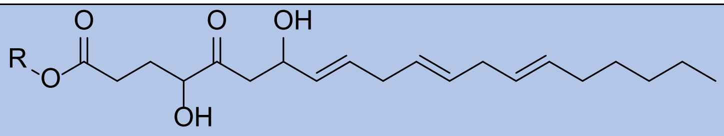<br>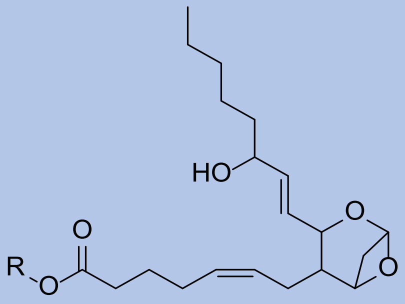<br>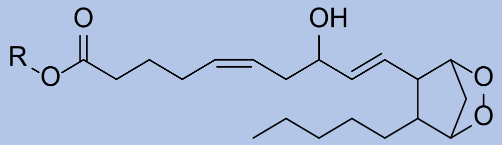<br>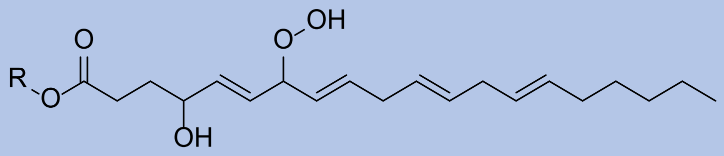<br>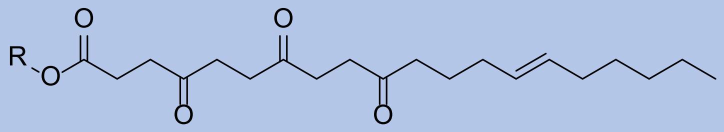<br>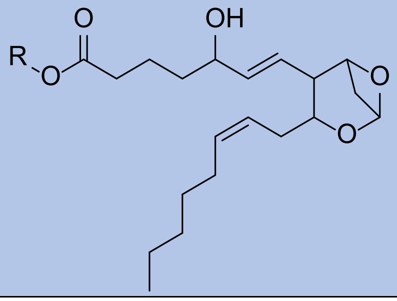

				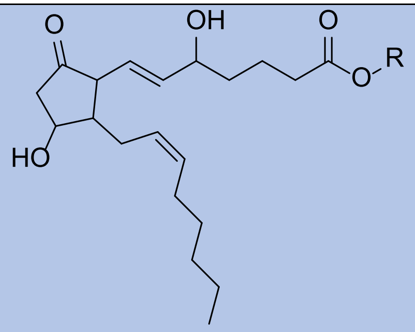<br>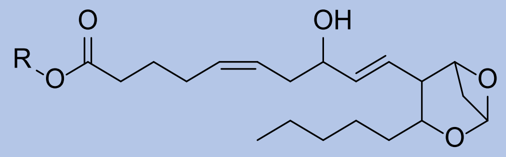<br>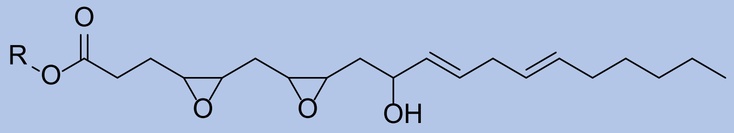<br>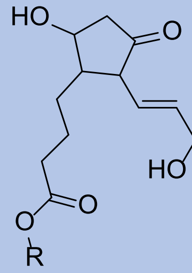<br>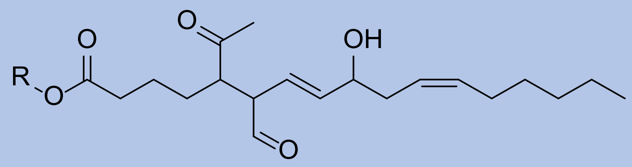<br>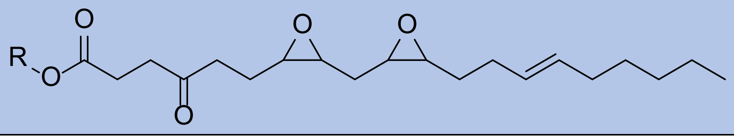

				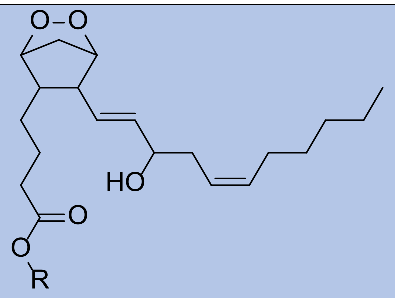 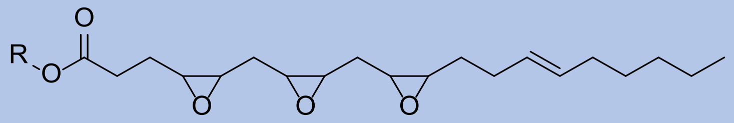 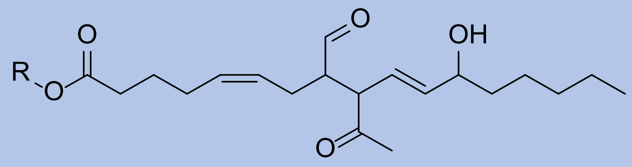 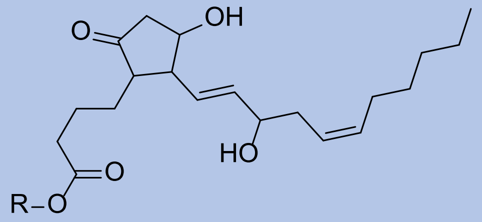 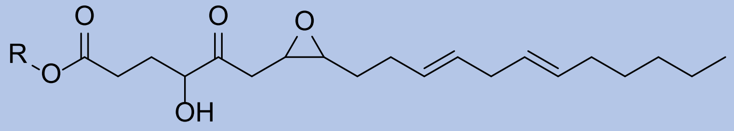 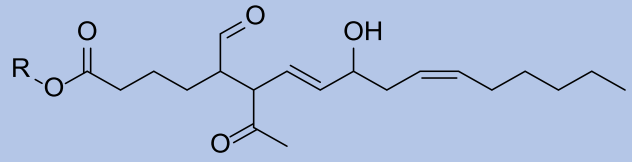 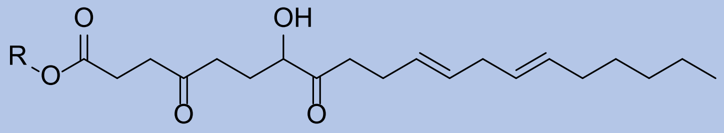
32. C44H80NO9P (HETE-PC)	PAPC	798	$C_{44}H_{80}NO_9P$	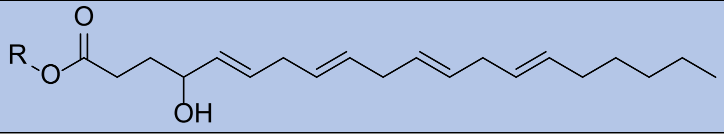

				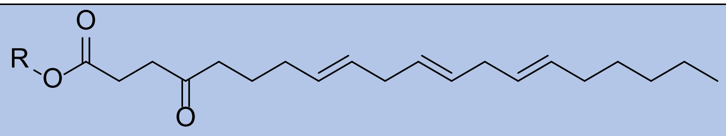 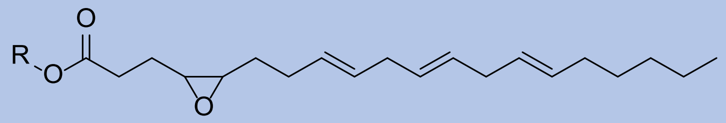
33. C42H80NO11P	PLPC	806	$C_{42}H_{80}NO_{11}P$	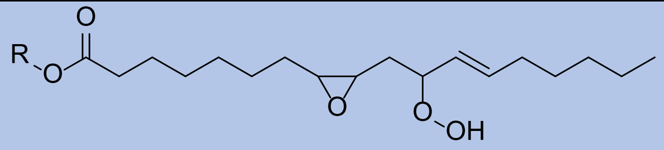 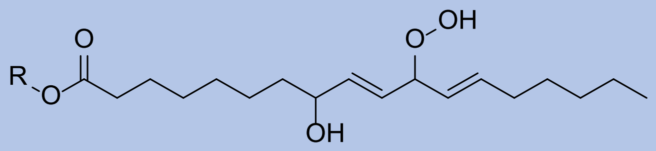 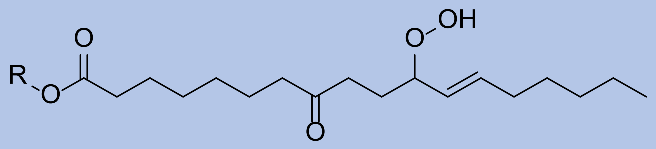
34. 16:0/18:2[1xKETO] (KODE-PC)	PLPC	772	$C_{42}H_{78}NO_{9}P$	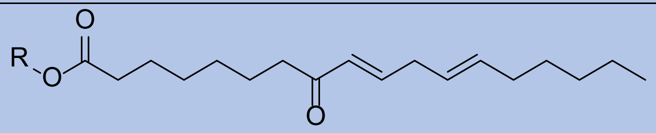
35. 16:0/18:1[1xOH] (HOME-PC)	PLPC	776	$C_{42}H_{82}NO_{9}P$	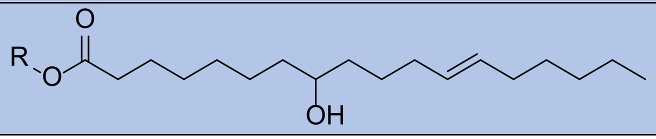

Supplemental Table 3. Oxidized phospholipids detected after development of hepatic fibrosis. *In silico* predicted structures of oxidized phospholipids derived from PAPC or PLPC detected in mouse plasma after twenty weeks on FPC diet. CHO – aldehyde, COOH – carboxylic acid, OH – hydroxy, OOH – hydroperoxy, KETO – ketone, EPOXY – epoxide.

Gene	Species	Primer orientation	Sequence
Hmox1	*Mus musculus*	Forward	ACAGCCCCACCAAGTTCAAA
		Reserve	TCTGCAGGGGCAGTATCTTG
Gclm	*Mus musculus*	Forward	TGGAGCAGCTGTATCAGTGG
		Reserve	AGAGCAGTTCTTTCGGGTCA
Pgd	*Mus musculus*	Forward	CTCCTCGACTCTGCTTCGTC
		Reserve	CGGCATCTTCTTGTCGTGTC
Acly	*Mus musculus*	Forward	TGATGGGAGAAGTTGGGAAG
		Reserve	ATCAGCTCGGGACTCAGAAA
Hmgcoas	*Mus musculus*	Forward	ACAAGCCTGACATGCTCTCC
		Reserve	TTCAGGAACATCCGAGCTAGA
Hmgcoar	*Mus musculus*	Forward	TCTTGTGGAATGCTCTGTGA
		Reserve	AAGCTCTAGGACCAGCGACA
Adamtsl2	*Mus musculus*	Forward	ATGTGAGCCCATTGGCTGTG
		Reserve	TCGGTACTTGACCACTGTGC
Timp2	*Mus musculus*	Forward	ATGGCAACCCCATCAAGAGG
		Reserve	TGGGACAGCGAGTGATCTTG
Timp1	*Mus musculus*	Forward	TCGGACCTGGTCATAAGGC
		Reserve	GTACGCCAGGGAACCAAGAA
Mmp2	*Mus musculus*	Forward	GTGTTCTTCGCAGGGAATGAG
		Reserve	GATGCTTCCAAACTTCACGCT
Itga8	*Mus musculus*	Forward	ACACGTTCCTCAAGAGAAAGAA
		Reserve	GGAGTGGCCCAAATAACCGA
Col15a1	*Mus musculus*	Forward	CTGTCCACTTTCCGAGCCTTT
		Reserve	AAAGCACTTGGCCCTTGAGA

Col8a1	*Mus musculus*	Forward	GGCAAAGAGTACCCACACCTACC
		Reserve	GACCTTGTTCTCCGCGCAAACTG
Col4a2	*Mus musculus*	Forward	TCGTTCAGCCAGGTTGCATT
		Reserve	AAAGCCCTTGAGCCCTTGTT
Col5a2	*Mus musculus*	Forward	TGGGGACTGATGGTACACCT
		Reserve	GGATCACCCGATTGTCCTCG
Adam8	*Mus musculus*	Forward	TGAACAAGCAGCGTCTACGA
		Reserve	CTGGGAGTGGTGAACTGGAC
Fgfr1	*Mus musculus*	Forward	TCCCTGTGGAAGTGGAGTCT
		Reserve	GCTACAGGCCTACGGTTTGG
Tgfbr1	*Mus musculus*	Forward	GGCGAAGGCATTACAGTGTT
		Reserve	TGGTGAATGACAGTGCGGTT
Itga9	*Mus musculus*	Forward	GCTCTCGCTGTAGCCCATC
		Reserve	ACCCACGAGGACCCAGC
Col6a1	*Mus musculus*	Forward	AGGGCTACAAGGAACCATGC
		Reserve	TTTCCTCGCTCCCCCTCATA
Tgfbr2	*Mus musculus*	Forward	CCAAGTCGGATGTGGAAATGG
		Reserve	TGTCGCAAGTGGACAGTCTC
Col14a1	*Mus musculus*	Forward	TGAAGCACCCACAGCCATAG
		Reserve	TCCAGGCACCATAACCACTTC
Itga1	*Mus musculus*	Forward	TCAGTGGAGAGCAGATCGGA
		Reserve	CCCACAGGGCTCATTCTTGT
Adam9	*Mus musculus*	Forward	GGGCCGACGTATAATGCAAAG
		Reserve	CAGGTGGCGGTCTGGAG
CypA	*Mus musculus*	Forward	CGATGACGAGCCCTTGG

		Reserve	TCTGCTGTCTTTGGAACTTTGTC
B2m	*Mus musculus*	Forward	ATTCACCCCCACTGAGACTG
		Reserve	TGCTATTTCTTTCTGCGTGC
Hmox1	*Homo sapiens*	Forward	AAGACTGCGTTCCTGCTCAAC
		Reserve	AAAGCCCTACAGCAACTGTCG
Gclm	*Homo sapiens*	Forward	GCGAGGAGCTTCATGATTGT
		Reserve	TGTGCAACTCCAAGGACTGA
Hprt	*Homo sapiens*	Forward	AGGCGAACCTCTCGGCTTTC
		Reserve	CAAGACGTTCAGTCCTGTCCATA
scFv-E06	*N/A*	Forward	GTACTGCTGCTCTGGGTTCC
		Reserve	CACTGGCCGTGCAACTAATG

Supplemental Table 4. Forward and reserve primer sequences for cDNA and genomic DNA amplification.

Data File S1. Source data.
